# Ability of bifidobacteria to metabolize chitin-glucan and its impact on the gut microbiota

**DOI:** 10.1038/s41598-019-42257-z

**Published:** 2019-04-08

**Authors:** Giulia Alessandri, Christian Milani, Sabrina Duranti, Leonardo Mancabelli, Thibaut Ranjanoro, Salvatore Modica, Luca Carnevali, Rosario Statello, Francesca Bottacini, Francesca Turroni, Maria Cristina Ossiprandi, Andrea Sgoifo, Douwe van Sinderen, Marco Ventura

**Affiliations:** 1 Department of Veterinary Medical Science, University of Parma, Parma, Italy; 2 Laboratory of Probiogenomics, Department of Chemistry, Life Sciences, and Environmental Sustainability, University of Parma, Parma, Italy; 3 KitoZyme, Herstal, Belgium; 4 Stress Physiology Laboratory, Department of Chemistry, Life Sciences and Environmental Sustainability, University of Parma, Parma, Italy; 5 APC Microbiome Institute and School of Microbiology, Bioscience Institute, National University of Ireland, Cork, Ireland

## Abstract

Chitin-glucan (CG) represents a natural carbohydrate source for certain microbial inhabitants of the human gut and may act as a prebiotic for a number of bacterial taxa. However, the bifidogenic activity of this substrate is still unknown. In the current study, we evaluated the ability of chitin-glucan to influence growth of 100 bifidobacterial strains belonging to those species commonly identified within the bifidobacterial communities residing in the infant and adult human gut. Such analyses were coupled with transcriptome experiments directed to explore the transcriptional effects of CG on *Bifidobacterium breve* 2L, which was shown to elicit the highest growth performance on this natural polysaccharide. In addition, an *in vivo* trial involving a rat model revealed how the colonization efficiency of this bifidobacterial strain was enhanced when the animals were fed with a diet containing CG. Altogether our analyses indicate that CG is a valuable novel prebiotic compound that may be added to the human diet in order to re-establish/reinforce bifidobacteria colonization in the mammalian gut.

## Introduction

Chitin-glucan (CG) is a high-purity bio-polymer composed of two distinct polysaccharides represented by chitin (β-1,4-poly-N-acetyl-D-glucosamine) and β-1,3-D-glucan in a ratio ranging from 20:80 to 40:60 (w/w)^1^. CG is typically extracted from *Aspergillus niger* mycelial cell walls. Furthermore, another natural sources of CG are represented by different fungal species and yeasts due to the presence of this bio-polymer in their inner cell walls^2,3^. Recently, CG has been indicated as food supplements fixing the maximum consumption rate to five gr per day for an average person^4^.

However, mammalian enzymes are unable to degrade CG and therefore following ingestion this glycopolymer will arrive in its intact form in the large intestine where it may influence growth and/or metabolic activity of different members of the gut microbiota. In this context, there is growing scientific evidence of possible prebiotic effects elicited by CG towards various microorganisms of the mammalian gut^5,6^. Recently, an *in vitro* study performed in a dynamic gut simulator (SHIME) illustrated the effect of CG on microbiota composition and activity, leading to a decrease of the Firmicutes/Bacteroidetes ratio and an increase in the relative abundance of *Roseburia* spp^1^, which could suggest a potential role exploited by CG in shaping the gut microbiota through a prebiotic effect as previously displayed for other complex carbohydrates^7^.

The term ‘prebiotic’ includes compounds, such as non-digestible (i.e. cannot be metabolized or degraded by the host) carbohydrates, that are selectively metabolized by beneficial gut bacteria. Prebiotic treatment is a dietary strategy by which the gastrointestinal microbiota can be modified, both in composition and/or activity, for the purpose of conferring health benefits to the host^8^. In fact, it has been demonstrated that prebiotics can reduce symptoms associated with inflammatory bowel disease, the duration of infectious and antibiotic-associated diarrhea, and the risk of cardiovascular diseases. Furthermore, prebiotics may also promote satiety and weight loss, preventing obesity and enhancing protective effects against the onset of colon cancer^9^.

Bifidobacteria are very prevalent and abundant human gut microbiota members, especially during the first months following birth^10^, though their numbers decrease following weaning and in elderly. Bifidobacterial abundance in the human microbiota is markedly reduced following gastrointestinal diseases^11^, suggesting that this taxon plays a positive role in the promotion of host health^12^. Prebiotics, such as dietary fibers, have been used to counteract the reduction of bifidobacterial abundance in the human gut^13^.

So far, a very limited scientific evidence is available regarding the possible prebiotic effect of CG towards members of the *Bifidobacterium* genus^6^. In the current report, we evaluated possible bifidogenic features of CG towards 100 different bifidobacterial strains using an *in vitro* approach. Such analyses revealed the presence of a highly CG-responsive bifidobacterial strain, *Bifidobacterium breve* 2L, whose transcriptome when cultivated on CG was investigated in detail by RNAseq experiments. Furthermore, *in vivo* trials in a rat model fed with CG and *B. breve* 2L clearly demonstrated that CG increases the abundance of *B. breve* 2L and several other members of the mammalian gut microbiota.

## Results and Discussion

### Evaluation of prebiotic capability of chitin-glucan toward bifidobacteria

**Figure 1:**
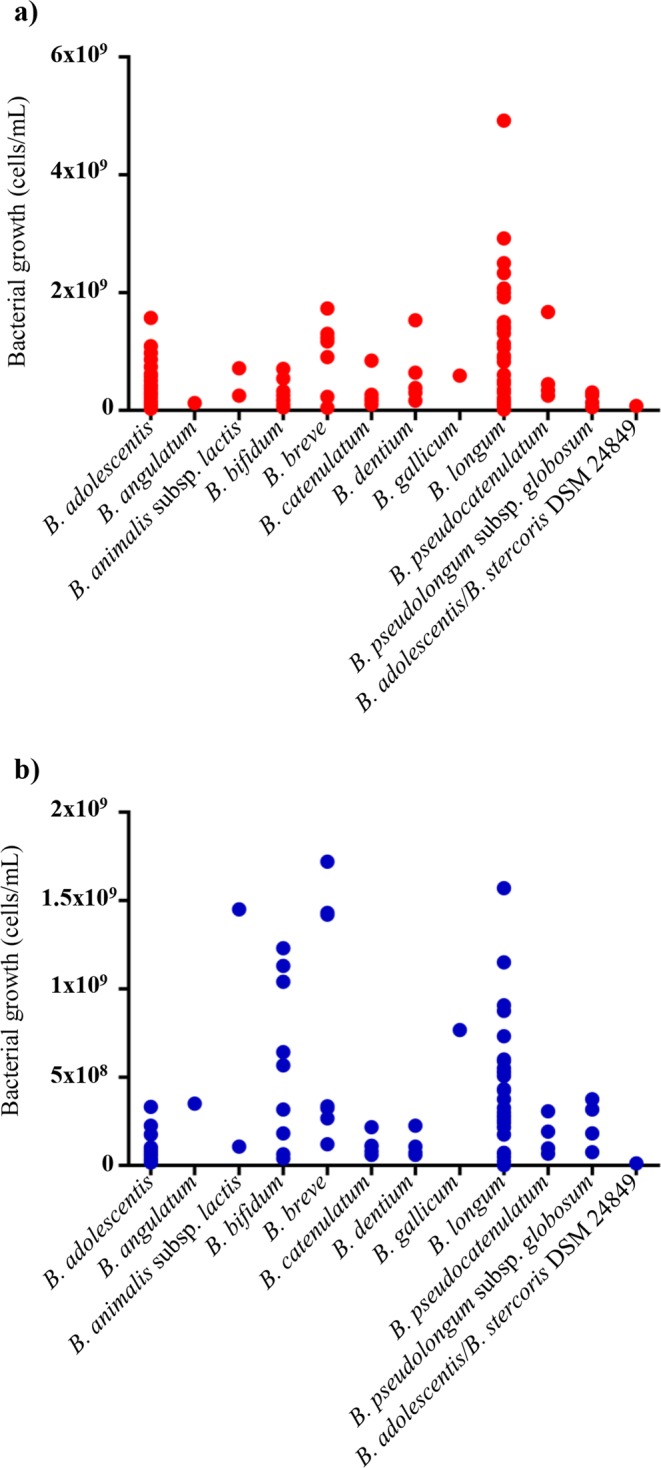
Bifidobacterial growth in MRS and chitin-glucan. The dispersion graphs report bacterial growth for the different bifidobacterial species included in this study, expressed as cells/mL. Panel a shows the growths in complete MRS (in red), while panel b depicts growths in MRS w/o glu + CG (in blue). Each growth reported is the average value of three experiments.

One hundred bifidobacterial strains previously isolated from the human environment (reported in Table 1), were evaluated for their ability to grow on CG, as unique carbon source. These strains were isolated from infant feces (*Bifidobacterium bifidum, Bifidobacterium breve* and *Bifidobacterium pseudocatenulatum*) as well as from gastrointestinal tract of adult (*Bifidobacterium adolescentis, Bifidobacterium angulatum, Bifidobacterium catenulatum, Bifidobacterium dentium, Bifidobacterium longum*, *Bifidobacterium pseudolongum* subsp. *globosum*). In addition, the bifidobacterial collection used in this project included strains belonging to *Bifidobacterium animalis* subsp. *lactis*, isolated from various commercial products sold as probiotics. Despite the fact that CG consists of two different polysaccharides, i.e. chitin (β-1,4-poly-N-acetyl-D-glucosamine) and β-1,3-D-glucan, we focused our study on the biopolymer CG due to its commercial relevance as an extract of *Aspergillus niger*. Furthermore, we have used the Tyndallization procedure, which operates a series moderate heat treatment steps separated by rapid cooling stages, in order to achieve a sterilization of CG-based medium without causing extensive hydrolysis of CG as might occur when using high-level temperature treatments such as autoclaving. Growth assays were conducted on a modified MRS growth media, i.e. MRS w/o glu + CG (0.5% (w/v) together with a positive control (complete MRS) as well as a negative control (MRS w/o glu). Experiments were performed in triplicates for each growth media. We observed a minimal growth also on MRS w/o glu (≤10^3^ cells/ml), which is probably linked to the presence of components such as meat and yeast extract providing extra organic carbon sources. However, the final cell count of positive control and MRS w/o glu + CG were calculated by subtracting the cells count obtained on MRS w/o glu. Interestingly, all strains were shown to exhibit growth using MRS w/o glu + CG as the unique carbon source. Remarkably, almost all assayed strains displayed growth levels comparable with those obtained in complete MRS (MRS + glucose), i.e. comprised between 10^7^–10^9^ cells/mL (Table 1). Notably, this may be caused by partial degradation of CG in simpler sugars, e.g. glucose monomers from glucan, due to the heat treatment included in the modified Tyndallization approach employed to sterilize the MRS w/o glu + CG medium. Only in case of strains belonging to the *B. adolescentis* and *B. longum* species, the observed growth levels were significantly different (p-value < 0.05) (Fig. 1). In detail, *B. adolescentis* strains exhibited a final cell count of 7.59 × 10^7^ cells/mL, and 4.19 × 10^8^ cells/mL, in MRS w/o glu + CG and in complete MRS, respectively. Regarding *B. longum*, these strains were able to reach an average final cell count of 4.40 × 10^8^ cells/mL in MRS w/o glu + CG, which is a lower cell yield compared to the final cell count in complete MRS (1.05 × 10^9^ cells/mL). This finding indicates that these bifidobacterial species have a reduced ability to utilize CG as compared to the other assessed bifidobacterial strains. In contrast, *B. breve* and *B. bifidum* strains were shown to reach the highest cell numbers on this substrate (cell numbers ranging from 10^8^ to 10^9^ cells/mL), and in some cases surpassing growth yields obtained in complete MRS (Fig. 1). These two bacterial species are typically isolated from infants, and consequently expected to be metabolically adapted to degrade host-specific glycans such as mucin and host glycan constituents like N-acetylglucosamine^14,15^. Notably, CG is partially composed of polymerized β-1,4-poly-N-acetyl-D-glucosamine, which may explain the reason for the more robust metabolic efficiency of these species toward CG (compared to the other examined bifidobacterial species).

**Table 1:** Bifidobacterial strains grown on MRS medium and MRS w/o glucose supplemented with CG and results of *in vitro* experiments.

Strains^a^	Origin	Average (cells/mL)	SD	Average CG (cells/mL)	CG SD	Average Agar plate (CFU/mL)	Agar plate SD
*B. adolescentis* 1901B	Adult stool sample	1.03 × 10^8^	4.63 × 10^7^	3.42 × 10^7^	2.67 × 10^7^		
*B. adolescentis* 1902B	Adult stool sample	6.17 × 10^8^	2.08 × 10^8^	2.92 × 10^7^	8.04 × 10^6^		
*B. adolescentis* 1903B	Adult stool sample	2.75 × 10^8^	1.52 × 10^8^	4.75 × 10^7^	2.88 × 10^7^		
*B. adolescentis* 1904B	Adult stool sample	2.50 × 10^8^	2.38 × 10^8^	3.33 × 10^8^	3.64 × 10^8^	8.40 × 10^6^	2.06 × 10^6^
*B. adolescentis* 22L	Human milk	3.67 × 10^8^	1.94 × 10^8^	2.83 × 10^7^	1.18 × 10^7^		
*B. adolescentis* 236B	Colonoscopic sample	2.17 × 10^8^	1.59 × 10^8^	4.00 × 10^7^	4.33 × 10^6^		
*B. adolescentis* 382B	Adult stool sample	5.08 × 10^8^	3.13 × 10^8^	7.17 × 10^7^	9.46 × 10^6^		
*B. adolescentis* 388B	Adult stool sample	1.17 × 10^8^	5.20 × 10^7^	2.26 × 10^8^	1.13 × 10^8^	3.75 × 10^7^	8.57 × 10^6^
*B. adolescentis* 42B	Adult stool sample	2.75 × 10^8^	8.66 × 10^7^	1.83 × 10^7^	1.04 × 10^7^		
*B. adolescentis* 487B	Adult stool sample	4.08 × 10^8^	1.89 × 10^8^	3.83 × 10^7^	1.89 × 10^7^		
*B. adolescentis* 532B	Colonoscopic sample	3.83 × 10^8^	1.23 × 10^8^	1.02 × 10^8^	2.25 × 10^7^		
*B. adolescentis* 548B	Colonoscopic sample	2.67 × 10^8^	3.82 × 10^7^	6.00 × 10^7^	1.73 × 10^7^		
*B. adolescentis* 59B	Adult stool sample	3.08 × 10^8^	8.04 × 10^7^	2.50 × 10^7^	4.33 × 10^6^		
*B. adolescentis* 61B	Adult stool sample	1.58 × 10^8^	1.44 × 10^7^	2.25 × 10^7^	1.98 × 10^7^		
*B. adolescentis* 65B	Colonoscopic sample	5.75 × 10^8^	1.95 × 10^8^	4.50 × 10^7^	1.09 × 10^7^		
*B. adolescentis* 679B	Adult stool sample	1.75 × 10^8^	5.00 × 10^7^	6.00 × 10^7^	6.61 × 10^6^		
*B. adolescentis* 703B	Adult stool sample	9.75 × 10^8^	5.91 × 10^8^	5.82 × 10^7^	4.04 × 10^7^		
*B. adolescentis* 70B	Adult stool sample	4.33 × 10^8^	1.76 × 10^8^	2.25 × 10^7^	1.41 × 10^7^		
*B. adolescentis* 711B	Colonoscopic sample	8.67 × 10^8^	5.20 × 10^7^	1.75 × 10^8^	1.00 × 10^8^		
*B. adolescentis* 723B	Colonoscopic sample	1.57 × 10^9^	4.15 × 10^8^	8.42 × 10^7^	7.88 × 10^7^		
*B. adolescentis* 731B	Colonoscopic sample	7.42 × 10^8^	1.38 × 10^8^	9.92 × 10^7^	5.43 × 10^7^		
*B. adolescentis* 734B	Colonoscopic sample	3.17 × 10^7^	1.44 × 10^7^	1.13 × 10^7^	5.30 × 10^6^		
*B. adolescentis* ATCC 15703	Colonoscopic sample	7.33 × 10^7^	2.50 × 10^7^	2.17 × 10^7^	5.20 × 10^6^		
*B. adolescentis* LMG 10733	Colonoscopic sample	1.09 × 10^9^	3.17 × 10^8^	5.33 × 10^7^	3.82 × 10^6^		
*B. adolescentis* LMG 10734	Colonoscopic sample	1.27 × 10^8^	3.16 × 10^7^	6.00 × 10^7^	0		
*B. adolescentis* LMG 18897	Adult stool sample	5.83 × 10^8^	1.81 × 10^8^	2.50 × 10^7^	7.07 × 10^6^		
*B. angulatum* LMG 11039	Adult stool sample	1.25 × 10^8^	6.61 × 10^7^	3.50 × 10^8^	3.68 × 10^8^		
*B. animalis* subsp. *lactis* Bb-12	Fermented milk product	7.17 × 10^8^	4.25 × 10^8^	1.45 × 10^9^	2.78 × 10^8^	8.30 × 10^6^	2.43 × 10^6^
*B. animalis* subsp. *lactis* DSM 10140	Fermented milk	2.50 × 10^8^	4.33 × 10^7^	1.08 × 10^8^	5.20 × 10^7^		
*B. bifidum* 156B	Infant stool sample	2.58 × 10^8^	1.66 × 10^8^	6.42 × 10^8^	8.78 × 10^7^	2.61 × 10^6^	2.75 × 10^5^
*B. bifidum* 324B	Infant stool sample	1.67 × 10^8^	1.04 × 10^8^	1.83 × 10^8^	5.20 × 10^7^	2.58 × 10^6^	4.15 × 10^5^
*B. bifidum* 361B	Adult stool sample	5.00 × 10^7^	2.50 × 10^7^	4.00 × 10^7^	1.15 × 10^7^		
*B. bifidum* 85B	Infant stool sample	3.25 × 10^8^	1.25 × 10^8^	3.17 × 10^8^	2.89 × 10^7^		
*B. bifidum* LMG 11041	Infant stool sample	1.83 × 10^8^	1.44 × 10^8^	4.92 × 10^7^	1.44 × 10^6^	8.14 × 10^6^	9.27 × 10^5^
*B. bifidum* LMG 11582	Adult stool sample	5.42 × 10^8^	8.04 × 10^7^	5.67 × 10^8^	1.42 × 10^8^	1.62 × 10^7^	4.55 × 10^6^
*B. bifidum* LMG 11583	Adult stool sample	2.08 × 10^8^	7.64 × 10^7^	1.23 × 10^9^	3.44 × 10^8^	5.00 × 10^6^	5.35 × 10^5^
*B. bifidum* LMG 13195	Infant colonoscopic sample	9.58 × 10^7^	2.67 × 10^7^	6.42 × 10^7^	5.20 × 10^6^		
*B. bifidum* LMG 13200	Infant stool sample	7.08 × 10^8^	3.83 × 10^8^	1.04 × 10^9^	1.01 × 10^8^		
*B. bifidum* PRL2010	Infant stool sample	5.42 × 10^8^	2.40 × 10^8^	1.13 × 10^9^	1.95 × 10^8^	4.15 × 10^7^	4.74 × 10^6^
*B. breve* 12L	Human milk	1.30 × 10^9^	1.26 × 10^9^	1.43 × 10^9^	1.32 × 10^8^	7.43 × 10^7^	6.56 × 10^6^
*B. breve* 2L	Human milk	1.23 × 10^9^	5.13 × 10^8^	1.42 × 10^9^	7.52 × 10^8^	1.33 × 10^8^	2.74 × 10^7^
*B. breve* 31L	Human milk	1.17 × 10^9^	5.77 × 10^8^	3.25 × 10^8^	6.61 × 10^7^	2.09 × 10^4^	1.10 × 10^4^
*B. breve* 687B	Adult stool sample	1.55 × 10^9^	1.42 × 10^8^	1.46 × 10^9^	1.68 × 10^8^		
*B. breve* 689B	Adult stool sample	1.73 × 10^9^	1.95 × 10^8^	2.68 × 10^8^	7.39 × 10^7^		
*B. breve* 691B	Adult stool sample	9.08 × 10^8^	1.26 × 10^8^	1.72 × 10^9^	2.88 × 10^8^	1.11 × 10^8^	5.13 × 10^6^
*B. breve* LMG 13208	Infant colonoscopic sample	2.33 × 10^8^	1.44 × 10^7^	3.35 × 10^8^	9.84 × 10^7^	4.34 × 10^7^	8.40 × 10^6^
*B. catenulatum* 1231B	Human gut	1.00 × 10^8^	5.00 × 10^7^	6.00 × 10^7^	5.00 × 10^6^		
*B. catenulatum* 1232B	Human gut	1.67 × 10^8^	8.04 × 10^7^	1.09 × 10^8^	1.91 × 10^7^		
*B. catenulatum* 1233B	Human gut	2.67 × 10^8^	1.42 × 10^8^	1.13 × 10^8^	3.03 × 10^7^		
*B. catenulatum* 1234B	Human gut	2.08 × 10^8^	1.46 × 10^8^	8.00 × 10^7^	2.70 × 10^7^		
*B. catenulatum* LMG 11043	Colonoscopic sample	8.48 × 10^8^	1.49 × 10^8^	2.17 × 10^8^	1.18 × 10^8^		
*B. dentium* 125B	Adult stool sample	1.53 × 10^9^	4.04 × 10^8^	2.25 × 10^8^	1.25 × 10^8^		
*B. dentium* 181B	Adult stool sample	3.83 × 10^8^	1.53 × 10^8^	7.33 × 10^7^	2.84 × 10^7^		
*B. dentium* 183B	Adult stool sample	3.00 × 10^8^	1.75 × 10^8^	6.08 × 10^7^	1.66 × 10^7^		
*B. dentium* 369B	Adult stool sample	6.42 × 10^8^	2.45 × 10^8^	1.08 × 10^8^	2.60 × 10^7^		
*B. dentium* LMG 11405	Oral cavity	1.67 × 10^8^	1.26 × 10^8^	6.67 × 10^7^	1.44 × 10^7^	3.19 × 10^6^	2.75 × 10^6^
*B. gallicum* LMG 11596	Colonoscopic sample	5.92 × 10^8^	2.31 × 10^8^	7.67 × 10^8^	2.93 × 10^8^	5.72 × 10^7^	7.25 × 10^6^
*B. longum* 123B	Colonoscopic sample	1.50 × 10^9^	9.01 × 10^8^	4.33 × 10^8^	1.38E × 10^8^		
*B. longum* 134B	Colonoscopic sample	1.33 × 10^8^	7.64 × 10^7^	1.75 × 10^8^	7.50 × 10^7^	1.85 × 10^7^	6.03 × 10^6^
*B. longum* 159B	Colonoscopic sample	1.58 × 10^8^	5.20 × 10^7^	2.58 × 10^8^	8.04 × 10^7^	1.77 × 10^6^	7.33 × 10^5^
*B. longum* 207B	Colonoscopic sample	1.67 × 10^7^	1.42 × 10^7^	6.67 × 10^7^	2.27 × 10^7^	1.86 × 10^7^	3.96 × 10^6^
*B. longum* 220B	Colonoscopic sample	3.08 × 10^8^	1.66 × 10^8^	3.00 × 10^8^	2.84 × 10^8^		
*B. longum* 224B	Colonoscopic sample	2.00 × 10^9^	4.33 × 10^8^	1.57 × 10^9^	2.25 × 10^8^		
*B. longum* 229B	Colonoscopic sample	4.92 × 10^9^	2.16 × 10^9^	9.08 × 10^8^	2.01 × 10^8^		
*B. longum* 296B	Adult stool sample	1.42 × 10^9^	1.18 × 10^9^	6.00 × 10^8^	1.75 × 10^8^		
*B. longum* 314B	Colonoscopic sample	2.08 × 10^8^	5.20 × 10^7^	2.50 × 10^8^	0	1.78 × 10^7^	3.30 × 10^6^
*B. longum* 319B	Colonoscopic sample	3.50 × 10^8^	3.04 × 10^8^	5.25 × 10^8^	1.89 × 10^8^	3.53 × 10^7^	1.74 × 10^7^
*B. longum* 340B	Adult stool sample	8.92 × 10^8^	1.46 × 10^8^	2.17 × 10^8^	5.20 × 10^7^		
*B. longum* 346B	Adult stool sample	3.33 × 10^8^	2.13 × 10^8^	3.25 × 10^8^	1.50 × 10^8^		
*B. longum* 350B	Adult stool sample	4.58 × 10^8^	1.44 × 10^7^	2.42 × 10^8^	1.01 × 10^8^		
*B. longum* 351B	Adult stool sample	1.42 × 10^8^	7.64 × 10^7^	7.17 × 10^7^	3.82 × 10^6^		
*B. longum* 397B	Adult stool sample	1.07 × 10^9^	7.51 × 10^7^	5.25 × 10^8^	1.15 × 10^8^		
*B. longum* 419B	Adult stool sample	5.00 × 10^8^	1.32 × 10^8^	4.25 × 10^8^	1.52 × 10^8^		
*B. longum* 428B	Adult stool sample	8.25 × 10^8^	3.27 × 10^8^	2.75 × 10^8^	9.01 × 10^7^		
*B. longum* 432B	Adult stool sample	1.39 × 10^9^	2.69 × 10^8^	5.08 × 10^8^	2.27 × 10^8^		
*B. longum* 433B	Adult stool sample	2.50 × 10^9^	5.00 × 10^8^	1.15 × 10^9^	4.95 × 10^8^		
*B. longum* 434B	Adult stool sample	1.92 × 10^9^	1.26 × 10^9^	5.50 × 10^8^	7.50 × 10^7^		
*B. longum* 442B	Adult stool sample	6.08 × 10^8^	5.20 × 10^7^	7.33 × 10^8^	2.25 × 10^8^	7.38 × 10^5^	7.61 × 10^4^
*B. longum* 447B	Adult stool sample	1.13 × 10^9^	4.06 × 10^8^	2.83 × 10^8^	1.46 × 10^8^		
*B. longum* 451B	Adult stool sample	1.31 × 10^9^	3.06 × 10^8^	5.08 × 10^8^	2.75 × 10^8^		
*B. longum* 499B	Colonoscopic sample	3.00 × 10^8^	1.15 × 10^8^	5.17 × 10^7^	3.82 × 10^6^		
*B. longum* 553B	Colonoscopic sample	2.33 × 10^9^	5.20 × 10^8^	5.92 × 10^8^	2.60 × 10^8^		
*B. longum* 606B	Colonoscopic sample	9.17 × 10^7^	9.46 × 10^7^	2.50 × 10^6^	0		
*B. longum* 633B	Adult stool sample	2.92 × 10^9^	1.88 × 10^9^	8.75 × 10^8^	2.54 × 10^8^		
*B. longum* 707B	Adult stool sample	9.25 × 10^8^	7.15 × 10^8^	3.75 × 10^8^	1.56 × 10^8^		
*B. longum* 71B	Adult stool sample	3.00 × 10^7^	8.66 × 10^6^	2.42 × 10^7^	6.29 × 10^6^		
*B. longum* 743B	Adult stool sample	1.58 × 10^8^	1.46 × 10^8^	4.92 × 10^7^	2.63 × 10^7^		
*B. longum* 861B	Adult stool sample	1.83 × 10^8^	5.77 × 10^7^	2.42 × 10^8^	7.64 × 10^7^	8.86 × 10^5^	1.54 × 10^5^
*B. longum* 908B	Colonoscopic sample	2.07 × 10^9^	4.16 × 10^8^	8.75 × 10^8^	6.61 × 10^7^		
*B. longum* subsp. *longum* LMG 13197	Colonoscopic sample	1.42 × 10^9^	7.22 × 10^8^	5.33 × 10^8^	4.47 × 10^8^		
*B. pseudocatenulatum* 202B	Colonoscopic sample	4.45 × 10^8^	3.82 × 10^8^	3.08 × 10^8^	1.28 × 10^8^		
*B. pseudocatenulatum* 263B	Adult stool sample	3.42 × 10^8^	2.45 × 10^8^	9.67 × 10^7^	2.89 × 10^7^		
*B. pseudocatenulatum* 289B	Adult stool sample	2.50 × 10^8^	4.33 × 10^7^	9.33 × 10^7^	6.29 × 10^6^		
*B. pseudocatenulatum* 318B	Colonoscopic sample	3.33 × 10^8^	8.04 × 10^7^	1.92 × 10^8^	1.44 × 10^7^		
*B. pseudocatenulatum* LMG 10505	Infant stool sample	1.67 × 10^9^	1.28 × 10^9^	6.67 × 10^7^	1.44 × 10^7^		
*B. pseudolongum* subsp. *globosum* 555B	Colonoscopic sample	1.33 × 10^8^	2.89 × 10^7^	3.75 × 10^8^	2.50 × 10^7^	5.52 × 10^5^	5.89 × 10^5^
*B. pseudolongum* subsp. *globosum* 685B	Adult stool sample	2.58 × 10^8^	1.44 × 10^8^	3.17 × 10^8^	1.42 × 10^8^	4.89 × 10^7^	3.86 × 10^6^
*B. pseudolongum* subsp. *globosum* 686B	Colonoscopic sample	3.08 × 10^8^	2.04 × 10^8^	1.83 × 10^8^	5.91 × 10^7^		
*B. pseudolongum* subsp. *globosum* LMG 11596	Bovine rumen	5.83 × 10^7^	3.82 × 10^7^	7.50 × 10^7^	3.54 × 10^7^	4.05 × 10^7^	1.17 × 10^7^
*B. stercoris* JCM 15918	Adult stool sample	7.92 × 10^7^	2.13 × 10^7^	1.25 × 10^7^	2.50 × 10^6^		

^a^ATCC: American Type Culture Collection, USA; LMG, Belgian Co-ordinated Collection of Microorganisms-Bacterial Collection, Belgium; DSM, German Collection of Microorganism and Cell Cultures, Germany; JCM Japan Collection of Microorganisms, Japan.

^b^EC: Enzyme Commission.

Values are expressed as the means ± standard errors from three experiments.

For those bifidobacterial strains showing an identical or higher growth level in MRS w/o glu + CG *vs*. MRS, growth performances were further evaluated using a different approach, i.e. viable cell count on MRS Agar (Table 1). Moreover, three bifidobacterial strains, i.e., *B. bifidum* LMG 11041, *B. breve* 31L and *B. dentium* LMG 11405, were selected as controls, since these strains showed reduced growth levels in MRS w/o glu + CG when compared to the cell numbers reached when grown on MRS.

Interestingly, all counts obtained through the plating method were lower than those obtained employing the Thoma cell counting chamber. This may not be surprising, since the Thoma chamber utilizes an indirect counting method and is unable to distinguish between living or devitalized cells, while the plating method exclusively evaluates viable cells. Notably, all strains grown using CG as the sole carbon source and then plated on MRS Agar were shown to be able to utilize the substrate tested. Bacterial counts ranged from 2.09 × 10^4^ CFU/mL for *B. breve* 31L cultures to 1.33 × 10^8^ CFU/mL for *B. breve* 2L cultures. Thus, *B. breve* 2L was the strain showing the highest growth performance when CG was provided as the sole carbon source. For this reason, *B. breve* 2L was chosen as a model bifidobacterial strain to dissect the genetic repertoire responsible for efficient CG metabolism.

### Identification of genes induced by CG in the genome of *B. breve* 2L

In order to identify the genes of *B. breve* 2L responsible for CG metabolism, we evaluated the transcriptomes of this strain when cultivated on CG by RNAseq analyses. The average transcriptome profile corresponding to the three replicates of *B. breve* 2L cultivated on MRS w/o glu + CG (0.5%(w/v) as the unique carbon source was compared with the average transcriptome profile of the three replicates of the positive control (*B. breve* 2L grown on complete MRS). Sequencing reads of *B. breve* 2L grown on CG as well as on glucose were mapped on the genome sequence of *B. breve* 2L. Subsequently, evaluation of RPKM (reads per kilobase per million mapped reads) values for each gene revealed high expression (ranging from 1464 to 24685 RPKM) of genes predicted to encode carbohydrate transporters (Table S1, annotation highlighted in red).

Notably, *B. breve* 2L grown in MRS w/o glu + CG (0.5% (w/v) showed increased transcription, ranging from 8.1 to 106-fold, of various genes, some of which were predicted to be involved in carbohydrate internalization (Table S2, annotation highlighted in red). Furthermore, significant transcriptional up-regulation, ranging from 8.2-fold to 14.6-fold, was observed for three genes encoding enzymes putatively involved in the hydrolysis of glycosidic linkages between hexose sugars (Table S2, annotation highlighted in blue).

A detailed scrutiny of the upregulated *B. breve* 2L genes upon its cultivation on MRS w/o glu + GC, revealed that the majority of these genes were organized in seven loci, which are predicted to encode carbohydrate transport systems (Table S3, annotation highlighted in red), or are coding for putative glycosyl hydrolases (Table S3, annotation highlighted in blue).

Altogether, these results indicate that the inclusion of CG in the growth medium as the unique carbohydrate source, modulates the expression of genes encoding enzymes toward the degradation and metabolism of CG.

Notably, CG was also observed to increase transcription of *tad* genes (Table S3, annotation highlighted in green) responsible for the synthesis and assembly of the Type IVb pilus locus, which has been shown in another *B. breve* strain to mediate the colonization and persistence of bifidobacterial cells in the mammalian gut^16–18^.

### Evaluation of the colonization of *B. breve* 2L in rats following GC treatment

**Figure 2:**
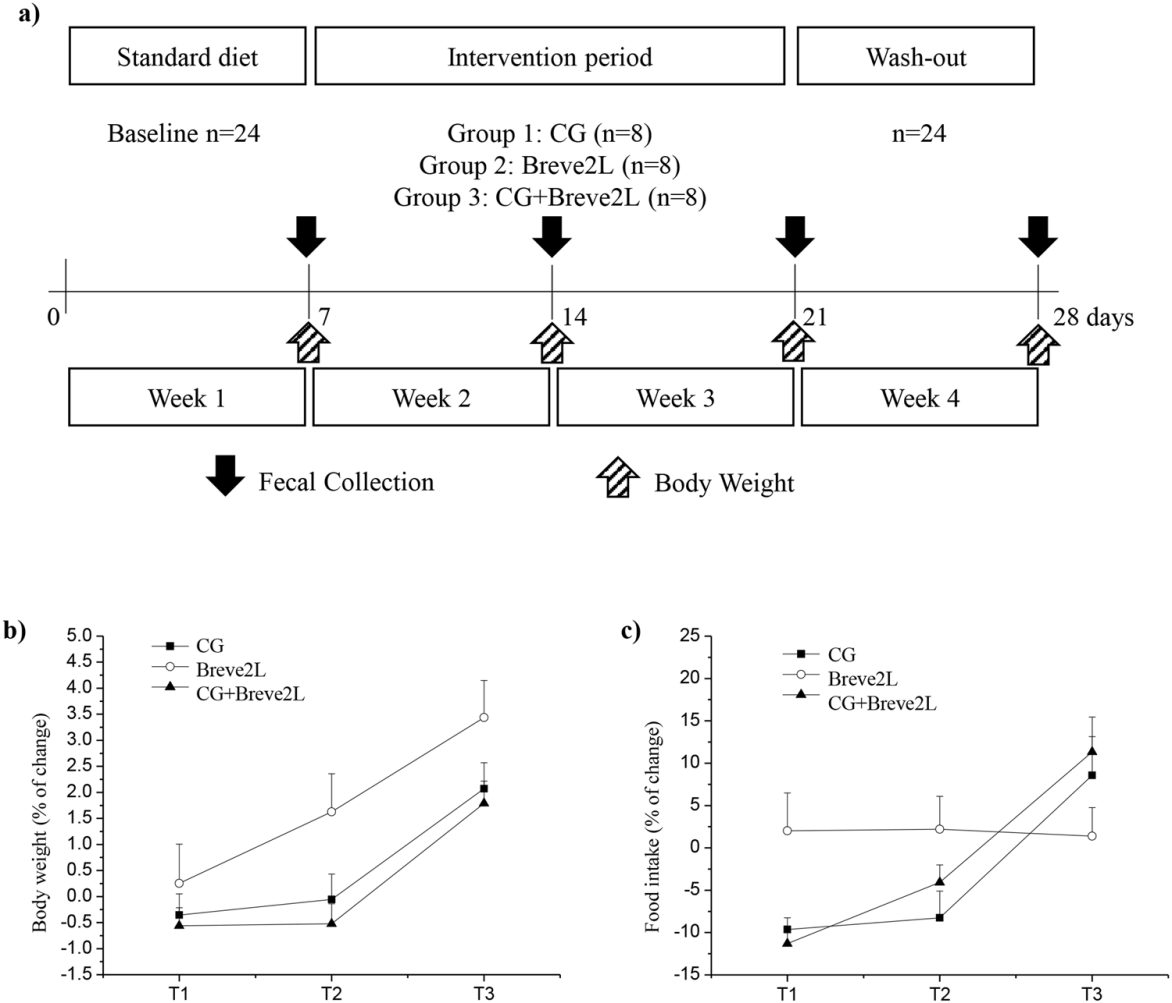
Design and results of the experimental procedure. Panel a shows the schedule of the experimental procedures. Panel b and c display the body weight and food intake percentage changes relative to the respective ‘T0’ values, during the experiment, respectively. Values are expressed as means ± SEM.

In order to evaluate if CG is able to modulate colonization of *B. breve* 2L in the mammalian gut, we performed an *in vivo* trial using Groningen rats (*Rattus norvegicus*). The trial consisted of three groups of animals, one receiving a daily inoculum of approximately 10^9^ CFU of *B. breve* 2L, i.e., Breve2L, a second group fed with standard diet supplemented with 10% CG, i.e., CG, and another one treated with the same amount of *B. breve* 2L cells plus 10% CG, i.e., CG + Breve2L (Fig. 2).

The putative physiological effect of CG on the Body Weight (BW) of the animals was recorded and compared to the Food Intake (FI) (Fig. 2b,c). Two-way ANOVA for repeated measures provided a significant effect of CG over time for BW changes (*p*-value < 0.05) and FI/BW (*p*-value < 0.01). BW increment was significantly higher in the Breve2L group as compared to the CG + Breve2L group at the end of the intervention period (T2) (*p*-value < 0.05). FI/BW ratio was significantly higher in the Breve2L group compared to either the CG (*p*-value < 0.05) or CG + Breve2L groups (*p*-value = 0.01), after the first week of the intervention period (T1). These data suggest that CG stimulates appetite while reducing/limiting body weight increment. In this context, characterization of the modulatory effects of CG toward the mammalian gut microbiota composition is pivotal to understand the physiological and metabolic aspects responsible for the reduction of body weight.

**Figure 3:**
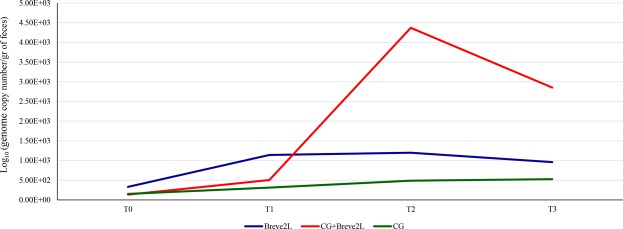
Quantitative PCR evaluations of the load of *B. breve* 2L in stool samples of rats. The graph reports the average abundance of *B. breve* 2L observed through qPCR in CG, Breve2L and CG + Breve2L groups at T0, T1, T2 and T3.

In order to assess the number of *B. breve* 2L in the fecal samples of the animals enrolled in this study, we applied a qPCR approach (Fig. 3). These analyses highlighted that rats of the CG groups did not show a statistically significant increment of *B. breve* 2L at the end of the experiment (T3) compared to the other time points. In contrast, a statistically significant increased load of *B. breve* 2L (p-value < 0.05) was observed at time points T1 (1.14E + 03 CFU/gr) and T2 (1.20E + 03 CFU/gr) compared to T0 (3.30E + 02 CFU/gr) in the Breve2L group, but there were no significant differences with respect to T3 (9.58E + 02 CFU/gr). These data suggest that the daily administration of *B. breve* 2L allows a transient colonization of the bacterial species as its concentration decreases after the washout week (T3). In this context, when rats were fed with both CG and *B. breve* 2L, the abundance of the *B. breve* strain was higher in T2 (4.37E + 03) and T3 (2.85E + 03) as compared to that observed for rats fed with only *B. breve* 2L, i.e. 1.20E + 03 CFU/gr at T2 and 9.58E + 02 CFU/gr at T3. Thus, supplementation of CG in the standard diet appeared to cause an increase in the abundance of *B. breve* 2L, thus enhancing gut colonization/persistence of this strain.

### Characterization of CG effects on the rat gut microbiota composition

**Figure 4:**
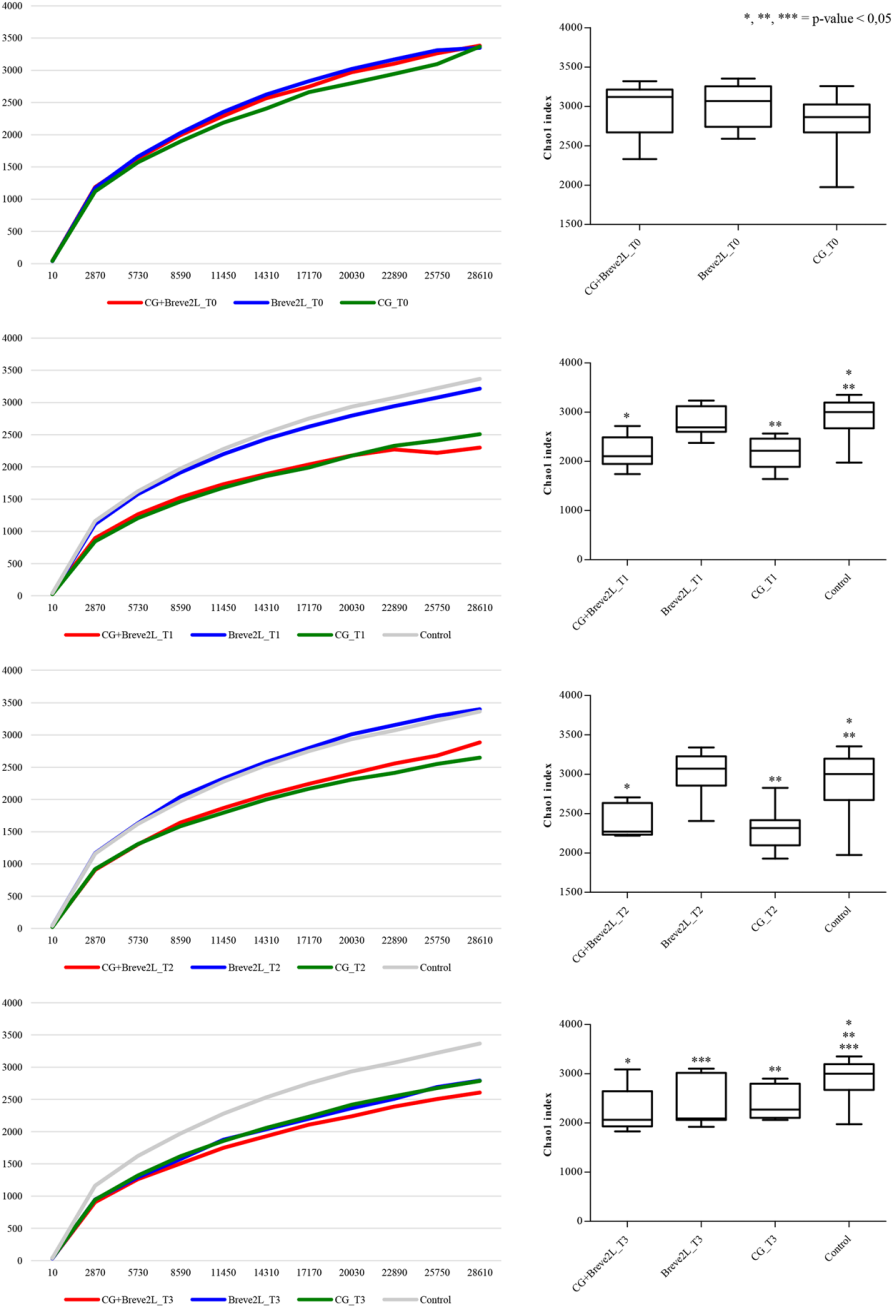
Alpha-diversity of CG, Breve2L and CG + Breve2L at T0, T1, T2 and T3 time points. Panels (a–d) report average alpha-diversity obtained using Chao1 index for T0, T1, T2 and T3 time points, respectively.

Evaluation of the gut microbiota composition of the animals enrolled in this study was performed by 16 S gene rRNA microbial profiling analyses on fecal samples collected during the trial. Analysis of sequencing data produced a total of 4,581,503 quality-filtered reads with an average of 47,724 reads per sample (Table S4). Evaluation of alpha-diversity, i.e. the biodiversity, of the collected rat fecal samples was performed through analysis of rarefaction curves constructed with 10 sub-sampling of the whole sequenced datasets. Notably, alpha-diversity data revealed, as expected, that at T0 all animal groups possessed similar microbiota diversity (t-test p-values > 0.05) (Fig. 3). T0 samples were used as “Control” non-treated group for analysis of T1, T2 and T3 time points. Intriguingly, analysis of data collected for T1 and T2 revealed that CG supplementation appeared to reduce the microbiota diversity, as observed for CG and CG + Breve2L compared to T0 and Breve2L sample groups (Fig. 4). These observations were confirmed by statistical analysis by means of t-test at 20,000 reads of CG and CG + Breve2L with respect to the same animal at T0, resulting in p-values < 0.05 (Fig. 4). Furthermore, data collected at T3 showed that cessation of CG supplementation does not reverse the biodiversity of the CG and CG + Breve2L groups to pre-treatment levels. These observations indicate that CG modulates the microbiota through selection of specific bacterial taxa, with subsequent reduction of the overall gut microbiota diversity, which persists (at least for the period tested) following termination of CG supplementation. Similar data were previously observed for other prebiotic compounds^7^.

**Figure 5:**
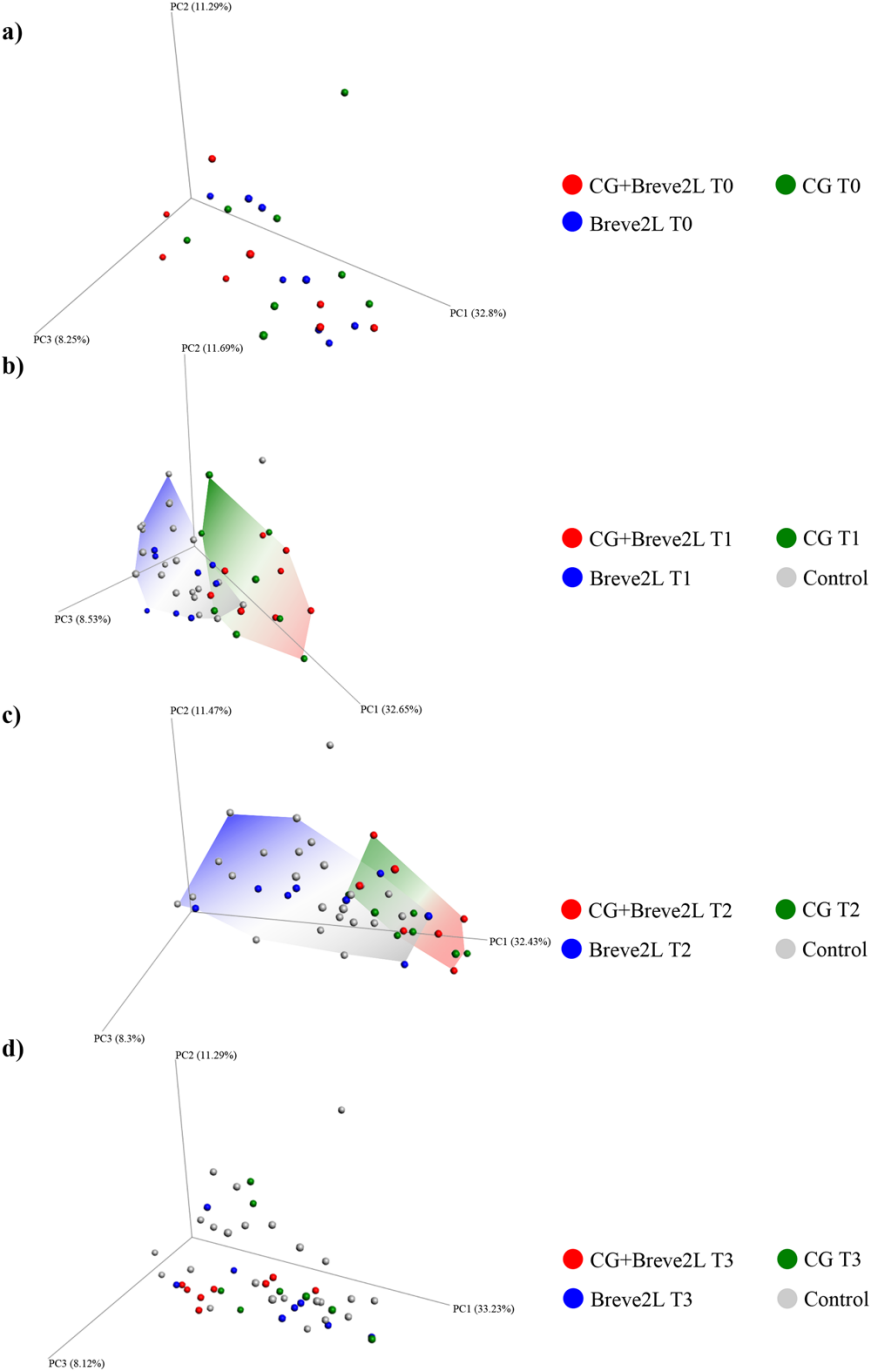
Beta-diversity of CG, Breve2L and CG + Breve2L at T0, T1, T2 and T3 time points. Panels a,b,c and d report three dimensional PCoA obtained using Weighted Unifrac index for T0, T1, T2 and T3 time points, respectively.

Beta-diversity analysis did not reveal any statistically significant differences between sample groups at T0 (PERMANOVA p-value > 0.05) and confirmed the modulatory effect exerted by CG at T1 and T2 (Fig. 5). In fact, PERMANOVA statistical analysis between CG and CG + Breve2L groups at T1 and T2 with respect to T0 datasets resulted in all cases in p-values < 0.05 (Fig. 5). Notably, cessation of CG supplementation causes the CG and CG + Breve2L sample groups to cluster together with Breve2L and Control datasets at T3 (Fig. 5). Thus, beta-diversity data confirms the modulatory effect exerted by CG on the animals’ gut microbiota composition.

**Figure 6:**
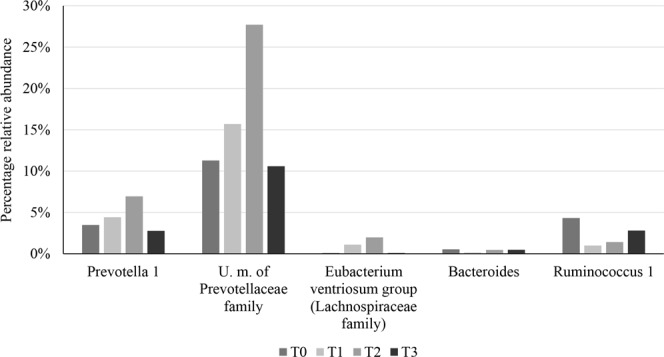
Bacterial genera whose abundance is highly modulated by CG. The bar plot reports the average relative abundance observed in CG samples group of relevant genera modulated by CG supplementation.

Taxonomic composition of all samples was reconstructed at phylum and genus levels in order to detail the impact of CG on the gut microbiota (Supplementary File 1). A t-test between the relative abundance of each profiled bacterial genus observed in the CG, Breve2L and CG + Breve2L groups was performed and compared between the various time points. Furthermore, statistically significant taxa observed for each comparison were mapped in order to precisely identify those bacterial genera whose relative abundance was increased or decreased upon CG and/or *B. breve* 2 L supplementation (Fig. 6). Intriguingly, 10 and 12 taxa showed increased (when compared to T0) relative abundance during CG supplementation at T1 and T2, respectively, followed by a decrease in abundance at T3, i.e. after cessation of CG supplementation (Fig. 6). In this context, it is worth mentioning that *Prevotella 1*, U. m. of *Prevotellaceae* family and *Eubacterium ventriosum* group (*Lachnospiraceae* family) increased their abundance by 100%, 145.4% and 2072.5% in T2 when compared to T0 (Fig. 6) (Supplementary File 1). In contrast, 42 and 38 taxa showed decreased relative abundance at T1 and T2, respectively, followed by an increase in abundance at T3 (Fig. 6) (Supplementary File 1). The observed decreased relative abundance of *Bacteroides* as well as *Ruminococcus* (−15.5% and −67.2% in T2 when compared to T0) and the simultaneous increase in relative abundance of *Prevotella* suggest that CG promotes a shift of the gut microbiota towards a Type 2 enterotype, i.e. *Prevotella*-driven enterotype^19^.

Intriguingly, CG was observed to induce increased relative abundance of *Akkermansia*, a taxon with health-promoting activities^20^ and reduced relative abundance of *Peptoclostridium*, a genus encompassing opportunistic pathogens^21^.

Analysis of the impact of *B. breve* 2L and CG administration on the animal microbiota could not be accurately performed through 16S rRNA gene microbial profiling due to the low relative abundance of bifidobacteria in the assessed fecal samples. For this reason, we performed a detailed cataloguing of bifidobacterial communities through the use of a previously published bifidobacterial ITS profiling approach^22,23^, allowing a detailed cataloguing of the bifidobacterial population down to the (sub)species level.

### Cataloguing of the rat fecal bifidobacterial communities residing

In order to detail the impact of CG supplementation on the bifidobacterial population colonizing the rat gut, we performed bifidobacterial ITS profiling of DNA extracted from all fecal samples collected in this study. Sequencing produced a total of 316,080 reads, with an average of 3,293 reads per sample (Table S5).

**Figure 7:**
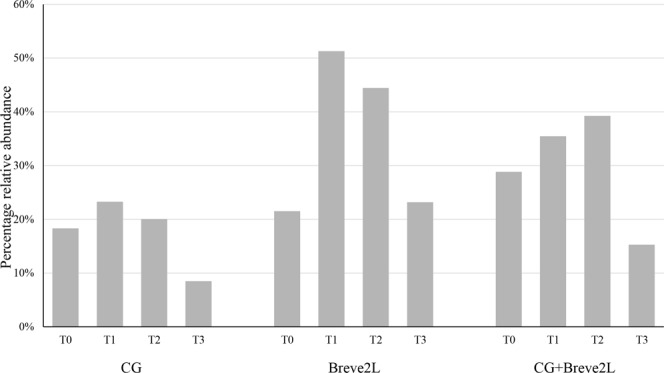
*B. breve* 2L relative abundance respect to the total bifidobacterial population. The bar plot reports the average relative abundance of *B. breve* 2L observed in CG, Breve2L and CG + Breve2L at T0, T1, T2 and T3.

In order to detail the modulatory effects of CG, we performed inspection of (sub)species-level profiles and statistical analysis through t-test of CG, Breve2L as well as CG + Breve2L groups of samples at T1, T2 and T3 compared to T0 (Supplementary File 2). As expected, an increase in the relative abundance of *B. breve* was observed in the Breve2L and CG + Breve2L groups at T1 and T2, which was followed by a relative decrease in *B. breve* 2L abundance at T3 (Fig. 7). Moreover, data of samples constituting the CG group revealed that CG supplementation induces a statistically significant increase in relative abundance (p-value < 0.05) of eight bifidobacterial species at T2 (Supplementary File 2). Furthermore, when CG was supplemented in combination with *B. breve* 2L, this bifidobacterial species (and most likely the *B. breve* 2L strain) was shown to reach a higher relative abundance as compared to those animals only receiving *B. breve* 2L (Fig. 7), as also indicated by qPCR results (Fig. 2). These results indicate that CG exerts a species-specific modulation of the bifidobacterial population harbored by the rat gut.

## Conclusion

Evaluation of the potential prebiotic features of chitin-glucan toward bifidobacteria under *in vitro* conditions highlighted the ability of 100 bifidobacterial strains to use this substrate as its sole carbon source. The bifidobacterial species that was most effective in utilizing CG for growth was shown to be *B. breve*. Notably, these results reflect the ability of this typical infant gut colonizer to utilize host-produced glycans, whose molecular structure partially resembles that of CG.

RnaSeq transcriptomics data was performed for the *B. breve* strain showing the best CG utilization capabilities, i.e. *B. breve* 2L, pointing out a modulation of the *B. breve* 2L genes involved in the transport and metabolism of hexose sugars.

The *in vivo* trials in a rat model clearly supported the notion that CG exploits a clear bifidogenic effect on bifidobacteria and in particular of small number of bifidobacterial species such as *B. breve*. These findings reinforce the potential of GC in modulating and shaping the bifidobacterial communities especially in ecological conditions where bifidobacteria are depleted (e.g., very often associated with metabolic disorders or gut diseases). In this context, the capability of bifidobacteria to use the CG as carbon source and the subsequent degradation of this bio-polymer in simpler derivatives, i.e. chitooligosaccharides (COS), may support the notion that CG act as a prebiotic. Indeed, it has been largely demonstrated that COS are able to inhibit the growth of pathogenic bacteria^24,25^. Moreover, CG consumption was shown to reduce body weight increment in rats, thus pointing at CG as an interesting novel prebiotic for prevention and treatment of obesity.

Such *in vivo* data should be further confirmed by clinical trials performed in human beings consuming CG or CG-based products (e.g., symbiotic products) in those categories of individuals where bifidobacterial abundance is naturally low (e.g., in the elderly) or is depleted as a consequence of metabolic disorder (e.g., constipation) or diseases (auto-immune diseases) as well as antibiotic therapy.

It is also arguable that cross-feeding interactions might be established by the different members of the bifidobacterial communities as well as with the different members of the human gut microbiota for the complete metabolism of CG. This was previously shown in co-participated trophic interactions^15,26^ where a partner partially metabolizes GC in favor to another microorganism that is genetically incapable to utilize this substrate. Thus, cross-feeding might represent another valuable way exploited by CG to induce a more general prebiotic effect in the human gut.

## Methods

### Strains and culture conditions

*Bifidobacterium* strains used in this study are listed in Table 1. Strains were routinely grown anaerobically in De Man, Rogosa, Sharpe (MRS) medium (Scharlau) containing 2% glucose (w/v), which was supplemented with 0.05% L-cysteine-HCl and incubated at 37 °C for 24 h. Anaerobic conditions were achieved by the use of an anaerobic cabinet (Ruskin), in which the atmosphere consisted of 17% CO_2_, 80% N_2_, and 2.99% H_2_.

### Ethical statement

All experimental procedures and protocols involving animals were approved by the Veterinarian Animal Care and Use Committee of Parma University (approved protocol 370/2018-PR) and conducted in compliance with the European Community Council Directives dated 22 September 2010 (2010/63/UE).

### Chitin-glucan growth assay

CG (KitoZyme, Belgium) was added to MRS without glucose (MRS w/o glu) at a final concentration of 0.5% (w/v), as previously assessed through growth experiments involving various bifidobacteria on different carbon sources^14^. This growth medium was termed MRS w/o glu + CG and subjected to sterilization employing a modified Tyndallization approach consisting of two thermal cycles at 80 °C for 30 minutes each alternated with cooling in ice.

For all growth tests, cells were recovered from an overnight culture and turbidity was measured at 600 nm, using a biophotometer (Eppendorf). A growth tube containing 6 mL of MRS w/o glu + CG was inoculated with active viable bacterial cells diluted to an OD_600nm_ of ~1.0, obtaining a final inoculum with an OD_600nm_ of ~0.1. Cultures were grown in biologically independent triplicates and the resulting growth data sets were expressed as the means from these replicates. Moreover, positive (MRS) and negative (MRS w/o glu) growth controls were performed. Cultures were incubated under anaerobic conditions at 37 °C for 24 h. Cell growth was monitored using a Thoma cell counting chamber (Herka).

For some bifidobacterial strains, cell growth was also monitored by viable cell count in MRS Agar. For this purpose, strains were inoculated as mentioned above in MRS w/o glu + CG. Then, 1 mL of each grown strain was serially diluted in PBS (Phosphate buffered saline) and plated on MRS agar. Plates were incubated under anaerobic conditions at 37 °C for 48 h. Bacterial growth was assessed by colony counting.

### RNA-Seq transcriptomic analysis and identification of genes induced by CG

Bacterial cells were recovered from an overnight culture and turbidity was measured at 600 nm, using a biophotometer (Eppendorf). A growth tube containing 40 mL of complete MRS (reference condition) as well as 40 ml of MRS w/o glu CG was inoculated with viable bacterial cells diluted to an OD_600nm_ of ~1.0, obtaining a final inoculum with an OD_600nm_ of ~0.1. All growth conditions were performed by incubation in anaerobic cabinet at 37 °C. Following inoculation, growth was monitored and when an OD_600nm_ value between 0.6 and 0.8 (exponential phase) cells were centrifuged at 6000 rpm for 5 min. Finally, prior to RNA extraction cells were frozen at −80 °C. Growth assays were carried out in triplicate. Total RNA was isolated from *B. breve* 2L cultures grown in MRS medium (Scharlau, Italy) as well as MRS w/o glu + GC. The obtained cell pellet was resuspended in 1 ml of QIAZOL (Qiagen, United Kingdom) and placed in a tube containing 0.8 g of glass beads (diameter, 106 μm; Sigma). Cells were lysed by shaking the mix on a Precellys 24 homogenizer (Bertin instruments, France). The mixture was then centrifuged at 13,000 rpm for 15 min, and the RNA-containing upper phase was recovered. RNA was further purified using RNeasy mini kit (Qiagen, UK) as reported in the manufacturer’s instructions.

RNA quality was checked by a Tape station 2200 (Agilent Technologies, USA) analysis. RNA concentration and purity were evaluated by Picodrop microliter spectrophotometer (Picodrop, UK).

For RNA sequencing, 2 μg of total RNA was treated to remove ribosomal RNA by the Ribo-Zero Magnetic Kit (Illumina), followed by purification of the rRNA-depleted sample by ethanol precipitation. RNA was processed according to the manufacturer’s protocol. The efficacy of rRNA depletion was checked by a Tape station 2200 (Agilent Technologies). Then, 500 ng of rRNA-depleted RNAs was fragmented using Bioruptor NGS ultrasonicator (Diagenode, USA) followed by size evaluation using Tape station 2200 (Agilent Technologies). A whole transcriptome library was constructed using the TruSeq Stranded RNA LT Kit (Illumina). Samples were loaded into a Flow cell V2 75 cycles (Illumina) as reported by the technical support guide.

Sequencing reads were mapped with Burrows-Wheeler Aligner (BWA)^27^ to the genomic sequence of *B. breve* 2L (NCBI accession number AWUG00000000.1). Reads Per Kilobase per Million mapped reads (RPKM) of each gene were assessed using Artemis software^28^.

### Animal housing

Experiments involved 5-month-old male wild-type Groningen rats (*Rattus norvegicus*). After weaning, rats were housed in same sex sibling groups in rooms under humidity (50 ± 10%) and temperature-controlled conditions (22 ± 2 °C), a 12-h light-dark cycle (lights on at 7 a.m.), and with food and water available *ad libitum*.

### Experimental design of the *in vivo* trials

From the initiation of the experiments, rats were housed individually in polymethyl methacrylate (Plexiglas®) cages (39 cm × 23 cm × 15 cm). The first week represented an acclimatization period, during which rats continued to consume a standard chow diet supplemented with an oral administration of 500 µl of sucrose solution (2%) in order to adapt the rats to drink from the syringe. Maintaining their habits, rats could represent the negative control of themselves, acting as the baseline for subsequent microbiota analyses^29^. For the following two weeks (14 days), rats (n = 24) were randomized to 3 groups: a first group fed with standard diet supplemented with CG [90% standard diet (w/w) + 10% KiOnutrime-CG from KitoZyme, Belgium; CG group], a second group fed with a standard diet and an oral treatment with *B. breve* 2L (Breve2L group) and a third group fed with standard diet supplemented with CG (same composition of the CG group) and oral treatment with *B. breve* 2L (CG + Breve2L group) (Table 2). The CG group rats during the intervention period were orally inoculated with 500 µl of sucrose solution (2%) in order to maintain the same condition for the three groups except for the experimental variables. Finally, during the last week of the wash-out period, all animals returned to the standard chow diet.

**Table 2:** Fecal samples from rats collected at the different time points.

Samples	Type of intervention	Time points
WT1A – WT8A	CG group	T_0_
WT1B – WT8B		T_1_
WT1C – WT8C		T_2_
WT1D – WT8D		T_3_
WT9A – WT16A	Breve2L group	T_0_
WT9B – WT16B		T_1_
WT9C – WT16C		T_2_
WT9D – WT16D		T_3_
WT17A – WT24A	CG + Breve2L group	T_0_
WT17B – WT24B		T_1_
WT17C – WT24C		T_2_
WT17D – WT24D		T_3_

Standard diet consisted of 54.61% nitrogen-free extract (mainly represented by starch and hemicellulose), 5.54% fibers, 19.42% protein, 11.09% water, 2.58% lipids, and 6.76% ash (non-organic mineral matter) (3.9 kcal/g; 4RF21, Mucedola, Italy). The percentage of supplementation of the substrate CG to standard chow diet was fixed at 10% (w/w) as previously illustrated^6^.

Food intake (FI) and body weight (BW) were measured daily and weekly, respectively, and the BW changes and FI were calculated as previously described^7^.

### Evaluation of *Bifidobacterium breve* cell numbers by qPCR

Quantitative PCR (qPCR) was assessed using the species-specific primers BiBre1 (5′-CCGGATGCTCCATCACAC-3′) and BiBre2 (5′-ACAAAGTGCCTTGCTCCCT-3′). qPCR was performed using GoTaq qPCR Master Mix (Promega, USA) on a CFX96 system (BioRad, CA, USA) following previously described protocols^30^. PCR products were detected with SYBR green fluorescent dye and amplified according to the following protocol: one cycle of 95 °C for 2 min, followed by 40 cycles of 95 °C for 3 s and 56 °C for 30 s. The melting curve was 65 °C to 95 °C with increments of 0.5 °C/s. In each run, negative controls (no DNA) were included. A standard curve was built using the CFX96 software (BioRad).

### Fecal bacterial DNA extraction and 16S rRNA/ITS microbial profiling

Fecal samples were subjected to DNA extraction using the QIAmp DNA Stool Mini Kit following the manufacturer’s instructions (Qiagen). Partial 16S rRNA gene sequences were amplified from extracted DNA using primer pair Probio_Uni/Probio_Rev, which targets the V3 region of the 16S rRNA gene sequence^31^. Partial ITS sequences were amplified from extracted DNA using the primer pair Probio-bif_Uni/Probio-bif_Rev, which targets the spacer region between the 16S rRNA and the 23S rRNA genes within the ribosomal RNA (rRNA) locus^22^. Illumina adapter overhang nucleotide sequences were added to the partial 16S rRNA gene-specific amplicons and to the generated ITS amplicons of 200 bp, which were further processed using the 16S Metagenomic Sequencing Library Preparation Protocol (Part No. 15044223 Rev. B—Illumina). Amplifications were carried out using a Verity Thermocycler (Applied Biosystems). The integrity of the PCR amplicons was analyzed by electrophoresis on a 2200 TapeStation Instrument (Agilent Technologies, USA). DNA products obtained following PCR-mediated amplification of the 16S rRNA gene sequences were purified by a magnetic purification step involving the Agencourt AMPure XP DNA purification beads (Beckman Coulter Genomics GmbH, Bernried, Germany) in order to remove primer dimers. DNA concentration of the amplified sequence library was determined by a fluorimetric Qubit quantification system (Life Technologies, USA). Amplicons were diluted to a concentration of 4 nM, and 5 µL quantities of each diluted DNA amplicon sample were mixed to prepare the pooled final library. 16S rRNA gene and ITS sequencing were performed using an Illumina MiSeq sequencer with MiSeq Reagent Kit v3 chemicals.

### Metagenomics analyses

Following sequencing, the.fastq files were processed using a custom script based on the QIIME software suite^32^. Paired-end read pairs were assembled to reconstruct the complete Probio_Uni/ Probio_Rev and Probio-bif_Uni/Probio-bif_Rev amplicons. Quality control retained sequences with a length between 140 and 400 bp and mean sequence quality score >20 while sequences with homopolymers >7 bp and mismatched primers were omitted. In order to calculate downstream diversity measures (alpha and beta diversity indices, Unifrac analysis), 16S rRNA Operational Taxonomic Units (OTUs) were defined at ≥99% sequence homology using uclust^33^ and OTUs with less than 10 sequences were filtered. ITS Operational Taxonomic Units (OTUs) were defined at 100% sequence homology using uclust^33^. All reads were classified to the lowest possible taxonomic rank using QIIME^32^ and a reference dataset from the SILVA database^34^ for 16S rRNA data or an updated version of the bifidobacterial ITS database^22^ for ITS data. Biodiversity of the samples (alpha-diversity) were calculated with Chao1 and Shannon indexes. Similarities between samples (beta-diversity) were calculated by unweighted uniFrac^35^. The range of similarities is calculated between the values 0 and 1. PCoA representations of beta-diversity were performed using QIIME^32^.

### Statistical analysis

For *in vitro* trials, statistical significance between means was analyzed using the Student’s t-test. Values are expressed as the means ± standard errors from three experiments. Statistical calculations were performed using the software program GraphPad Prism 5 (La Jolla, CA, USA).

For *in vivo* experiments, two-way ANOVA for repeated measures with ‘group’ as between-subject factor (three levels: CG group, Breve2L group and CG + Bbreve2L group) was performed for: (i) BW changes, with ‘time’ as within-subject factor (three levels: T1, T2, T3); (ii) FI-to-BW ratio, with ‘time’ as within-subject factor (four levels: week1, week2, week3 and week4). Follow up analysis was performed using Student’s t-test, with a Bonferroni correction for multiple comparisons.

Furthermore, the difference between the rarefaction curves and relative abundance of taxa, as well as the differential abundance of bacterial genera were statistically analysed by t-test. All statistical analyses were performed through SPSS software (www.ibm.com/software/it/analytics/spss/).

### Data Deposition

Raw sequences of 16S rRNA gene profiling and bifidobacterial ITS profiling as well as RNAseq data are accessible through SRA study accession numbers SRP164382 and SRP164394.

## Supplementary information

Supplementary Files

Supplementary Tables
