# Supplementary material for: Ability of bifidobacteria to metabolize chitin-glucan and its impact on the gut microbiota
Source: Sci Rep. 2019 Apr 8;9:5755. doi: 10.1038/s41598-019-42257-z (PMC6453949; doi:10.1038/s41598-019-42257-z)
Supplement: Supplementary file 1 — Supplementary Files [file 41598_2019_42257_MOESM1_ESM.pdf]

Ability of bifidobacteria to metabolize chitin-glucan and its impact on the gut microbiota

Key words: Bifidobacterium, prebiotics, gut microbiota, microbe-microbe interactions

Running title: Bifidobacteria and chitin glucan

Giulia Alessandri[1#], Christian Milani[2#], Sabrina Duranti[2#], Leonardo Mancabelli[2], Thibaut Ranjanoro[3], Salvatore Modica[3], Luca Carnevali[4], Rosario Statello[4], Francesca Bottacini[5], Francesca Turroni[2], Maria Cristina Ossiprandi[1], Andrea Sgoifo[4], Dowue van Sinderen[5] and Marco Ventura[1]

[#] These authors contributed equally

Department of Veterinary Medical Science, University of Parma, Parma, Italy[1]; Laboratory of Probiogenomics, Department of Chemistry, Life Sciences, and Environmental Sustainability, University of Parma, Parma, Italy[2]; KitoZyme, Belgium[3]; Stress Physiology Laboratory, Department of Chemistry, Life Sciences and Environmental Sustainability, University of Parma, Parma, Italy[4]; APC Microbiome Institute and School of Microbiology, Bioscience Institute, National University of Ireland, Cork, Ireland[5]

Correspondence. Mailing address for Marco Ventura; Laboratory of Probiogenomics, Department of Chemistry, Life Sciences, and Environmental Sustainability, University of Parma, Parco Area delle Scienze 11a, 43124 Parma, Italy. Phone: ++39-521-905666. Fax: ++39-521-905604. E-mail: marco.ventura@unipr.it

Supplementary File 1

Absolute variation (%) at phylum level

		CG			Breve2L			CG+Breve2L		
		**T1-T0**	**T2-T0**	**T3-T0**	**T1-T0**	**T2-T0**	**T3-T0**	**T1-T0**	**T2-T0**	**T3-T0**
D_0__Archaea;D_1__Euryarchaeota	Euryarchaeota	0.00%	0.00%	0.00%	[redacted]	0.00%	[redacted]	0.00%	0.00%	0.00%
D_0__Bacteria;D_1__Actinobacteria	Actinobacteria	0.02%	-0.07%	0.11%	0.02%	0.09%	0.06%	0.00%	-0.08%	0.02%
D_0__Bacteria;D_1__Bacteroidetes	Bacteroidetes	10.26%	16.94%	1.52%	0.72%	0.29%	-0.25%	13.33%	22.01%	0.59%
D_0__Bacteria;D_1__Chloroflexi	Chloroflexi	-0.01%	-0.01%	-0.01%	[redacted]	[redacted]	[redacted]	[redacted]	[redacted]	[redacted]
D_0__Bacteria;D_1__Cyanobacteria	Cyanobacteria	0.00%	0.08%	0.13%	0.01%	0.18%	0.23%	0.10%	0.28%	0.08%
D_0__Bacteria;D_1__Deferribacteres	Deferribacteres	0.00%	0.00%	0.00%	0.00%	0.00%	0.00%	0.00%	0.00%	0.00%
D_0__Bacteria;D_1__Firmicutes	Firmicutes	-8.27%	-14.66%	-2.75%	0.39%	-0.52%	1.56%	-9.05%	-18.45%	1.03%
D_0__Bacteria;D_1__Fusobacteria	Fusobacteria	-0.02%	-0.02%	0.01%	-0.01%	0.00%	0.02%	0.01%	0.01%	0.02%
D_0__Bacteria;D_1__Proteobacteria	Proteobacteria	-0.97%	-0.23%	-0.21%	-0.10%	-0.14%	0.02%	-1.87%	-1.38%	0.29%
D_0__Bacteria;D_1__Saccharibacteria	Saccharibacteria	0.03%	-1.35%	0.65%	-0.37%	-0.02%	-0.77%	-1.67%	-2.53%	-1.42%
D_0__Bacteria;D_1__Spirochaetae	Spirochaetae	0.01%	0.30%	0.08%	0.00%	0.11%	0.09%	-0.05%	1.00%	0.19%
D_0__Bacteria;D_1__Tenericutes	Tenericutes	-1.00%	-1.11%	0.46%	-0.59%	-0.16%	-0.86%	-0.67%	-0.81%	-0.61%
D_0__Bacteria;D_1__Verrucomicrobia	Verrucomicrobia	-0.01%	0.00%	0.00%	0.00%	0.00%	-0.01%	0.02%	0.00%	0.01%
D_0__Bacteria;Other	U. m. of Bacteria kingdom	-0.03%	0.11%	-0.01%	-0.05%	0.12%	-0.07%	-0.11%	-0.04%	-0.14%
Unclassified;Other	Others	-0.01%	0.02%	0.01%	-0.02%	0.04%	-0.02%	-0.03%	-0.02%	-0.05%

Relative variation (%) at phylum level

		CG			Breve2L			CG+Breve2L		
		**T1-T0**	**T2-T0**	**T3-T0**	**T1-T0**	**T2-T0**	**T3-T0**	**T1-T0**	**T2-T0**	**T3-T0**
D_0__Archaea;D_1__Euryarchaeota	Euryarchaeota	-89.55%	Absent in T2	Absent in T3	Absent in both	Absent in T0	Absent in both	-33.18%	-42.40%	-91.48%
D_0__Bacteria;D_1__Actinobacteria	Actinobacteria	16.33%	-49.27%	83.57%	21.53%	108.81%	68.80%	0.58%	-51.39%	14.34%
D_0__Bacteria;D_1__Bacteroidetes	Bacteroidetes	19.59%	32.35%	2.91%	1.30%	0.53%	-0.45%	28.13%	46.45%	1.25%
D_0__Bacteria;D_1__Chloroflexi	Chloroflexi	Absent in T1	Absent in T2	Absent in T3	Absent in both	Absent in both	Absent in both	Absent in both	Absent in both	Absent in both
D_0__Bacteria;D_1__Cyanobacteria	Cyanobacteria	-4.37%	161.10%	249.82%	22.37%	700.72%	888.90%	278.40%	820.99%	217.85%
D_0__Bacteria;D_1__Deferribacteres	Deferribacteres	-64.06%	215.77%	249.95%	-66.63%	-34.34%	82.35%	-77.10%	-1.56%	9.16%

D_0__Bacteria;D_1__Firmicutes	Firmicutes	-19.85%	-35.19%	-6.60%	1.03%	-1.37%	4.12%	-20.22%	-41.23%	2.31%
D_0__Bacteria;D_1__Fusobacteria	Fusobacteria	-78.35%	-81.55%	42.75%	-41.24%	-11.45%	57.48%	134.51%	90.26%	260.24%
D_0__Bacteria;D_1__Proteobacteria	Proteobacteria	-37.18%	-8.76%	-8.05%	-2.95%	-4.23%	0.71%	-55.83%	-41.03%	8.53%
D_0__Bacteria;D_1__Saccharibacteria	Saccharibacteria	1.78%	-82.58%	39.85%	-17.16%	-0.94%	-36.20%	-57.05%	-86.64%	-48.67%
D_0__Bacteria;D_1__Spirochaetae	Spirochaetae	29.17%	705.53%	180.45%	3.13%	557.56%	439.06%	-47.89%	1020.66%	197.34%
D_0__Bacteria;D_1__Tenericutes	Tenericutes	-75.38%	-84.22%	34.99%	-39.89%	-11.08%	-58.35%	-64.12%	-77.47%	-58.40%
D_0__Bacteria;D_1__Verrucomicrobia	Verrucomicrobia	-45.61%	-23.33%	19.81%	-2.34%	18.00%	-37.14%	364.62%	37.85%	224.55%
D_0__Bacteria;Other	U. m. of Bacteria kingdom	-30.99%	113.38%	-10.26%	-51.54%	123.40%	-75.60%	-62.04%	-22.61%	-82.36%
Unclassified;Other	Others	-66.65%	118.94%	55.32%	-81.52%	183.01%	-92.25%	-56.82%	-32.54%	-87.31%

Trend (effect of treatment respect to T0 controls)

		CG			Breve2L			CG+Breve2L		
		T0-T1	T0-T2	T0-T3	T0-T1	T0-T2	T0-T3	T0-T1	T0-T2	T0-T3
D_0__Archaea;D_1__Euryarchaeota	Euryarchaeota	-	-	-		+		-	-	-
D_0__Bacteria;D_1__Actinobacteria	Actinobacteria	+	-	+	+	+	+	+	-	+
D_0__Bacteria;D_1__Bacteroidetes	Bacteroidetes	+	+	+	+	+	-	+	+	+
D_0__Bacteria;D_1__Chloroflexi	Chloroflexi	-	-	-						
D_0__Bacteria;D_1__Cyanobacteria	Cyanobacteria	-	+	+	+	+	+	+	+	+
D_0__Bacteria;D_1__Deferribacteres	Deferribacteres	-	+	+	-	-	+	-	-	+
D_0__Bacteria;D_1__Firmicutes	Firmicutes	-	-	-	+	-	+	-	-	+
D_0__Bacteria;D_1__Fusobacteria	Fusobacteria	-	-	+	-	-	+	+	+	+
D_0__Bacteria;D_1__Proteobacteria	Proteobacteria	-	-	-	-	-	+	-	-	+
D_0__Bacteria;D_1__Saccharibacteria	Saccharibacteria	+	-	+	-	-	-	-	-	-
D_0__Bacteria;D_1__Spirochaetae	Spirochaetae	+	+	+	+	+	+	-	+	+
D_0__Bacteria;D_1__Tenericutes	Tenericutes	-	-	+	-	-	-	-	-	-
D_0__Bacteria;D_1__Verrucomicrobia	Verrucomicrobia	-	-	+	-	+	-	+	+	+
D_0__Bacteria;Other	U. m. of Bacteria kingdom	-	+	-	-	+	-	-	-	-
Unclassified;Other	Others	-	+	+	-	+	-	-	-	-

Taxonomy	Absolute variation (%) at genus level								
	CG			Breve2L			CG+Breve2L		
	T1-T0	T2-T0	T3-T0	T1-T0	T2-T0	T3-T0	T1-T0	T2-T0	T3-T0
U. m. of Euryarchaeota phylum	0.00%	0.00%	0.00%	[redacted]	0.00%	[redacted]	0.00%	0.00%	0.00%
Actinomyces	0.00%	0.00%	0.01%	0.00%	0.00%	0.00%	-0.01%	-0.01%	-0.01%
Bifidobacterium	0.02%	-0.01%	0.07%	0.03%	0.07%	0.04%	0.03%	-0.01%	0.04%
Gardnerella	0.00%	0.00%	0.00%	[redacted]			0.00%	0.00%	0.00%
Adlercreutzia	0.00%	0.00%	0.00%	0.00%	0.00%	0.00%	0.00%	0.00%	0.00%
Atopobium	0.00%	0.00%	0.00%	0.00%	0.00%	0.00%	0.00%	0.00%	0.00%
Collinsella	0.00%	0.00%	0.02%	0.00%	0.01%	0.02%	0.00%	0.00%	0.02%
Coriobacteriaceae UCG-002	0.01%	0.01%	0.00%	0.00%	0.00%	0.00%	0.00%	0.01%	0.00%
Enterorhabdus	-0.01%	-0.01%	0.00%	0.00%	0.01%	0.01%	-0.02%	-0.02%	-0.01%
Gordonibacter	0.00%	0.00%	0.00%	0.00%	0.00%	0.00%	0.00%	0.00%	0.00%
Parvibacter	0.00%	0.00%	0.00%	0.00%	0.00%	0.00%	0.00%	0.00%	0.00%
Senegalimassilia	[redacted]	0.00%	0.00%	[redacted]	0.00%	[redacted]	[redacted]		0.00%
U. m. of Coriobacteriaceae family	0.00%	-0.02%	0.00%	-0.01%	0.00%	-0.01%	-0.03%	-0.05%	-0.03%
Corynebacterium	0.00%	0.00%	0.00%	[redacted]		0.00%	0.00%	0.00%	0.00%
Microbacterium	0.00%	0.00%	0.00%	[redacted]		0.00%	0.00%	[redacted]	0.00%
U. m. of Microbacteriaceae family	0.00%	0.00%	0.00%	[redacted]			0.00%	[redacted]	
Rothia	0.00%	0.00%	0.00%	[redacted]		0.00%	0.00%	[redacted]	0.01%
U. m. of Micrococcaceae family	0.00%	0.00%	0.01%	0.00%	0.00%	0.00%	0.00%	0.00%	0.01%
Propionibacterium	-0.01%	-0.02%	-0.01%	0.00%	0.00%	0.00%	0.02%	0.00%	0.00%
U. m. of Actinobacteria phylum	0.00%	0.00%	0.00%	[redacted]	0.00%	0.00%	0.00%	0.00%	0.00%
Bacteroides	-0.39%	-0.08%	-0.07%	-0.29%	-0.40%	-0.30%	-0.18%	-0.38%	0.05%
U. m. of Bacteroidales BS11 gut group family	0.00%	0.00%	0.00%	[redacted]	0.00%	0.00%	[redacted]		0.00%
U. m. of Bacteroidales RF16 group family	-0.03%	-0.01%	0.01%	-0.01%	-0.01%	-0.01%	0.00%	0.00%	0.00%
U. m. of Bacteroidales S24-7 group family	5.53%	-5.03%	-5.79%	-3.25%	0.45%	-3.41%	2.09%	0.86%	-2.35%
Barnesiella	0.00%	0.00%	0.01%	0.00%	0.00%	0.01%	0.00%	0.00%	0.01%
Butyricimonas	0.00%	0.01%	0.00%	0.00%	0.00%	0.00%	0.00%	0.01%	0.01%
Coprobacter	[redacted]		0.00%	[redacted]		0.00%	[redacted]		0.00%
Odoribacter	-0.01%	-0.01%	0.00%	0.02%	0.04%	0.01%	-0.01%	-0.02%	0.00%
Parabacteroides	0.62%	0.80%	-0.11%	-0.07%	0.31%	0.02%	0.88%	0.73%	-0.02%
U. m. of Porphyromonadaceae family	-0.01%	-0.01%	0.02%	0.00%	0.01%	0.02%	0.00%	0.00%	0.02%
Alloprevotella	-0.43%	-0.39%	1.27%	-0.38%	-0.35%	0.39%	-0.17%	0.21%	1.20%
Paraprevotella	-0.01%	0.15%	-0.01%	-0.01%	-0.01%	-0.01%	0.00%	0.00%	0.00%
Prevotella 1	0.95%	3.47%	-0.71%	0.10%	-0.32%	-0.99%	1.80%	3.98%	-1.95%
Prevotella 2	0.00%	0.02%	0.00%	0.00%	0.01%	0.00%	0.00%	0.01%	-0.01%
Prevotella 7	-0.40%	0.29%	-0.73%	-0.59%	-0.36%	-0.13%	0.85%	1.28%	-0.11%
Prevotella 9	0.01%	-0.02%	0.07%	0.02%	0.01%	0.06%	0.07%	0.00%	0.08%
Prevotellaceae Ga6A1 group	[redacted]		0.00%	[redacted]		0.00%	[redacted]		0.00%

Prevotellaceae NK3B31 group	1.35%	1.32%	6.75%	5.42%	0.97%	2.36%	2.58%	2.75%	7.56%
Prevotellaceae UCG-001	-0.45%	0.44%	1.15%	0.08%	0.39%	1.33%	-0.27%	0.63%	1.08%
Prevotellaceae UCG-003	-0.04%	0.07%	0.10%	0.00%	0.07%	-0.01%	-0.07%	0.02%	-0.03%
Prevotellaceae UCG-004	0.00%	0.00%	0.00%	0.00%	0.00%	0.00%	0.00%	0.00%	0.00%
Prevotella	-0.01%	0.05%	0.00%	0.05%	0.05%	0.02%	-0.05%	0.03%	-0.04%
U. m. of Prevotellaceae family	4.41%	16.42%	-0.70%	-0.11%	-0.51%	0.56%	6.38%	12.68%	-4.77%
Alistipes	-0.04%	-0.04%	0.00%	-0.01%	0.19%	0.06%	-0.07%	-0.06%	0.00%
Rikenellaceae RC9 gut group	-0.16%	0.07%	0.15%	-0.06%	0.25%	0.12%	-0.06%	-0.04%	0.15%
Rikenella	-0.01%	-0.01%	-0.01%	0.00%	0.00%	0.00%	-0.02%	-0.02%	-0.02%
U. m. of Rikenellaceae family	-0.01%	-0.01%	0.00%	0.00%	0.00%	0.00%	0.00%	0.00%	0.00%
U. m. of Bacteroidales order	-0.57%	-0.53%	0.11%	-0.22%	-0.53%	-0.37%	-0.38%	-0.59%	-0.27%
U. m. of Cytophagales order	-0.02%	-0.02%	0.01%	0.02%	0.02%	0.02%	-0.03%	-0.03%	-0.01%
Cloacibacterium	0.00%	0.00%	0.00%	0.00%	0.00%	0.00%	0.00%	0.00%	0.00%
Flavobacterium	0.00%	0.00%	0.00%	[redacted]			0.00%	0.00%	0.00%
U. m. of Bacteroidetes phylum	-0.02%	-0.01%	0.00%	0.00%	0.01%	0.01%	-0.02%	-0.01%	0.01%
U. m. of AKIW781 family	-0.01%	-0.01%	-0.01%	[redacted]			[redacted]		
U. m. of Cyanobacteria phylum	0.01%	-0.01%	0.00%	0.00%	0.00%	0.00%	0.07%	0.01%	0.01%
U. m. of Gastranaerophilales order	-0.01%	0.09%	0.13%	0.01%	0.18%	0.23%	0.03%	0.27%	0.07%
Mucispirillum	0.00%	0.00%	0.00%	0.00%	0.00%	0.00%	0.00%	0.00%	0.00%
Anoxybacillus	0.00%	0.00%	0.00%	[redacted]			0.00%	[redacted]	
Bacillus	-0.01%	-0.03%	-0.03%	-0.01%	0.01%	-0.02%	0.04%	-0.03%	-0.03%
Terribacillus	0.00%	0.00%	0.00%	0.00%	0.00%	0.00%	0.00%	0.00%	0.00%
Gemella	0.00%	0.00%	0.00%	0.00%	0.00%	0.00%	0.00%	-0.01%	0.00%
Brochothrix	0.00%	0.00%	0.00%	[redacted]		0.00%	[redacted]		0.00%
Listeria	0.00%	0.00%	0.00%			0.00%			0.00%
Staphylococcus	0.03%	0.00%	0.01%	0.00%	0.00%	0.01%	0.04%	0.01%	0.01%
Catabacter	0.00%	0.00%	0.00%	0.00%	0.00%	0.00%	0.00%	0.00%	0.00%
Christensenellaceae R-7 group	-0.80%	-1.11%	-0.95%	-0.45%	0.22%	0.07%	-0.29%	-0.32%	-0.09%
U. m. of Christensenellaceae family	-0.05%	-0.05%	-0.01%	-0.01%	0.01%	0.00%	-0.04%	-0.03%	0.01%
Clostridium sensu stricto 1	-0.02%	-0.03%	0.01%	0.00%	0.00%	0.01%	0.00%	-0.01%	0.01%
U. m. of Clostridiales vadinBB60 group family	-0.04%	0.02%	0.04%	0.00%	0.07%	0.03%	-0.05%	-0.04%	0.05%
Defluviitaleaceae UCG-011	0.04%	0.02%	0.02%	0.00%	0.01%	0.02%	0.00%	0.00%	0.04%
Anaerofustis	0.00%	0.00%	0.00%	0.00%	0.00%	0.00%	0.00%	0.00%	0.00%
Anaerovorax	-0.04%	-0.04%	0.02%	-0.02%	-0.01%	-0.01%	-0.07%	-0.07%	-0.01%
Eubacterium nodatum group (Clostridiales order, Family XIII family)	-0.08%	-0.07%	-0.04%	-0.05%	-0.04%	-0.03%	-0.10%	-0.12%	-0.09%
Family XIII AD3011 group (Clostridiales order)	-0.06%	-0.09%	0.01%	0.00%	0.02%	0.00%	-0.08%	-0.12%	-0.03%
Family XIII UCG-001 (Clostridiales order)	-0.01%	-0.02%	0.00%	0.01%	0.01%	0.01%	-0.02%	-0.02%	-0.01%
Mogibacterium	-0.01%	-0.01%	-0.01%	0.00%	0.01%	0.00%	0.00%	0.00%	-0.01%
U. m. of Family XIII family (Clostridiales order)	0.00%	-0.02%	0.01%	0.02%	0.02%	-0.01%	-0.02%	-0.06%	-0.02%
Acetatifactor	-0.07%	-0.07%	-0.02%	0.07%	-0.04%	0.09%	-0.07%	-0.07%	0.38%
Anaerostipes	0.00%	-0.05%	-0.03%	0.06%	0.05%	0.09%	0.01%	0.00%	0.09%

Blautia	-0.06%	-0.04%	-0.04%	-0.03%	-0.01%	-0.02%	0.07%	0.01%	0.02%
Butyrivibrio 2	0.00%	0.00%	0.01%	0.00%	[redacted]	0.01%	[redacted]	[redacted]	0.01%
Butyrivibrio	0.00%	0.00%	0.02%	-0.05%	-0.06%	-0.03%	-0.01%	-0.01%	0.00%
Coprococcus 2	0.00%	0.00%	0.00%	0.00%	0.00%	0.00%	0.00%	0.00%	0.00%
Coprococcus 3	-0.17%	-0.16%	0.00%	-0.04%	0.06%	-0.04%	-0.10%	-0.09%	-0.03%
Dorea	-0.10%	-0.10%	-0.09%	-0.05%	-0.06%	-0.03%	-0.05%	-0.06%	-0.05%
Eisenbergiella	-0.02%	0.04%	-0.02%	0.02%	-0.01%	-0.03%	-0.03%	-0.05%	-0.04%
Eubacterium eligens group (Lachnospiraceae family)	0.00%	0.05%	0.01%	0.00%	-0.01%	0.00%	0.01%	0.00%	0.02%
Eubacterium fissicatena group (Lachnospiraceae family)	0.00%	0.00%	0.00%	0.00%	0.00%	0.00%	0.00%	0.00%	0.01%
Eubacterium hallii group (Lachnospiraceae family)	-0.08%	-0.05%	0.14%	0.00%	-0.04%	0.04%	-0.12%	-0.11%	0.13%
Eubacterium ruminantium group (Lachnospiraceae family)	0.00%	-0.01%	0.00%	0.00%	0.00%	0.00%	0.01%	0.00%	0.00%
Eubacterium uniforme	0.00%	0.00%	0.00%	[redacted]	[redacted]	[redacted]	0.00%	0.00%	[redacted]
Eubacterium ventriosum group (Lachnospiraceae family)	1.00%	1.89%	-0.01%	0.02%	0.08%	0.05%	1.84%	0.56%	0.02%
Fusicatenibacter	0.00%	0.06%	0.00%	0.00%	0.00%	0.00%	0.00%	0.00%	0.00%
Howardella	0.00%	0.00%	0.00%	0.00%	0.00%	0.00%	0.00%	0.00%	0.00%
Hungatella	0.00%	0.00%	0.00%	0.00%	0.00%	0.00%	0.00%	[redacted]	0.00%
Lachnoanaerobaculum	0.00%	0.68%	0.41%	-0.04%	0.26%	0.48%	-0.05%	0.38%	0.77%
Lachnoclostridium 10	0.00%	0.00%	0.00%	0.00%	0.00%	0.00%	0.00%	0.00%	0.00%
Lachnoclostridium	0.00%	0.00%	0.00%	0.00%	0.00%	0.00%	0.01%	0.00%	0.01%
Lachnospiraceae FCS020 group	-0.01%	-0.01%	0.00%	-0.01%	-0.01%	0.00%	-0.01%	-0.01%	0.00%
Lachnospiraceae ND3007 group	0.00%	0.00%	0.00%	0.00%	0.00%	0.00%	0.00%	0.00%	0.00%
Lachnospiraceae NK3A20 group	-0.01%	-0.01%	-0.01%	-0.01%	0.01%	-0.01%	-0.02%	-0.02%	-0.02%
Lachnospiraceae NK4A136 group	-1.54%	-1.32%	1.89%	-0.01%	0.27%	1.92%	-1.92%	-1.81%	2.03%
Lachnospiraceae NK4B4 group	-0.07%	-0.07%	-0.06%	0.00%	0.00%	0.00%	-0.01%	-0.01%	-0.01%
Lachnospiraceae UCG-001	-0.02%	0.02%	0.00%	0.05%	0.16%	-0.02%	0.03%	0.03%	0.04%
Lachnospiraceae UCG-006	-0.02%	0.01%	-0.01%	0.04%	0.02%	-0.01%	-0.03%	-0.06%	0.00%
Lachnospiraceae UCG-010	-0.01%	0.00%	0.00%	0.00%	0.00%	0.00%	-0.01%	-0.01%	0.00%
Lachnospiraceae XPB1014 group	-0.02%	-0.02%	0.00%	0.01%	-0.02%	-0.02%	-0.03%	-0.03%	-0.01%
Lachnospira	-0.05%	-0.04%	0.00%	-0.02%	-0.05%	-0.03%	0.00%	0.00%	0.10%
Marvinbryantia	0.00%	0.11%	0.00%	0.02%	-0.02%	-0.02%	0.00%	0.00%	0.00%
Mobilitalea	0.00%	0.00%	0.00%	0.00%	0.00%	0.00%	0.00%	0.00%	0.00%
Moryella	0.00%	0.00%	0.00%	0.00%	0.00%	0.00%	0.00%	0.00%	0.00%
Oribacterium	-0.01%	-0.01%	0.00%	-0.01%	-0.01%	-0.01%	-0.02%	-0.02%	0.01%
Roseburia	-0.02%	0.00%	0.01%	-0.01%	-0.01%	0.01%	-0.03%	-0.04%	-0.03%
Ruminococcus gnavus group (Lachnospiraceae family)	-0.37%	0.84%	-0.42%	-0.11%	-0.44%	-0.34%	-0.37%	-0.48%	-0.68%
Syntrophococcus	0.00%	0.00%	0.00%	0.00%	0.00%	0.00%	0.00%	0.00%	0.00%
Tyzzerella 3	-0.01%	-0.01%	0.00%	0.00%	0.01%	0.01%	0.01%	-0.01%	0.00%
Tyzzerella 4	0.00%	0.00%	0.00%	0.00%	0.00%	0.00%	-0.01%	-0.01%	-0.01%
Tyzzerella	-0.08%	-0.11%	-0.05%	-0.04%	-0.07%	-0.05%	-0.05%	-0.13%	-0.07%
U. m. of Lachnospiraceae family	-4.54%	-3.84%	0.82%	4.72%	0.12%	1.54%	-7.52%	-8.16%	3.26%
Peptococcus	0.00%	0.00%	0.01%	0.00%	0.00%	0.00%	0.00%	0.01%	0.02%

U. m. of Peptococcaceae family	-0.01%	-0.01%	0.00%	0.00%	-0.01%	0.01%	-0.02%	-0.02%	-0.01%
Peptoclostridium	0.00%	0.00%	0.00%	[redacted]	[redacted]	0.00%	[redacted]	[redacted]	0.00%
Peptostreptococcus	[redacted]	[redacted]	0.00%	[redacted]	[redacted]	0.00%	[redacted]	[redacted]	0.00%
Romboutsia	-0.44%	-0.45%	-0.25%	-0.10%	0.05%	-0.12%	-0.20%	-0.21%	-0.04%
Terrisporobacter	0.00%	0.00%	0.00%	0.00%	0.00%	0.00%	0.00%	0.00%	0.00%
U. m. of Peptostreptococcaceae family	-0.01%	-0.01%	-0.01%	0.00%	0.00%	0.00%	-0.01%	-0.01%	-0.01%
Anaerofilum	0.00%	-0.01%	0.00%	-0.01%	0.00%	0.01%	0.00%	-0.01%	0.01%
Anaerotruncus	-0.53%	-0.39%	-0.26%	-0.06%	0.06%	0.07%	-0.51%	-0.56%	-0.13%
Butyricicoccus	-0.06%	-0.05%	-0.03%	-0.03%	-0.02%	-0.02%	0.01%	-0.05%	0.03%
Candidatus Soleaferrea	0.00%	0.00%	0.00%	0.00%	0.00%	0.00%	0.00%	-0.01%	0.00%
Caproiciproducens	0.00%	0.00%	0.00%	0.00%	0.00%	0.00%	0.00%	0.00%	0.00%
Eubacterium coprostanoligenes group (Ruminococcaceae family)	-0.10%	-0.81%	0.44%	-0.04%	0.28%	0.30%	-0.34%	-0.71%	0.12%
Faecalibacterium	-0.04%	0.54%	0.05%	0.00%	-0.03%	0.05%	0.08%	0.01%	0.12%
Flavonifractor	-0.02%	-0.03%	-0.01%	0.01%	0.02%	0.00%	-0.03%	-0.03%	-0.01%
Hydrogenoanaerobacterium	-0.08%	-0.11%	-0.04%	-0.03%	0.01%	-0.02%	-0.03%	-0.12%	-0.03%
Intestinimonas	-0.23%	-0.25%	-0.05%	0.01%	0.01%	0.07%	-0.20%	-0.32%	-0.02%
Oscillibacter	-0.29%	-0.16%	-0.04%	-0.20%	-0.06%	-0.11%	-0.39%	-0.47%	-0.01%
Oscillospira	0.00%	-0.01%	-0.01%	0.00%	0.00%	0.00%	0.00%	-0.01%	0.01%
Papillibacter	-0.06%	-0.01%	-0.02%	0.04%	0.10%	0.04%	-0.07%	-0.08%	-0.01%
Ruminiclostridium 1	0.00%	0.00%	0.00%	0.00%	0.00%	0.00%	0.00%	0.00%	0.00%
Ruminiclostridium 5	3.01%	0.97%	0.72%	-0.03%	-0.02%	0.24%	0.79%	0.33%	0.09%
Ruminiclostridium 6	-0.56%	-0.27%	-0.47%	-0.05%	0.09%	0.72%	-0.67%	-0.16%	0.07%
Ruminiclostridium 9	-1.03%	-1.06%	0.21%	-0.22%	-0.06%	-0.06%	-1.66%	-2.02%	-0.84%
Ruminiclostridium	-0.08%	-0.08%	-0.03%	-0.03%	-0.03%	-0.02%	-0.07%	-0.10%	0.03%
Ruminococcaceae NK4A214 group	-0.08%	-0.16%	-0.05%	-0.10%	-0.03%	-0.03%	-0.04%	-0.04%	0.02%
Ruminococcaceae UCG-002	-0.08%	-0.04%	0.04%	-0.09%	-0.17%	-0.12%	-0.19%	-0.25%	0.08%
Ruminococcaceae UCG-003	0.00%	0.00%	0.00%	0.00%	0.00%	0.00%	0.00%	[redacted]	0.00%
Ruminococcaceae UCG-004	0.00%	0.00%	0.00%	0.00%	0.00%	0.00%	-0.01%	0.00%	0.00%
Ruminococcaceae UCG-005	0.75%	-0.84%	-2.15%	-1.24%	0.84%	-0.35%	6.87%	2.85%	0.39%
Ruminococcaceae UCG-007	0.00%	0.00%	0.01%	0.00%	0.00%	0.00%	0.00%	0.00%	0.00%
Ruminococcaceae UCG-009	-0.22%	-0.17%	-0.11%	-0.03%	-0.02%	0.00%	-0.14%	-0.16%	0.03%
Ruminococcaceae UCG-010	-0.09%	-0.12%	-0.08%	0.01%	0.05%	0.01%	-0.04%	-0.09%	-0.02%
Ruminococcaceae UCG-013	-0.24%	-0.26%	0.12%	-0.20%	0.01%	-0.12%	-0.35%	-0.42%	0.01%
Ruminococcaceae UCG-014	-1.35%	-1.78%	-0.47%	-0.95%	-1.11%	-1.16%	-1.50%	1.16%	-1.46%
Ruminococcaceae V9D2013 group	0.00%	0.00%	0.00%	0.00%	0.00%	0.00%	-0.01%	0.00%	0.00%
Ruminococcus 1	-3.34%	-2.90%	-1.52%	0.05%	1.15%	0.34%	-0.21%	-1.71%	-0.26%
Ruminococcus 2	0.07%	0.01%	0.04%	-0.04%	-0.01%	0.02%	-0.07%	-0.15%	-0.11%
Sporobacter	0.00%	-0.01%	0.01%	0.01%	0.01%	0.02%	-0.02%	-0.04%	-0.02%
Subdoligranulum	-0.01%	-0.01%	0.02%	0.01%	0.00%	0.01%	0.01%	0.00%	0.02%
U. m. of Ruminococcaceae family	0.03%	-1.18%	-0.07%	-0.49%	-0.55%	-0.40%	-2.23%	-2.76%	-1.34%
U. m. of Clostridiales order	-0.13%	-0.18%	-0.06%	-0.11%	-0.02%	-0.11%	-0.08%	-0.25%	-0.12%

Allobaculum	1.30%	-0.01%	-0.07%	0.04%	0.08%	-0.04%	0.47%	0.04%	-0.01%
Candidatus Stoquefichus	0.52%	0.32%	0.00%	0.00%	0.00%	0.00%	0.47%	0.12%	0.00%
Catenibacterium	[redacted]		0.00%	[redacted]		0.00%	0.00%	[redacted]	0.00%
Dielma	0.00%	0.00%	0.00%	0.00%	0.00%	0.00%	0.00%	0.00%	0.00%
Erysipelatoclostridium	0.00%	0.00%	0.00%	0.00%	0.00%	0.00%	0.00%	0.00%	0.00%
Erysipelotrichaceae UCG-002	0.00%	0.00%	0.00%	[redacted]		0.00%	[redacted]		0.00%
Erysipelotrichaceae UCG-003	0.02%	0.07%	-0.03%	-0.05%	0.01%	-0.02%	0.30%	0.04%	-0.01%
Faecalibaculum	0.01%	0.00%	0.00%	0.00%	0.01%	0.00%	0.00%	0.00%	0.01%
Holdemanella	0.00%	[redacted]	0.00%	[redacted]	0.00%	0.00%	0.00%	0.00%	0.00%
Turicibacter	-0.06%	-0.05%	-0.03%	-0.08%	-0.04%	-0.09%	-0.06%	-0.06%	-0.05%
U. m. of Erysipelotrichaceae family	1.03%	0.01%	0.05%	-0.07%	0.23%	-0.09%	0.36%	0.13%	-0.01%
Enterococcus	0.03%	0.03%	0.04%	0.00%	0.00%	0.03%	0.03%	0.02%	0.03%
Lactobacillus	1.14%	-1.95%	-0.82%	0.49%	-1.96%	-1.29%	-0.15%	-1.01%	-1.21%
Lactococcus	-0.01%	-0.01%	0.00%	0.00%	0.00%	0.00%	0.00%	0.00%	0.00%
Streptococcus	0.01%	-0.03%	0.06%	-0.01%	-0.01%	0.02%	-0.07%	-0.10%	-0.03%
Acidaminococcus	0.00%	0.00%	0.00%	0.00%	[redacted]	0.00%	0.00%	0.00%	0.00%
Phascolarctobacterium	0.00%	0.05%	0.02%	0.00%	0.00%	0.02%	0.00%	0.00%	0.01%
Anaerovibrio	0.25%	0.26%	0.06%	-0.02%	0.02%	-0.02%	0.29%	0.03%	0.00%
Dialister	0.00%	0.00%	0.01%	0.00%	0.00%	0.00%	0.01%	0.00%	0.00%
Megamonas	0.00%	0.00%	0.01%	0.00%	0.00%	0.00%	[redacted]	0.00%	0.00%
Megasphaera	0.00%	0.00%	0.00%	[redacted]		0.00%	[redacted]		0.00%
Quinella	0.00%	0.08%	0.12%	-0.04%	-0.03%	-0.05%	-0.18%	-0.16%	-0.10%
Veillonella	-0.01%	-0.02%	0.02%	-0.01%	-0.01%	0.00%	-0.03%	-0.02%	0.01%
U. m. of Veillonellaceae family	0.05%	0.12%	0.20%	-0.06%	0.14%	0.17%	-0.04%	-0.07%	-0.03%
U. m. of Selenomonadales order	0.01%	0.00%	0.00%	0.00%	0.00%	0.00%	0.00%	0.00%	0.00%
U. m. of Firmicutes phylum	0.00%	0.00%	0.02%	0.00%	0.01%	0.00%	-0.01%	0.00%	0.00%
Cetobacterium	0.00%	0.00%	0.00%	[redacted]			[redacted]		0.00%
Fusobacterium	-0.02%	-0.02%	0.01%	-0.01%	0.00%	0.02%	0.01%	0.01%	0.02%
Anaerobiospirillum	0.00%	0.00%	0.00%	0.00%	0.00%	0.00%	0.00%	0.00%	0.00%
Succinivibrio	-0.01%	-0.01%	0.04%	0.00%	0.00%	0.03%	0.00%	0.00%	0.04%
U. m. of Succinivibrionaceae family	0.00%	0.00%	0.02%	0.00%	0.00%	0.00%	0.00%	0.00%	0.01%
U. m. of Aeromonadales order	0.00%	0.00%	0.00%	0.00%	0.00%	0.00%	0.00%	0.00%	0.00%
Parasutterella	-0.07%	0.05%	-0.09%	-0.05%	0.17%	0.02%	0.05%	0.08%	0.00%
Sutterella	0.00%	0.00%	0.01%	0.00%	[redacted]	0.01%	0.01%	0.00%	0.01%
U. m. of CM1G08 family	0.00%	0.00%	0.00%	0.00%	0.00%	0.00%	[redacted]		0.00%
U. m. of Burkholderiales order	0.00%	0.00%	0.00%	0.00%	0.02%	0.01%	0.00%	0.00%	0.01%
Helicobacter	-0.10%	-0.03%	0.00%	-0.12%	-0.03%	-0.10%	-0.04%	-0.02%	0.08%
Caulobacter	0.00%	[redacted]	0.00%	0.00%	0.00%	0.00%	0.00%	0.00%	0.00%
U. m. of Cellvibrionales order	0.00%	0.00%	0.00%	0.00%	0.00%	0.00%	0.00%	0.00%	0.00%
Bilophila	0.00%	0.00%	0.00%	0.00%	0.00%	0.00%	0.00%	0.00%	0.00%
Desulfovibrio	-0.05%	-0.10%	0.20%	0.00%	0.14%	0.14%	-0.48%	-0.48%	-0.15%

Citrobacter	0.00%	0.00%	0.00%	0.00%	0.00%	0.00%	0.00%	0.00%	0.00%
Enterobacter	0.00%	-0.01%	0.02%	0.00%	0.00%	0.01%	0.01%	0.00%	0.02%
Escherichia-Shigella	0.03%	-0.03%	0.10%	0.02%	0.01%	0.07%	0.03%	0.01%	0.07%
Hafnia-Obesumbacterium	[redacted]	[redacted]	0.00%	0.00%	0.00%	0.00%	0.00%	[redacted]	0.00%
Klebsiella	0.00%	0.00%	0.01%	0.00%	0.00%	0.01%	0.00%	0.00%	0.01%
Salmonella	0.00%	0.00%	0.00%	0.00%	0.00%	0.00%	0.00%	[redacted]	0.00%
Serratia	0.00%	0.00%	0.00%	0.00%	[redacted]	0.00%	0.00%	0.00%	0.00%
U. m. of Enterobacteriaceae family	0.00%	0.00%	0.01%	0.00%	0.00%	0.00%	0.00%	0.00%	0.01%
Haemophilus	0.00%	[redacted]	0.00%	0.00%	0.00%	0.00%	0.00%	[redacted]	[redacted]
Necropsobacter	0.00%	0.01%	0.00%	0.00%	0.00%	0.00%	0.00%	0.00%	0.00%
Pasteurella	-0.01%	0.00%	0.01%	0.00%	0.00%	0.00%	0.00%	0.00%	0.00%
U. m. of Pasteurellaceae family	[redacted]	0.00%	[redacted]	0.00%	0.00%	0.00%	[redacted]	0.00%	0.00%
Acinetobacter	0.00%	0.00%	0.00%	[redacted]	0.00%	0.00%	0.00%	[redacted]	0.00%
Enhydrobacter	0.01%	[redacted]	[redacted]	0.00%	0.00%	0.00%	0.00%	0.00%	[redacted]
Paracoccus	0.00%	[redacted]	0.00%	0.00%	[redacted]	0.00%	0.00%	0.00%	[redacted]
U. m. of Rhodospirillaceae family	-0.07%	0.20%	0.11%	-0.02%	0.20%	0.00%	0.20%	0.27%	0.19%
Vibrio	0.00%	0.00%	0.00%	[redacted]	[redacted]	0.00%	[redacted]	[redacted]	0.00%
Stenotrophomonas	0.00%	0.00%	0.00%	0.00%	0.00%	0.00%	0.00%	0.00%	0.00%
U. m. of Proteobacteria phylum	-0.70%	-0.30%	-0.67%	0.07%	-0.64%	-0.19%	-1.64%	-1.24%	-0.01%
U. m. of Saccharibacteria phylum	0.03%	-1.35%	0.65%	-0.37%	-0.02%	-0.77%	-1.67%	-2.53%	-1.42%
Treponema 2	0.01%	0.30%	0.08%	0.00%	0.11%	0.09%	-0.05%	1.00%	0.19%
Anaeroplasma	0.00%	0.00%	0.00%	0.00%	0.01%	0.00%	-0.01%	-0.01%	-0.01%
U. m. of Mollicutes RF9 order	-1.01%	-1.13%	0.45%	-0.58%	-0.22%	-0.87%	-0.66%	-0.84%	-0.61%
U. m. of Mycoplasmataceae family	0.01%	0.02%	0.01%	0.00%	0.05%	0.02%	-0.01%	0.04%	0.01%
U. m. of Opitutae vadinHA64 order	0.00%	0.00%	0.00%	0.00%	0.00%	0.00%	[redacted]	0.00%	[redacted]
U. m. of Verrucomicrobia phylum	-0.02%	0.00%	-0.01%	0.00%	0.00%	-0.01%	0.00%	0.00%	0.00%
Akkermansia	0.00%	0.00%	0.02%	0.00%	0.00%	0.00%	0.02%	0.00%	0.01%
U. m. of Verrucomicrobiaceae family	0.00%	[redacted]	0.00%	[redacted]	0.00%	[redacted]	0.00%	[redacted]	0.00%
U. m. of Bacteria kingdom	-0.03%	0.11%	-0.01%	-0.05%	0.12%	-0.07%	-0.11%	-0.04%	-0.14%
Others	-0.01%	0.02%	0.01%	-0.02%	0.04%	-0.02%	-0.03%	-0.02%	-0.05%

Taxonomy	Relative variation (%) at genus level								
	CG			Breve2L			CG+Breve2L		
	T1-T0	T2-T0	T3-T0	T1-T0	T2-T0	T3-T0	T1-T0	T2-T0	T3-T0
U. m. of Euryarchaeota phylum	-89.55%	Absent in T2	Absent in T3	Absent in both	Absent in T0	Absent in both	-33.18%	-42.40%	-91.48%
Actinomyces	56.66%	-54.97%	147.92%	-47.61%	-19.02%	-0.42%	-59.81%	-74.57%	-68.13%
Bifidobacterium	75.50%	-60.52%	293.53%	306.16%	652.19%	350.85%	75.76%	-35.66%	113.47%
Gardnerella	181.39%	Absent in T2	40.28%	Absent in both	Absent in both	Absent in both	119.76%	Absent in T2	52.17%
Adlercreutzia	106.05%	-21.99%	-20.06%	52.33%	75.95%	27.47%	34.26%	-59.29%	-34.90%
Atopobium	63.36%	-70.64%	45.15%	-72.74%	-2.74%	-50.19%	-39.67%	-74.06%	12.44%
Collinsella	-42.29%	34.54%	296.94%	6.04%	320.36%	601.60%	80.79%	133.55%	653.32%
Coriobacteriaceae UCG-002	453.36%	225.55%	-1.42%	-16.08%	83.61%	-57.35%	113.05%	308.99%	-38.60%
Enterorhabdus	-62.17%	-80.15%	20.98%	-9.24%	92.78%	93.25%	-86.52%	-83.57%	-38.93%
Gordonibacter	14.65%	-58.41%	3.54%	-15.49%	76.54%	Absent in T3	63.63%	100.29%	27.93%
Parvibacter	Absent in T1	-77.51%	2.28%	Absent in T0	Absent in T0	Absent in T0	Absent in T1	Absent in T2	56.58%
Senegalimassilia	Absent in both	Absent in T0	Absent in T0	Absent in both	Absent in T0	Absent in both	Absent in both	Absent in both	Absent in T0
U. m. of Coriobacteriaceae family	8.62%	-59.46%	12.89%	-31.95%	-12.19%	-23.90%	-60.07%	-82.40%	-60.05%
Corynebacterium	-64.56%	93.03%	-79.21%	Absent in both	Absent in both	Absent in T0	1099.21%	Absent in T2	239.11%
Microbacterium	40.79%	Absent in T2	52.10%	Absent in both	Absent in both	Absent in T0	Absent in T0	Absent in both	Absent in T0
U. m. of Microbacteriaceae family	169.18%	Absent in T2	-28.63%	Absent in both	Absent in both	Absent in both	Absent in T0	Absent in both	Absent in both
Rothia	Absent in T1	Absent in T2	287.00%	Absent in both	Absent in both	Absent in T0	Absent in T0	Absent in both	Absent in T0
U. m. of Micrococcaceae family	0.52%	-33.72%	151.10%	-13.90%	-26.96%	2.69%	8.53%	1.71%	67.53%
Propionibacterium	-32.35%	-81.06%	-63.70%	1.24%	227.53%	340.59%	1095.45%	42.51%	248.39%
U. m. of Actinobacteria phylum	Absent in T1	-29.00%	-35.23%	Absent in both	Absent in T0	Absent in T0	-78.22%	-76.80%	-42.65%
Bacteroides	-72.86%	-15.52%	-12.56%	-40.33%	-55.61%	-41.35%	-40.16%	-84.05%	12.17%
U. m. of Bacteroidales BS11 gut group family	Absent in T1	Absent in T2	203.87%	Absent in both	Absent in T0	Absent in T0	Absent in both	Absent in both	Absent in T0
U. m. of Bacteroidales RF16 group family	-74.18%	-28.80%	32.53%	-30.79%	-41.53%	-42.92%	-51.37%	9.28%	-1.29%
U. m. of Bacteroidales S24-7 group family	19.64%	-17.83%	-20.56%	-13.84%	1.93%	-14.51%	8.99%	3.68%	-10.11%
Barnesiella	-50.60%	-84.97%	416.99%	-22.29%	Absent in T2	288.83%	61.03%	Absent in T2	245.25%
Butyricimonas	-27.40%	91.28%	-4.88%	8.01%	28.26%	-15.72%	86.42%	210.88%	174.99%
Coprobacter	Absent in both	Absent in both	Absent in T0	Absent in both	Absent in both	Absent in T0	Absent in both	Absent in both	Absent in T0
Odoribacter	-30.01%	-38.45%	-7.14%	40.39%	76.10%	19.92%	-29.73%	-54.63%	8.88%
Parabacteroides	139.78%	179.37%	-23.94%	-17.78%	79.16%	4.05%	275.16%	226.56%	-4.81%
U. m. of Porphyromonadaceae family	-65.88%	-70.91%	294.93%	84.88%	351.74%	565.88%	-41.48%	102.14%	1327.11%
Alloprevotella	-35.61%	-32.08%	105.40%	-31.64%	-29.43%	32.98%	-16.36%	20.52%	118.62%
Paraprevotella	-44.97%	585.49%	-25.43%	-63.33%	-76.82%	-38.25%	-14.20%	-32.91%	42.56%
Prevotella 1	27.31%	100.03%	-20.32%	1.99%	-6.56%	-20.53%	46.71%	103.06%	-50.62%
Prevotella 2	29.53%	345.19%	-40.80%	29.50%	63.18%	6.31%	0.31%	142.93%	-75.32%
Prevotella 7	-20.78%	15.14%	-37.34%	-19.87%	-12.09%	-4.35%	52.81%	79.71%	-7.07%
Prevotella 9	44.50%	-85.11%	348.21%	837.16%	245.56%	2614.53%	1139.70%	66.94%	1222.17%
Prevotellaceae Ga6A1 group	Absent in both	Absent in both	Absent in T0	Absent in both	Absent in both	Absent in T0	Absent in both	Absent in both	Absent in T0
Prevotellaceae NK3B31 group	68.41%	66.84%	342.39%	235.33%	42.33%	102.46%	264.40%	281.04%	774.23%
Prevotellaceae UCG-001	-43.64%	42.17%	110.94%	9.49%	49.49%	167.09%	-31.30%	71.56%	123.53%
Prevotellaceae UCG-003	-21.12%	34.96%	46.76%	2.64%	46.68%	-6.44%	-34.85%	11.57%	-16.32%
Prevotellaceae UCG-004	Absent in T1	-75.51%	-68.34%	0.05%	43.07%	Absent in T3	Absent in T1	137.77%	-63.70%
Prevotella	-26.34%	193.71%	-3.43%	95.51%	95.98%	32.61%	-80.01%	40.53%	-65.60%
U. m. of Prevotellaceae family	39.08%	145.40%	-6.18%	-0.68%	-3.16%	3.45%	49.12%	97.59%	-36.69%
Alistipes	-33.95%	-33.70%	2.27%	-3.61%	134.62%	41.04%	-51.13%	-38.99%	-2.41%

Rikenellaceae RC9 gut group	-58.80%	25.77%	55.63%	-36.92%	162.69%	79.24%	-32.78%	-22.14%	86.31%
Rikenella	Absent in T1	Absent in T2	-92.80%	-42.94%	27.62%	14.41%	-97.24%	Absent in T2	-95.77%
U. m. of Rikenellaceae family	-90.54%	-95.28%	-77.18%	62.54%	163.13%	-8.41%	Absent in T1	-81.21%	-74.39%
U. m. of Bacteroidales order	-39.23%	-36.40%	7.52%	-14.63%	-34.48%	-24.04%	-28.37%	-44.62%	-20.29%
U. m. of Cytophagales order	-80.59%	-77.42%	29.76%	67.26%	62.37%	61.56%	-82.14%	-90.57%	-34.98%
Cloacibacterium	-90.85%	12.28%	Absent in T3	Absent in T1	Absent in T2	Absent in T3	-17.70%	-68.69%	Absent in T3
Flavobacterium	Absent in T0	Absent in T0	Absent in T0	Absent in both	Absent in both	Absent in both	537.76%	-40.18%	Absent in T3
U. m. of Bacteroidetes phylum	-85.94%	-68.60%	-16.39%	15.02%	33.82%	36.89%	-83.55%	-52.12%	49.55%
U. m. of AKIW781 family	Absent in T1	Absent in T2	Absent in T3	Absent in both	Absent in both	Absent in both	Absent in both	Absent in both	Absent in both
U. m. of Cyanobacteria phylum	94.15%	-84.73%	-26.35%	46.60%	43.13%	262.82%	5813.20%	998.01%	441.63%
U. m. of Gastranaerophilales order	-40.36%	250.90%	350.70%	21.56%	722.44%	909.58%	77.08%	814.55%	209.71%
Mucispirillum	-64.06%	215.77%	249.95%	-66.63%	-34.34%	82.35%	-77.10%	-1.56%	9.16%
Anoxybacillus	57.01%	Absent in T2	52.76%	Absent in both	Absent in both	Absent in both	Absent in T0	Absent in both	Absent in both
Bacillus	-23.25%	-90.52%	-85.09%	-38.65%	18.17%	-50.79%	121.12%	-85.27%	-85.76%
Terribacillus	-88.36%	Absent in T2	Absent in T3	-84.53%	5.86%	-83.77%	-57.30%	Absent in T2	-62.75%
Gemella	-88.26%	Absent in T2	87.17%	-91.49%	-69.90%	-30.75%	-47.82%	-91.37%	-56.03%
Brochothrix	Absent in T1	Absent in T2	189.24%	Absent in both	Absent in both	Absent in T0	Absent in both	Absent in both	Absent in T0
Listeria	Absent in T1	Absent in T2	217.30%	Absent in both	Absent in both	Absent in T0	Absent in both	Absent in both	Absent in T0
Staphylococcus	770.81%	8.62%	185.96%	37.68%	-34.59%	175.95%	2893.04%	772.24%	528.09%
Catabacter	27.02%	Absent in T2	362.83%	-67.78%	-36.27%	20.48%	Absent in T1	Absent in T2	150.96%
Christensenellaceae R-7 group	-57.10%	-79.33%	-68.04%	-49.01%	23.70%	7.33%	-52.73%	-57.52%	-15.84%
U. m. of Christensenellaceae family	-82.43%	-82.47%	-16.05%	-26.09%	30.30%	9.35%	-85.01%	-80.36%	13.51%
Clostridium sensu stricto 1	-81.60%	Absent in T2	41.39%	23.59%	-79.59%	262.37%	-3.36%	-89.89%	45.93%
U. m. of Clostridiales vadinBB60 group family	-64.14%	23.95%	60.19%	-0.42%	90.37%	35.28%	-62.62%	-42.36%	61.61%
Defluviitaleaceae UCG-011	1636.67%	919.25%	895.49%	68.28%	251.65%	485.77%	-18.85%	29.12%	429.79%
Anaerofustis	Absent in T1	Absent in T2	-61.63%	94.25%	46.72%	-61.39%	Absent in T1	Absent in T2	-10.21%
Anaerovorax	-73.09%	-77.58%	40.25%	-30.99%	-9.69%	-19.44%	-90.47%	-89.75%	-10.59%
Eubacterium nodatum group (Clostridiales order, Family XIII family)	-58.42%	-54.02%	-30.99%	-40.23%	-34.71%	-22.59%	-65.71%	-74.98%	-58.85%
Family XIII AD3011 group (Clostridiales order)	-45.11%	-61.31%	4.38%	-0.41%	14.79%	2.11%	-46.71%	-68.02%	-16.78%
Family XIII UCG-001 (Clostridiales order)	-30.63%	-58.77%	11.52%	28.22%	55.04%	41.96%	-41.99%	-59.63%	-15.00%
Mogibacterium	-40.76%	-64.25%	-70.16%	-79.84%	1012.69%	Absent in T3	-20.84%	-38.86%	-92.72%
U. m. of Family XIII family (Clostridiales order)	7.30%	-29.00%	16.08%	28.53%	36.60%	-7.99%	-24.28%	-68.17%	-19.03%
Acetatifactor	-93.63%	-88.05%	-28.29%	53.73%	-29.31%	70.02%	-88.05%	-82.34%	453.67%
Anaerostipes	7.52%	-82.50%	-45.72%	73.89%	58.53%	111.47%	15.60%	-14.14%	256.84%
Blautia	-53.36%	-39.68%	-38.56%	-54.82%	-27.06%	-30.96%	197.49%	20.90%	48.15%
Butyrivibrio 2	Absent in T1	Absent in T2	460.55%	Absent in T0	Absent in both	Absent in T0	Absent in both	Absent in both	Absent in T0
Butyrivibrio	Absent in T1	Absent in T2	692.72%	-76.19%	-89.67%	-52.47%	-97.37%	Absent in T2	-21.91%
Coprococcus 2	-25.24%	-77.79%	-22.89%	10.22%	-7.26%	108.60%	-43.29%	-2.41%	183.30%
Coprococcus 3	-83.33%	-78.20%	1.52%	-32.14%	44.28%	-33.00%	-81.19%	-70.15%	-26.39%
Dorea	-98.30%	Absent in T2	-89.35%	-85.49%	-93.96%	-54.83%	-96.06%	-98.18%	-88.16%
Eisenbergiella	-63.08%	137.69%	-64.66%	44.63%	-26.92%	-56.44%	-57.85%	-90.27%	-79.73%
Eubacterium eligens group (Lachnospiraceae family)	-35.65%	352.00%	94.72%	-12.88%	-70.81%	41.91%	814.24%	-25.58%	1234.04%
Eubacterium fissicatena group (Lachnospiraceae family)	-65.84%	-22.55%	15.69%	-38.52%	-46.95%	-32.54%	-75.01%	-46.02%	143.84%
Eubacterium hallii group (Lachnospiraceae family)	-88.19%	-62.03%	153.61%	0.26%	-34.29%	33.98%	-94.36%	-85.12%	101.19%
Eubacterium ruminantium group (Lachnospiraceae family)	-63.83%	-90.68%	53.72%	Absent in T1	-20.78%	144.09%	1549.35%	-29.29%	514.30%
Eubacterium uniforme	428.42%	218.90%	Absent in T3	Absent in both	Absent in both	Absent in both	Absent in T0	Absent in T0	Absent in both
Eubacterium ventriosum group (Lachnospiraceae family)	1099.51%	2072.48%	-7.79%	29.56%	122.70%	77.95%	2277.81%	689.34%	21.93%
Fusicatenibacter	Absent in T1	3540.10%	Absent in T3	-6.60%	-53.69%	-76.41%	-75.51%	Absent in T2	88.20%

Howardella	Absent in T1	Absent in T2	Absent in T3	Absent in T0	Absent in T0	Absent in T0	Absent in T1	Absent in T2	-58.77%
Hungatella	122.68%	Absent in T2	27.16%	-19.92%	35.23%	-26.35%	Absent in T0	Absent in both	Absent in T0
Lachnoanaerobaculum	-0.12%	148.05%	88.64%	-14.14%	83.51%	154.26%	-12.44%	87.01%	175.43%
Lachnoclostridium 10	-49.53%	Absent in T2	-95.95%	-34.60%	-73.43%	208.53%	-51.94%	-14.11%	Absent in T3
Lachnoclostridium	-46.38%	-90.10%	83.13%	43.38%	-27.21%	247.04%	336.56%	-49.48%	558.29%
Lachnospiraceae FCS020 group	-64.25%	-71.50%	-25.04%	-47.83%	-45.40%	-29.94%	-67.76%	-84.40%	-1.14%
Lachnospiraceae ND3007 group	-42.18%	-83.86%	-47.49%	-80.26%	-92.18%	-69.65%	62.45%	Absent in T2	-3.53%
Lachnospiraceae NK3A20 group	-84.97%	-94.00%	-69.06%	-27.25%	37.90%	-55.34%	-76.53%	-96.11%	-78.52%
Lachnospiraceae NK4A136 group	-92.54%	-79.71%	114.06%	-0.37%	17.90%	128.93%	-91.28%	-85.93%	96.22%
Lachnospiraceae NK4B4 group	-99.70%	-99.16%	-88.66%	-50.27%	2.74%	94.80%	-95.81%	Absent in T2	-74.38%
Lachnospiraceae UCG-001	-43.65%	45.74%	-5.71%	107.85%	323.23%	-34.47%	43.14%	48.69%	73.41%
Lachnospiraceae UCG-006	-45.97%	28.10%	-14.44%	65.86%	33.60%	-16.39%	-44.63%	-77.91%	3.86%
Lachnospiraceae UCG-010	-53.80%	-34.94%	13.63%	-27.46%	-6.36%	-7.01%	-79.43%	-48.72%	8.80%
Lachnospiraceae XPB1014 group	-84.37%	-87.54%	7.47%	30.06%	-56.84%	-53.13%	-86.78%	-82.49%	-16.19%
Lachnospira	-89.98%	-83.61%	-5.69%	-22.84%	-75.33%	-49.65%	-8.51%	4.50%	572.88%
Marvinbryantia	-76.74%	4085.56%	-60.66%	100.39%	-88.57%	-94.86%	Absent in T1	-57.86%	11.71%
Mobilitalea	1.59%	11.08%	178.81%	29.49%	-27.27%	-69.88%	-66.06%	-83.91%	-42.93%
Moryella	Absent in T1	Absent in T2	-78.32%	650.26%	298.83%	167.27%	Absent in T1	-86.97%	-82.82%
Oribacterium	-93.88%	-80.97%	1.90%	-68.36%	-77.36%	-75.45%	-97.11%	-86.70%	53.05%
Roseburia	-43.97%	5.06%	32.70%	-15.46%	-22.65%	13.43%	-56.27%	-80.45%	-49.72%
Ruminococcus gnavus group (Lachnospiraceae family)	-53.33%	121.75%	-61.19%	-8.73%	-35.32%	-27.51%	-35.52%	-46.01%	-66.01%
Syntrophococcus	Absent in T1	-5.57%	68.77%	-45.10%	-40.83%	24.15%	Absent in T1	Absent in T2	-89.14%
Tyzzerella 3	-91.28%	-62.84%	3.69%	-15.18%	200.79%	117.90%	52.54%	-82.67%	4.23%
Tyzzerella 4	-94.72%	Absent in T2	-30.46%	-4.31%	-29.65%	-0.29%	-92.15%	Absent in T2	-63.41%
Tyzzerella	-49.97%	-71.53%	-35.97%	-25.30%	-44.82%	-32.88%	-29.79%	-77.21%	-37.69%
U. m. of Lachnospiraceae family	-63.36%	-53.61%	11.46%	56.42%	1.47%	18.41%	-67.05%	-72.79%	29.02%
Peptococcus	-67.75%	93.91%	117.53%	12.20%	-54.71%	-30.25%	-51.47%	135.74%	308.30%
U. m. of Peptococcaceae family	-77.23%	-44.24%	22.63%	-29.67%	-53.62%	52.26%	-90.29%	-74.27%	-34.07%
Peptoclostridium	Absent in T1	Absent in T2	18.50%	Absent in both	Absent in both	Absent in T0	Absent in both	Absent in both	Absent in T0
Peptostreptococcus	Absent in both	Absent in both	Absent in T0	Absent in both	Absent in both	Absent in T0	Absent in both	Absent in both	Absent in T0
Romboutsia	-97.77%	-99.41%	-56.01%	-37.53%	18.99%	-48.21%	-92.63%	-97.60%	-17.66%
Terrisporobacter	-53.07%	Absent in T2	286.89%	27.74%	-8.08%	320.22%	22.61%	Absent in T2	62.32%
U. m. of Peptostreptococcaceae family	-97.14%	Absent in T2	-76.39%	61.04%	59.90%	-79.05%	Absent in T1	Absent in T2	-87.23%
Anaerofilum	-21.71%	-71.00%	4.63%	-53.87%	7.03%	98.95%	-17.58%	-36.78%	32.56%
Anaerotruncus	-67.16%	-50.06%	-32.60%	-10.18%	10.83%	12.55%	-61.56%	-67.68%	-15.73%
Butyricicoccus	-62.09%	-54.72%	-30.58%	-41.77%	-20.43%	-27.88%	13.16%	-52.71%	34.57%
Candidatus Soleaferrea	76.65%	64.05%	28.88%	-29.35%	-60.38%	-9.02%	-18.61%	-62.64%	-43.23%
Caproiciproducens	-50.58%	-52.21%	-7.67%	265.77%	262.24%	420.73%	-85.98%	-46.63%	0.93%
Eubacterium coprostanoligenes group (Ruminococcaceae family)	-7.78%	-60.76%	32.76%	-3.59%	26.91%	28.90%	-30.12%	-61.71%	10.47%
Faecalibacterium	-41.18%	587.95%	49.11%	7.44%	-57.68%	118.50%	654.72%	115.09%	1004.19%
Flavonifractor	-73.85%	-84.21%	-42.15%	28.12%	36.28%	-10.99%	-81.56%	-82.16%	-31.94%
Hydrogenoanaerobacterium	-51.58%	-73.72%	-25.25%	-17.98%	5.63%	-10.29%	-17.83%	-66.23%	-17.88%
Intestinimonas	-46.08%	-51.16%	-11.04%	2.62%	1.40%	13.55%	-38.41%	-60.91%	-4.54%
Oscillibacter	-47.09%	-25.04%	-7.19%	-24.35%	-7.26%	-13.57%	-44.83%	-54.63%	-0.96%
Oscillospira	-9.29%	-35.59%	-31.51%	-3.97%	23.07%	34.74%	14.88%	-46.08%	59.56%
Papillibacter	-62.08%	-8.77%	-16.44%	35.18%	96.76%	37.78%	-63.82%	-73.10%	-6.46%
Ruminiclostridium 1	Absent in T1	Absent in T2	-94.57%	-85.04%	118.93%	256.48%	Absent in T1	Absent in T2	86.13%
Ruminiclostridium 5	637.48%	206.23%	152.53%	-6.15%	-4.39%	59.07%	92.93%	38.35%	10.26%

Ruminiclostridium 6	-66.41%	-31.91%	-55.83%	-12.64%	25.51%	198.04%	-74.48%	-18.25%	8.30%
Ruminiclostridium 9	-38.90%	-40.18%	7.80%	-7.41%	-2.20%	-2.08%	-46.18%	-56.31%	-23.42%
Ruminiclostridium	-69.79%	-74.87%	-24.99%	-24.13%	-23.48%	-16.82%	-58.77%	-81.63%	27.61%
Ruminococcaceae NK4A214 group	-22.69%	-45.85%	-15.05%	-64.12%	-18.79%	-21.30%	-21.04%	-22.40%	8.72%
Ruminococcaceae UCG-002	-29.21%	-14.00%	14.44%	-20.69%	-40.47%	-28.98%	-48.79%	-63.99%	20.49%
Ruminococcaceae UCG-003	-93.79%	Absent in T2	-73.86%	341.82%	Absent in T2	201.91%	Absent in T0	Absent in both	Absent in T0
Ruminococcaceae UCG-004	-37.94%	-34.66%	-11.57%	-48.89%	-33.54%	12.85%	-44.45%	-27.34%	-31.80%
Ruminococcaceae UCG-005	20.16%	-22.59%	-57.95%	-61.00%	41.55%	-17.40%	610.91%	253.58%	34.35%
Ruminococcaceae UCG-007	46.24%	28.69%	199.53%	-30.41%	-9.83%	-7.60%	-6.55%	-43.88%	16.67%
Ruminococcaceae UCG-009	-77.37%	-59.75%	-37.25%	-11.41%	-9.68%	0.68%	-61.77%	-71.32%	11.53%
Ruminococcaceae UCG-010	-40.37%	-54.64%	-37.11%	6.81%	32.27%	8.80%	-26.31%	-54.02%	-10.07%
Ruminococcaceae UCG-013	-50.33%	-54.19%	25.57%	-56.46%	3.24%	-34.68%	-66.41%	-80.62%	1.83%
Ruminococcaceae UCG-014	-67.96%	-89.60%	-23.79%	-44.74%	-52.24%	-54.82%	-62.97%	48.51%	-61.09%
Ruminococcaceae V9D2013 group	-73.15%	-94.05%	11.70%	77.77%	27.07%	48.52%	Absent in T1	-83.81%	-12.11%
Ruminococcus 1	-77.24%	-67.23%	-35.12%	3.93%	83.45%	24.26%	-8.25%	-66.81%	-10.14%
Ruminococcus 2	150.66%	15.69%	90.30%	-66.51%	-12.90%	24.81%	-44.03%	-86.66%	-62.29%
Sporobacter	7.58%	-62.15%	28.16%	15.32%	25.86%	40.77%	-46.81%	-72.07%	-42.60%
Subdoligranulum	-64.82%	-97.51%	171.97%	122.80%	-60.94%	204.70%	629.65%	22.33%	1302.82%
U. m. of Ruminococcaceae family	0.68%	-29.68%	-1.87%	-10.63%	-12.01%	-8.72%	-40.59%	-50.27%	-24.37%
U. m. of Clostridiales order	-35.46%	-51.15%	-17.80%	-28.21%	-5.14%	-29.06%	-19.34%	-62.66%	-30.90%
Allobaculum	1495.27%	-7.53%	-78.81%	96.91%	163.75%	-82.23%	2336.68%	205.18%	-32.90%
Candidatus Stoquefichus	21848.77%	13543.55%	89.33%	-88.62%	12.01%	20.55%	31389.94%	8069.85%	178.72%
Catenibacterium	Absent in both	Absent in both	Absent in T0	Absent in both	Absent in both	Absent in T0	Absent in T0	Absent in both	Absent in T0
Dielma	249.94%	73.41%	923.78%	Absent in T1	Absent in T2	Absent in T3	-33.91%	Absent in T2	-90.05%
Erysipelatoclostridium	-70.09%	-67.94%	37.07%	-19.63%	1.09%	164.79%	-91.22%	Absent in T2	43.21%
Erysipelotrichaceae UCG-002	Absent in T1	Absent in T2	282.81%	Absent in both	Absent in both	Absent in T0	Absent in both	Absent in both	Absent in T0
Erysipelotrichaceae UCG-003	23.45%	70.20%	-28.28%	-76.13%	7.53%	-30.39%	904.94%	128.78%	-21.82%
Faecalibaculum	90.93%	-21.01%	26.35%	-33.94%	762.77%	451.60%	19.47%	-15.20%	83.16%
Holdemanella	Absent in T0	Absent in both	Absent in T0	Absent in both	Absent in T0	Absent in T0	Absent in T0	Absent in T0	Absent in T0
Turicibacter	-65.22%	-55.81%	-32.84%	-74.85%	-32.88%	-82.20%	-80.71%	-80.40%	-61.81%
U. m. of Erysipelotrichaceae family	359.74%	3.80%	18.77%	-24.96%	77.27%	-31.93%	139.96%	52.49%	-2.44%
Enterococcus	475.72%	542.50%	664.15%	-19.09%	6.74%	916.95%	921.53%	609.91%	769.27%
Lactobacillus	41.14%	-70.51%	-29.55%	17.63%	-70.04%	-46.13%	-5.36%	-36.48%	-43.72%
Lactococcus	-83.33%	-91.11%	-17.55%	-72.62%	-38.33%	117.27%	207.41%	Absent in T2	1363.90%
Streptococcus	17.93%	-66.96%	173.01%	-23.53%	-22.21%	60.53%	-53.18%	-73.57%	-24.58%
Acidaminococcus	93.63%	Absent in T2	199.17%	Absent in T0	Absent in both	Absent in T0	151.80%	Absent in T2	448.10%
Phascolarctobacterium	-34.67%	1998.83%	722.34%	1178.90%	21.28%	7637.55%	Absent in T0	Absent in T0	Absent in T0
Anaerovibrio	180.33%	184.41%	45.08%	-11.46%	11.16%	-12.04%	163.44%	18.78%	-1.58%
Dialister	44.63%	-90.06%	252.81%	Absent in T0	Absent in T0	Absent in T0	603.52%	30.20%	145.04%
Megamonas	-87.58%	Absent in T2	458.82%	-51.67%	-59.19%	298.75%	Absent in both	Absent in T0	Absent in T0
Megasphaera	40.79%	Absent in T2	-21.15%	Absent in both	Absent in both	Absent in T0	Absent in both	Absent in both	Absent in T0
Quinella	0.50%	83.34%	126.69%	-40.81%	-34.37%	-56.05%	-73.67%	-65.08%	-42.17%
Veillonella	-36.43%	-65.25%	62.01%	-27.99%	-36.73%	9.48%	-64.42%	-61.07%	34.04%
U. m. of Veillonellaceae family	14.71%	37.22%	60.99%	-15.85%	39.82%	47.34%	-7.27%	-13.50%	-6.37%
U. m. of Selenomonadales order	410.86%	76.89%	94.02%	-39.76%	87.31%	17.45%	109.68%	244.09%	34.39%
U. m. of Firmicutes phylum	4.19%	-19.57%	128.70%	-8.06%	30.75%	-12.37%	-26.33%	8.32%	6.99%
Cetobacterium	Absent in T1	Absent in T2	59.25%	Absent in both	Absent in both	Absent in both	Absent in both	Absent in both	Absent in T0
Fusobacterium	-77.99%	-81.24%	42.48%	-41.24%	-11.45%	57.48%	134.51%	90.26%	241.74%

Anaerobiospirillum	-71.10%	-42.56%	Absent in T3	-27.22%	-58.30%	-73.77%	-83.50%	-67.17%	-9.20%
Succinivibrio	-92.74%	-96.17%	603.76%	Absent in T0	Absent in T0	Absent in T0	31.43%	-32.93%	3380.54%
U. m. of Succinivibrionaceae family	-82.90%	-95.33%	388.52%	-19.95%	77.95%	163.78%	-87.44%	-48.69%	311.24%
U. m. of Aeromonadales order	Absent in T1	-0.93%	47.62%	-35.21%	-33.62%	24.11%	-89.10%	-78.13%	-57.94%
Parasutterella	-40.29%	29.49%	-53.48%	-57.84%	189.88%	26.61%	105.74%	162.55%	-3.15%
Sutterella	-36.55%	-79.68%	293.91%	Absent in T0	Absent in both	Absent in T0	2020.32%	587.79%	2795.85%
U. m. of CM1G08 family	Absent in T1	Absent in T2	Absent in T3	Absent in T1	-73.13%	111.71%	Absent in both	Absent in both	Absent in T0
U. m. of Burkholderiales order	-83.56%	52.38%	165.30%	-30.05%	854.62%	302.18%	-88.77%	-40.63%	244.17%
Helicobacter	-59.57%	-16.90%	0.29%	-39.81%	-10.17%	-33.64%	-36.34%	-19.49%	63.57%
Caulobacter	Absent in T0	Absent in both	Absent in T0	Absent in T1	-22.65%	Absent in T3	144.06%	56.63%	Absent in T3
U. m. of Cellvibrionales order	Absent in T1	Absent in T2	Absent in T3	Absent in T0	Absent in T0	Absent in T0	Absent in T1	-45.36%	Absent in T3
Bilophila	146.85%	Absent in T2	7.02%	-87.15%	-38.94%	45.70%	-26.31%	Absent in T2	-35.99%
Desulfovibrio	-11.41%	-23.43%	47.63%	0.55%	32.32%	32.13%	-62.42%	-62.38%	-19.35%
Citrobacter	-83.10%	Absent in T2	261.00%	Absent in T0	Absent in T0	Absent in T0	524.23%	Absent in T2	1216.23%
Enterobacter	-30.96%	-74.46%	227.27%	203.89%	-62.92%	798.60%	3320.48%	691.81%	6254.64%
Escherichia-Shigella	80.96%	-78.51%	308.58%	919.64%	710.66%	3454.00%	128.18%	48.52%	292.70%
Hafnia-Obesumbacterium	Absent in both	Absent in both	Absent in T0	Absent in T1	86.53%	294.65%	Absent in T0	Absent in both	Absent in T0
Klebsiella	-55.27%	-89.25%	360.88%	1178.90%	Absent in T2	3192.53%	356.39%	9.59%	1638.37%
Salmonella	Absent in T0	Absent in T0	Absent in T0	40.79%	331.95%	530.71%	Absent in T0	Absent in both	Absent in T0
Serratia	463.16%	Absent in T2	619.35%	Absent in T0	Absent in both	Absent in T0	Absent in T0	Absent in T0	Absent in T0
U. m. of Enterobacteriaceae family	90.66%	-81.32%	443.05%	Absent in T1	185.22%	975.95%	162.16%	Absent in T2	1218.61%
Haemophilus	Absent in T0	Absent in both	Absent in T0	Absent in T0	Absent in T0	Absent in T0	Absent in T0	Absent in both	Absent in both
Necropsobacter	-63.06%	145.35%	115.01%	28.34%	6.80%	90.01%	-86.55%	-3.12%	64.56%
Pasteurella	-60.30%	-19.36%	75.76%	-25.96%	-18.75%	-7.15%	-30.97%	-9.88%	-21.75%
U. m. of Pasteurellaceae family	Absent in both	Absent in T0	Absent in both	123.29%	37.55%	76.92%	Absent in both	Absent in T0	Absent in T0
Acinetobacter	40.79%	Absent in T2	690.12%	Absent in both	Absent in T0	Absent in T0	Absent in T0	Absent in both	Absent in T0
Enhydrobacter	Absent in T0	Absent in both	Absent in both	Absent in T1	Absent in T2	126.35%	Absent in T0	Absent in T0	Absent in both
Paracoccus	Absent in T0	Absent in both	Absent in T0	Absent in T0	Absent in both	Absent in T0	Absent in T0	Absent in T0	Absent in both
U. m. of Rhodospirillaceae family	-46.94%	129.49%	74.97%	-26.17%	209.89%	1.10%	204.65%	281.53%	200.26%
Vibrio	-44.49%	Absent in T2	-31.18%	Absent in both	Absent in both	Absent in T0	Absent in both	Absent in both	Absent in T0
Stenotrophomonas	81.11%	107.96%	-87.66%	155.78%	271.27%	-46.42%	276.40%	472.03%	712.25%
U. m. of Proteobacteria phylum	-42.98%	-18.26%	-41.16%	3.11%	-28.09%	-8.39%	-72.60%	-54.79%	-0.57%
U. m. of Saccharibacteria phylum	1.78%	-82.58%	39.85%	-17.16%	-0.94%	-36.20%	-57.05%	-86.64%	-48.67%
Treponema 2	29.17%	705.53%	180.45%	3.13%	557.56%	439.06%	-47.89%	1020.66%	197.34%
Anaeroplasma	Absent in T1	Absent in T2	-58.52%	-32.14%	66.84%	-38.90%	-95.02%	Absent in T2	-93.28%
U. m. of Mollicutes RF9 order	-76.57%	-86.31%	34.40%	-40.16%	-15.10%	-59.92%	-64.97%	-83.46%	-60.41%
U. m. of Mycoplasmataceae family	206.42%	393.69%	222.58%	21.02%	940.05%	328.65%	-25.99%	134.35%	20.77%
U. m. of Opitutae vadinHA64 order	Absent in T1	-49.24%	-20.39%	-20.76%	241.27%	-13.47%	Absent in both	Absent in T0	Absent in both
U. m. of Verrucomicrobia phylum	-89.87%	-15.57%	-74.30%	12.01%	34.26%	-78.47%	7.08%	-41.25%	129.05%
Akkermansia	138.74%	-60.59%	508.48%	-38.47%	-73.32%	72.37%	604.27%	72.65%	292.85%
U. m. of Verrucomicrobiaceae family	Absent in T0	Absent in both	Absent in T0	Absent in both	Absent in T0	Absent in both	Absent in T0	Absent in both	Absent in T0
U. m. of Bacteria kingdom	-30.99%	113.38%	-10.26%	-51.54%	123.40%	-75.60%	-62.04%	-22.61%	-82.36%
Others	-66.65%	118.94%	55.32%	-81.52%	183.01%	-92.25%	-56.82%	-32.54%	-87.31%

Taxonomy	Trend (effect of treatment respect to T0 controls) at genus level								
	CG			Breve2L			CG+Breve2L		
	T0-T1	T0-T2	T0-T3	T0-T1	T0-T2	T0-T3	T0-T1	T0-T2	T0-T3
U. m. of Euryarchaeota phylum	-	-	-		+		-	-	-
Actinomyces	+	-	+	-	-	-	-	-	-
Bifidobacterium	+	-	+	+	+	+	+	-	+
Gardnerella	+	-	+				+	-	+
Adlercreutzia	+	-	-	+	+	+	+	-	-
Atopobium	+	-	+	-	-	-	-	-	+
Collinsella	-	+	+	+	+	+	+	+	+
Coriobacteriaceae UCG-002	+	+	-	-	+	-	+	+	-
Enterorhabdus	-	-	+	-	+	+	-	-	-
Gordonibacter	+	-	+	-	+	-	+	+	+
Parvibacter	-	-	+	+	+	+	-	-	+
Senegalimassilia		+	+		+				+
U. m. of Coriobacteriaceae family	+	-	+	-	-	-	-	-	-
Corynebacterium	-	+	-			+	+	-	+
Microbacterium	+	-	+			+	+		+
U. m. of Microbacteriaceae family	+	-	-				+		
Rothia	-	-	+			+	+		+
U. m. of Micrococcaceae family	+	-	+	-	-	+	+	+	+
Propionibacterium	-	-	-	+	+	+	+	+	+
U. m. of Actinobacteria phylum	-	-	-		+	+	-	-	-
Bacteroides	-	-	-	-	-	-	-	-	+
U. m. of Bacteroidales BS11 gut group family	-	-	+		+	+			+
U. m. of Bacteroidales RF16 group family	-	-	+	-	-	-	-	+	-
U. m. of Bacteroidales S24-7 group family	+	-	-	-	+	-	+	+	-
Barnesiella	-	-	+	-	-	+	+	-	+
Butyricimonas	-	+	-	+	+	-	+	+	+
Coprobacter			+			+			+
Odoribacter	-	-	-	+	+	+	-	-	+
Parabacteroides	+	+	-	-	+	+	+	+	-
U. m. of Porphyromonadaceae family	-	-	+	+	+	+	-	+	+
Alloprevotella	-	-	+	-	-	+	-	+	+
Paraprevotella	-	+	-	-	-	-	-	-	+
Prevotella 1	+	+	-	+	-	-	+	+	-
Prevotella 2	+	+	-	+	+	+	+	+	-
Prevotella 7	-	+	-	-	-	-	+	+	-
Prevotella 9	+	-	+	+	+	+	+	+	+
Prevotellaceae Ga6A1 group			+			+			+
Prevotellaceae NK3B31 group	+	+	+	+	+	+	+	+	+
Prevotellaceae UCG-001	-	+	+	+	+	+	-	+	+
Prevotellaceae UCG-003	-	+	+	+	+	-	-	+	-
Prevotellaceae UCG-004	-	-	-	+	+	-	-	+	-
Prevotella	-	+	-	+	+	+	-	+	-
U. m. of Prevotellaceae family	+	+	-	-	-	+	+	+	-
Alistipes	-	-	+	-	+	+	-	-	-

Rikenellaceae RC9 gut group	-	+	+	-	+	+	-	-	+
Rikenella	-	-	-	-	+	+	-	-	-
U. m. of Rikenellaceae family	-	-	-	+	+	-	-	-	-
U. m. of Bacteroidales order	-	-	+	-	-	-	-	-	-
U. m. of Cytophagales order	-	-	+	+	+	+	-	-	-
Cloacibacterium	-	+	-	-	-	-	-	-	-
Flavobacterium	+	+	+				+	-	-
U. m. of Bacteroidetes phylum	-	-	-	+	+	+	-	-	+
U. m. of AKIW781 family	-	-	-						
U. m. of Cyanobacteria phylum	+	-	-	+	+	+	+	+	+
U. m. of Gastranaerophilales order	-	+	+	+	+	+	+	+	+
Mucispirillum	-	+	+	-	-	+	-	-	+
Anoxybacillus	+	-	+				+		
Bacillus	-	-	-	-	+	-	+	-	-
Terribacillus	-	-	-	-	+	-	-	-	-
Gemella	-	-	+	-	-	-	-	-	-
Brochothrix	-	-	+			+			+
Listeria	-	-	+			+			+
Staphylococcus	+	+	+	+	-	+	+	+	+
Catabacter	+	-	+	-	-	+	-	-	+
Christensenellaceae R-7 group	-	-	-	-	+	+	-	-	-
U. m. of Christensenellaceae family	-	-	-	-	+	+	-	-	+
Clostridium sensu stricto 1	-	-	+	+	-	+	-	-	+
U. m. of Clostridiales vadinBB60 group family	-	+	+	-	+	+	-	-	+
Defluviitaleaceae UCG-011	+	+	+	+	+	+	-	+	+
Anaerofustis	-	-	-	+	+	-	-	-	-
Anaerovorax	-	-	+	-	-	-	-	-	-
Eubacterium nodatum group (Clostridiales order, Family XIII family)	-	-	-	-	-	-	-	-	-
Family XIII AD3011 group (Clostridiales order)	-	-	+	-	+	+	-	-	-
Family XIII UCG-001 (Clostridiales order)	-	-	+	+	+	+	-	-	-
Mogibacterium	-	-	-	-	+	-	-	-	-
U. m. of Family XIII family (Clostridiales order)	+	-	+	+	+	-	-	-	-
Acetatifactor	-	-	-	+	-	+	-	-	+
Anaerostipes	+	-	-	+	+	+	+	-	+
Blautia	-	-	-	-	-	-	+	+	+
Butyrivibrio 2	-	-	+	+		+			+
Butyrivibrio	-	-	+	-	-	-	-	-	-
Coprococcus 2	-	-	-	+	-	+	-	-	+
Coprococcus 3	-	-	+	-	+	-	-	-	-
Dorea	-	-	-	-	-	-	-	-	-
Eisenbergiella	-	+	-	+	-	-	-	-	-
Eubacterium eligens group (Lachnospiraceae family)	-	+	+	-	-	+	+	-	+
Eubacterium fissicatena group (Lachnospiraceae family)	-	-	+	-	-	-	-	-	+
Eubacterium hallii group (Lachnospiraceae family)	-	-	+	+	-	+	-	-	+
Eubacterium ruminantium group (Lachnospiraceae family)	-	-	+	-	-	+	+	-	+
Eubacterium uniforme	+	+	-				+	+	
Eubacterium ventriosum group (Lachnospiraceae family)	+	+	-	+	+	+	+	+	+
Fusicatenibacter	-	+	-	-	-	-	-	-	+

Howardella	-	-	-	+	+	+	-	-	-
Hungatella	+	-	+	-	+	-	+		+
Lachnoanaerobaculum	-	+	+	-	+	+	-	+	+
Lachnoclostridium 10	-	-	-	-	-	+	-	-	-
Lachnoclostridium	-	-	+	+	-	+	+	-	+
Lachnospiraceae FCS020 group	-	-	-	-	-	-	-	-	-
Lachnospiraceae ND3007 group	-	-	-	-	-	-	+	-	-
Lachnospiraceae NK3A20 group	-	-	-	-	+	-	-	-	-
Lachnospiraceae NK4A136 group	-	-	+	-	+	+	-	-	+
Lachnospiraceae NK4B4 group	-	-	-	-	+	+	-	-	-
Lachnospiraceae UCG-001	-	+	-	+	+	-	+	+	+
Lachnospiraceae UCG-006	-	+	-	+	+	-	-	-	+
Lachnospiraceae UCG-010	-	-	+	-	-	-	-	-	+
Lachnospiraceae XPB1014 group	-	-	+	+	-	-	-	-	-
Lachnospira	-	-	-	-	-	-	-	+	+
Marvinbryantia	-	+	-	+	-	-	-	-	+
Mobilitalea	+	+	+	+	-	-	-	-	-
Moryella	-	-	-	+	+	+	-	-	-
Oribacterium	-	-	+	-	-	-	-	-	+
Roseburia	-	+	+	-	-	+	-	-	-
Ruminococcus gnavus group (Lachnospiraceae family)	-	+	-	-	-	-	-	-	-
Syntrophococcus	-	-	+	-	-	+	-	-	-
Tyzzerella 3	-	-	+	-	+	+	+	-	+
Tyzzerella 4	-	-	-	-	-	-	-	-	-
Tyzzerella	-	-	-	-	-	-	-	-	-
U. m. of Lachnospiraceae family	-	-	+	+	+	+	-	-	+
Peptococcus	-	+	+	+	-	-	-	+	+
U. m. of Peptococcaceae family	-	-	+	-	-	+	-	-	-
Peptoclostridium	-	-	+			+			+
Peptostreptococcus			+			+			+
Romboutsia	-	-	-	-	+	-	-	-	-
Terrisporobacter	-	-	+	+	-	+	+	-	+
U. m. of Peptostreptococcaceae family	-	-	-	+	+	-	-	-	-
Anaerofilum	-	-	+	-	+	+	-	-	+
Anaerotruncus	-	-	-	-	+	+	-	-	-
Butyricicoccus	-	-	-	-	-	-	+	-	+
Candidatus Soleaferrea	+	+	+	-	-	-	-	-	-
Caproiciproducens	-	-	-	+	+	+	-	-	+
Eubacterium coprostanoligenes group (Ruminococcaceae family)	-	-	+	-	+	+	-	-	+
Faecalibacterium	-	+	+	+	-	+	+	+	+
Flavonifractor	-	-	-	+	+	-	-	-	-
Hydrogenoanaerobacterium	-	-	-	-	+	-	-	-	-
Intestinimonas	-	-	-	+	+	+	-	-	-
Oscillibacter	-	-	-	-	-	-	-	-	-
Oscillospira	-	-	-	-	+	+	+	-	+
Papillibacter	-	-	-	+	+	+	-	-	-
Ruminiclostridium 1	-	-	-	-	+	+	-	-	+
Ruminiclostridium 5	+	+	+	-	-	+	+	+	+

Ruminiclostridium 6	-	-	-	-	+	+	-	-	+
Ruminiclostridium 9	-	-	+	-	-	-	-	-	-
Ruminiclostridium	-	-	-	-	-	-	-	-	+
Ruminococcaceae NK4A214 group	-	-	-	-	-	-	-	-	+
Ruminococcaceae UCG-002	-	-	+	-	-	-	-	-	+
Ruminococcaceae UCG-003	-	-	-	+	-	+	+		+
Ruminococcaceae UCG-004	-	-	-	-	-	+	-	-	-
Ruminococcaceae UCG-005	+	-	-	-	+	-	+	+	+
Ruminococcaceae UCG-007	+	+	+	-	-	-	-	-	+
Ruminococcaceae UCG-009	-	-	-	-	-	+	-	-	+
Ruminococcaceae UCG-010	-	-	-	+	+	+	-	-	-
Ruminococcaceae UCG-013	-	-	+	-	+	-	-	-	+
Ruminococcaceae UCG-014	-	-	-	-	-	-	-	+	-
Ruminococcaceae V9D2013 group	-	-	+	+	+	+	-	-	-
Ruminococcus 1	-	-	-	+	+	+	-	-	-
Ruminococcus 2	+	+	+	-	-	+	-	-	-
Sporobacter	+	-	+	+	+	+	-	-	-
Subdoligranulum	-	-	+	+	-	+	+	+	+
U. m. of Ruminococcaceae family	+	-	-	-	-	-	-	-	-
U. m. of Clostridiales order	-	-	-	-	-	-	-	-	-
Allobaculum	+	-	-	+	+	-	+	+	-
Candidatus Stoquefichus	+	+	+	-	+	+	+	+	+
Catenibacterium			+			+	+		+
Dielma	+	+	+	-	-	-	-	-	-
Erysipelatoclostridium	-	-	+	-	+	+	-	-	+
Erysipelotrichaceae UCG-002	-	-	+			+			+
Erysipelotrichaceae UCG-003	+	+	-	-	+	-	+	+	-
Faecalibaculum	+	-	+	-	+	+	+	-	+
Holdemanella	+		+		+	+	+	+	+
Turicibacter	-	-	-	-	-	-	-	-	-
U. m. of Erysipelotrichaceae family	+	+	+	-	+	-	+	+	-
Enterococcus	+	+	+	-	+	+	+	+	+
Lactobacillus	+	-	-	+	-	-	-	-	-
Lactococcus	-	-	-	-	-	+	+	-	+
Streptococcus	+	-	+	-	-	+	-	-	-
Acidaminococcus	+	-	+	+		+	+	-	+
Phascolarctobacterium	-	+	+	+	+	+	+	+	+
Anaerovibrio	+	+	+	-	+	-	+	+	-
Dialister	+	-	+	+	+	+	+	+	+
Megamonas	-	-	+	-	-	+		+	+
Megasphaera	+	-	-			+			+
Quinella	+	+	+	-	-	-	-	-	-
Veillonella	-	-	+	-	-	+	-	-	+
U. m. of Veillonellaceae family	+	+	+	-	+	+	-	-	-
U. m. of Selenomonadales order	+	+	+	-	+	+	+	+	+
U. m. of Firmicutes phylum	+	-	+	-	+	-	-	+	+
Cetobacterium	-	-	+						+
Fusobacterium	-	-	+	-	-	+	+	+	+

Anaerobiospirillum	-	-	-	-	-	-	-	-	-
Succinivibrio	-	-	+	+	+	+	+	-	+
U. m. of Succinivibrionaceae family	-	-	+	-	+	+	-	-	+
U. m. of Aeromonadales order	-	-	+	-	-	+	-	-	-
Parasutterella	-	+	-	-	+	+	+	+	-
Sutterella	-	-	+	+		+	+	+	+
U. m. of CM1G08 family	-	-	-	-	-	+			+
U. m. of Burkholderiales order	-	+	+	-	+	+	-	-	+
Helicobacter	-	-	+	-	-	-	-	-	+
Caulobacter	+		+	-	-	-	+	+	-
U. m. of Cellvibrionales order	-	-	-	+	+	+	-	-	-
Bilophila	+	-	+	-	-	+	-	-	-
Desulfovibrio	-	-	+	+	+	+	-	-	-
Citrobacter	-	-	+	+	+	+	+	-	+
Enterobacter	-	-	+	+	-	+	+	+	+
Escherichia-Shigella	+	-	+	+	+	+	+	+	+
Hafnia-Obesumbacterium			+	-	+	+	+		+
Klebsiella	-	-	+	+	-	+	+	+	+
Salmonella	+	+	+	+	+	+	+		+
Serratia	+	-	+	+		+	+	+	+
U. m. of Enterobacteriaceae family	+	-	+	-	+	+	+	-	+
Haemophilus	+		+	+	+	+	+		
Necropsobacter	-	+	+	+	+	+	-	-	+
Pasteurella	-	-	+	-	-	-	-	-	-
U. m. of Pasteurellaceae family		+		+	+	+		+	+
Acinetobacter	+	-	+		+	+	+		+
Enhydrobacter	+			-	-	+	+	+	
Paracoccus	+		+	+		+	+	+	
U. m. of Rhodospirillaceae family	-	+	+	-	+	+	+	+	+
Vibrio	-	-	-			+			+
Stenotrophomonas	+	+	-	+	+	-	+	+	+
U. m. of Proteobacteria phylum	-	-	-	+	-	-	-	-	-
U. m. of Saccharibacteria phylum	+	-	+	-	-	-	-	-	-
Treponema 2	+	+	+	+	+	+	-	+	+
Anaeroplasma	-	-	-	-	+	-	-	-	-
U. m. of Mollicutes RF9 order	-	-	+	-	-	-	-	-	-
U. m. of Mycoplasmataceae family	+	+	+	+	+	+	-	+	+
U. m. of Opitutae vadinHA64 order	-	-	-	-	+	-		+	
U. m. of Verrucomicrobia phylum	-	-	-	+	+	-	+	-	+
Akkermansia	+	-	+	-	-	+	+	+	+
U. m. of Verrucomicrobiaceae family	+		+		+		+		+
U. m. of Bacteria kingdom	-	+	-	-	+	-	-	-	-
Others	-	+	+	-	+	-	-	-	-

Taxonomy	p-value at genus level								
	CG			Breve2L			CG+Breve2L		
	T_1	T_2	T_3	T_1	T_2	T_3	T_1	T_2	T_3
U. m. of Euryarchaeota phylum		0.035	0.035		0.035				0.002
Actinomyces								0.045	0.033
Bifidobacterium			0.012			0.011			
Gardnerella		0.035						0.035	
Adlercreutzia									
Atopobium		0.026							
Collinsella					0.004	0.011	0.045		0.002
Coriobacteriaceae UCG-002	0.010	0.014							
Enterorhabdus							0.014	0.009	
Gordonibacter						0.003			
Parvibacter	0.000	0.010		0.035	0.003	0.000	0.035	0.035	
Senegalimassilia		0.035	0.001		0.038				0.019
U. m. of Coriobacteriaceae family				0.018			0.005	0.000	0.000
Corynebacterium						0.001	0.031	0.035	
Microbacterium		0.035				0.035	0.035		0.000
U. m. of Microbacteriaceae family		0.035					0.004		
Rothia	0.000	0.000	0.029			0.016	0.020		0.001
U. m. of Micrococcaceae family			0.044						
Propionibacterium					0.045		0.011		
U. m. of Actinobacteria phylum	0.000				0.003	0.035	0.009	0.011	
Bacteroides	0.034				0.049	0.041		0.008	
U. m. of Bacteroidales BS11 gut group family	0.035	0.035			0.035	0.034			0.000
U. m. of Bacteroidales RF16 group family	0.033								
U. m. of Bacteroidales S24-7 group family						0.045			
Barnesiella		0.003			0.004			0.002	0.001
Butyricimonas								0.029	0.035
Coprobacter			0.036			0.035			0.001
Odoribacter									0.042
Parabacteroides			0.019				0.003		
U. m. of Porphyromonadaceae family			0.002		0.015				0.012
Alloprevotella									
Paraprevotella		0.048		0.019	0.007				
Prevotella 1									0.010
Prevotella 2		0.001							0.049
Prevotella 7			0.029						
Prevotella 9		0.012	0.007	0.046	0.038	0.041	0.007	0.013	0.000
Prevotellaceae Ga6A1 group			0.000			0.035			0.035
Prevotellaceae NK3B31 group			0.003	0.013		0.020			0.001
Prevotellaceae UCG-001			0.023			0.018			
Prevotellaceae UCG-003									
Prevotellaceae UCG-004	0.000	0.010	0.005			0.001	0.035		
Prevotella		0.036	0.010				0.026		0.038
U. m. of Prevotellaceae family				0.025					0.003
Alistipes					0.036				

Rikenellaceae RC9 gut group					0.002				
Rikenella	0.000	0.000	0.001		0.022	0.035	0.000	0.000	0.000
U. m. of Rikenellaceae family	0.001	0.000	0.026				0.010		
U. m. of Bacteroidales order									
U. m. of Cytophagales order	0.006	0.039		0.047	0.017		0.028	0.028	
Cloacibacterium			0.030	0.035	0.035	0.035			0.035
Flavobacterium	0.000	0.035	0.004				0.021		0.035
U. m. of Bacteroidetes phylum									
U. m. of AKIW781 family	0.035	0.035	0.035						
U. m. of Cyanobacteria phylum							0.036	0.016	
U. m. of Gastranaerophilales order		0.034	0.000		0.048	0.028	0.037	0.037	
Mucispirillum	0.030			0.008					
Anoxybacillus		0.035					0.039		
Bacillus							0.036	0.000	0.000
Terribacillus	0.009	0.001	0.001	0.004		0.005		0.035	
Gemella	0.005	0.000		0.036				0.039	
Brochothrix	0.035	0.035				0.001			0.035
Listeria	0.035	0.035				0.035			0.000
Staphylococcus	0.005		0.042				0.001		
Catabacter		0.035					0.001	0.001	
Christensenellaceae R-7 group		0.029	0.011				0.044		
U. m. of Christensenellaceae family							0.013	0.033	
Clostridium sensu stricto 1	0.003	0.001			0.037	0.028			
U. m. of Clostridiales vadinBB60 group family		0.016							
Defluviitaleaceae UCG-011	0.000	0.001	0.020						
Anaerofustis	0.007	0.007					0.003	0.003	
Anaerovorax			0.007			0.047	0.009	0.005	
Eubacterium nodatum group (Clostridiales order, Family XIII family)	0.012						0.000	0.000	0.000
Family XIII AD3011 group (Clostridiales order)									
Family XIII UCG-001 (Clostridiales order)						0.016			0.020
Mogibacterium				0.009	0.008	0.001			0.003
U. m. of Family XIII family (Clostridiales order)									
Acetatifactor	0.000	0.000					0.000	0.002	0.001
Anaerostipes		0.010							0.030
Blautia	0.039		0.034				0.000		
Butyrivibrio 2	0.006	0.006	0.040	0.035		0.000			0.000
Butyrivibrio	0.032	0.032	0.042				0.042	0.036	
Coprococcus 2		0.046				0.024			
Coprococcus 3	0.006	0.021					0.024		
Dorea	0.000	0.000	0.000	0.039	0.034		0.028	0.024	0.039
Eisenbergiella			0.006	0.044			0.034	0.002	0.003
Eubacterium eligens group (Lachnospiraceae family)					0.013		0.001		0.006
Eubacterium fissicatena group (Lachnospiraceae family)									
Eubacterium hallii group (Lachnospiraceae family)	0.018						0.002	0.005	
Eubacterium ruminantium group (Lachnospiraceae family)		0.015		0.000			0.009		
Eubacterium uniforme			0.035				0.035	0.012	
Eubacterium ventriosum group (Lachnospiraceae family)	0.002	0.016					0.001	0.008	
Fusicatenibacter	0.006	0.039	0.006				0.002	0.000	

Howardella	0.035	0.035	0.035	0.035	0.035	0.035	0.000	0.000	
Hungatella		0.035					0.035		0.000
Lachnoanaerobaculum					0.037	0.031		0.008	0.019
Lachnoclostridium 10		0.004	0.005						0.000
Lachnoclostridium		0.008				0.045	0.037		0.002
Lachnospiraceae FCS020 group		0.007							
Lachnospiraceae ND3007 group		0.004			0.041			0.001	
Lachnospiraceae NK3A20 group		0.037		0.017		0.039		0.011	0.041
Lachnospiraceae NK4A136 group	0.004	0.008				0.002	0.000	0.002	
Lachnospiraceae NK4B4 group	0.036	0.036	0.048			0.026	0.033	0.018	
Lachnospiraceae UCG-001	0.028				0.025				
Lachnospiraceae UCG-006								0.002	
Lachnospiraceae UCG-010									
Lachnospiraceae XPB1014 group					0.032	0.015	0.004	0.008	
Lachnospira	0.001	0.012							0.002
Marvinbryantia		0.037			0.014	0.007	0.020		
Mobilitalea								0.023	
Moryella	0.000	0.000	0.002		0.016		0.002		
Oribacterium	0.032				0.039		0.004	0.037	
Roseburia								0.008	0.017
Ruminococcus gnavus group (Lachnospiraceae family)									0.001
Syntrophococcus	0.035						0.027	0.027	
Tyzzerella 3	0.009				0.035			0.012	
Tyzzerella 4	0.021	0.008					0.007	0.002	
Tyzzerella									0.024
U. m. of Lachnospiraceae family	0.030	0.021		0.030		0.038	0.010	0.009	
Peptococcus	0.026								
U. m. of Peptococcaceae family	0.027						0.000	0.001	
Peptoclostridium	0.035	0.035				0.035			0.010
Peptostreptococcus			0.001			0.000			0.002
Romboutsia	0.000	0.000	0.001				0.046	0.041	
Terrisporobacter		0.035				0.014		0.001	
U. m. of Peptostreptococcaceae family	0.001	0.001	0.012			0.036	0.035	0.035	
Anaerofilum		0.026		0.043					
Anaerotruncus	0.001								
Butyricicoccus	0.016					0.031			
Candidatus Soleaferrea									
Caproiciproducens				0.007		0.011	0.014		
Eubacterium coprostanoligenes group (Ruminococcaceae family)									
Faecalibacterium	0.032						0.001		0.003
Flavonifractor							0.009	0.005	
Hydrogenoanaerobacterium	0.027	0.012							
Intestinimonas									
Oscillibacter							0.019		0.019
Oscillospira									
Papillibacter					0.013				
Ruminiclostridium 1	0.000	0.000	0.001	0.028		0.009	0.004	0.004	
Ruminiclostridium 5	0.001		0.027			0.000		0.024	

Ruminiclostridium 6	0.018					0.001			
Ruminiclostridium 9					0.011		0.037		
Ruminiclostridium		0.038						0.020	
Ruminococcaceae NK4A214 group				0.005	0.027				
Ruminococcaceae UCG-002					0.016				
Ruminococcaceae UCG-003	0.047	0.030			0.035	0.040	0.000		0.000
Ruminococcaceae UCG-004									
Ruminococcaceae UCG-005				0.013		0.010	0.005	0.007	
Ruminococcaceae UCG-007							0.006		
Ruminococcaceae UCG-009	0.000		0.001				0.044	0.025	
Ruminococcaceae UCG-010		0.048	0.002		0.033			0.028	0.028
Ruminococcaceae UCG-013			0.021	0.028		0.026	0.006	0.010	
Ruminococcaceae UCG-014	0.017	0.000							
Ruminococcaceae V9D2013 group		0.021					0.000		
Ruminococcus 1	0.007	0.045						0.034	
Ruminococcus 2	0.012							0.037	
Sporobacter	0.017							0.011	
Subdoligranulum	0.010	0.001							0.003
U. m. of Ruminococcaceae family					0.027				
U. m. of Clostridiales order									
Allobaculum	0.002		0.035		0.029	0.007	0.038	0.045	
Candidatus Stoquefichus	0.003	0.023					0.035	0.004	
Catenibacterium			0.001			0.035	0.035		0.015
Dielma				0.000	0.000	0.000		0.037	
Erysipelatoclostridium						0.020	0.041	0.004	
Erysipelotrichaceae UCG-002	0.035	0.035				0.000			0.000
Erysipelotrichaceae UCG-003				0.004			0.016		
Faecalibaculum					0.001				
Holdemanella	0.035		0.001		0.035	0.035	0.035	0.035	0.001
Turicibacter	0.019								
U. m. of Erysipelotrichaceae family	0.009				0.025				
Enterococcus	0.009	0.011				0.008	0.008	0.022	
Lactobacillus					0.000				
Lactococcus		0.046		0.045		0.033		0.035	0.002
Streptococcus		0.001	0.022			0.049	0.036	0.026	
Acidaminococcus		0.035		0.035		0.035		0.035	0.009
Phascolarctobacterium		0.041	0.005			0.004	0.000	0.002	0.002
Anaerovibrio		0.004							
Dialister		0.030	0.031	0.035	0.035	0.005	0.008		
Megamonas	0.000	0.000	0.008					0.035	0.001
Megasphaera		0.035				0.003			0.000
Quinella		0.005	0.000			0.020	0.003	0.048	
Veillonella									
U. m. of Veillonellaceae family					0.040				
U. m. of Selenomonadales order	0.012						0.004	0.028	
U. m. of Firmicutes phylum									
Cetobacterium	0.035	0.035							0.000
Fusobacterium							0.010		

Anaerobiospirillum	0.043		0.002			0.022	0.001		0.001	0.044	
Succinivibrio	0.046	0.042	0.008		0.035	0.035	0.040				0.001
U. m. of Succinivibrionaceae family		0.038	0.016				0.050		0.008		
U. m. of Aeromonadales order	0.000								0.022	0.041	
Parasutterella									0.037		
Sutterella			0.026		0.035		0.001		0.000		0.007
U. m. of CM1G08 family	0.035	0.035	0.035		0.001	0.024					0.007
U. m. of Burkholderiales order	0.024					0.022					
Helicobacter	0.019										
Caulobacter	0.035		0.020		0.002		0.002				0.035
U. m. of Cellvibrionales order	0.035	0.035	0.035		0.002	0.035	0.035		0.035		0.035
Bilophila		0.005			0.019					0.002	
Desulfovibrio	0.000										
Citrobacter	0.050	0.016	0.031		0.035	0.035	0.000		0.020	0.035	0.009
Enterobacter		0.010	0.024			0.019	0.008		0.000	0.031	0.000
Escherichia-Shigella		0.010	0.006		0.035	0.008	0.006				
Hafnia-Obesumbacterium			0.001		0.035		0.025		0.035		0.003
Klebsiella						0.035	0.000		0.047		0.002
Salmonella	0.035	0.035	0.000				0.004		0.035		0.000
Serratia		0.035	0.010		0.035		0.003		0.002	0.035	0.006
U. m. of Enterobacteriaceae family					0.035		0.002			0.035	0.044
Haemophilus	0.001		0.036		0.035	0.035	0.035		0.013		
Necropsobacter		0.033							0.009		
Pasteurella										0.027	
U. m. of Pasteurellaceae family		0.035								0.035	0.035
Acinetobacter		0.035	0.007			0.035	0.001		0.000		0.001
Enhydrobacter	0.035				0.035	0.035			0.021	0.035	
Paracoccus	0.003		0.035		0.035		0.035		0.035	0.038	
U. m. of Rhodospirillaceae family					0.035				0.014	0.001	0.000
Vibrio		0.001					0.000				0.035
Stenotrophomonas									0.013	0.010	
U. m. of Proteobacteria phylum											
U. m. of Saccharibacteria phylum	0.047	0.027	0.043		0.004		0.003				
Treponema 2		0.011	0.007			0.004	0.032			0.018	
Anaeroplasma	0.030	0.030							0.012	0.006	0.013
U. m. of Mollicutes RF9 order	0.025	0.033									
U. m. of Mycoplasmataceae family	0.050					0.013	0.025			0.033	
U. m. of Opitutae vadinHA64 order	0.001					0.017				0.035	
U. m. of Verrucomicrobia phylum	0.001		0.002								
Akkermansia	0.049		0.008						0.016		
U. m. of Verrucomicrobiaceae family	0.002		0.002			0.035			0.024		0.035
U. m. of Bacteria kingdom		0.029			0.016	0.021	0.023		0.014	0.005	0.000
Others	0.003									0.008	0.001

Supplementary File 2

	Bifidobacterial composition (%)											
	CG	CG	CG	CG	Breve2L	Breve2L	Breve2L	Breve2L	CG+Breve2L	CG+Breve2L	CG+Breve2L	CG+Breve2L
	T_0	T_1	T_2	T_3	T_0	T_1	T_2	T_3	T_0	T_1	T_2	T_3
	Average	Average	Average	Average	Average	Average	Average	Average	Average	Average	Average	Average
**Taxonomy**												
B. adolescentis biotype A	1.39%	1.79%	1.21%	1.88%	0.99%	1.38%	0.82%	2.12%	1.46%	1.58%	1.08%	3.86%
B. adolescentis biotype B	2.34%	2.89%	2.08%	3.11%	0.91%	1.59%	1.80%	4.85%	4.30%	3.35%	2.52%	3.02%
B. adolescentis biotype C	3.22%	2.44%	2.98%	2.26%	2.63%	1.47%	1.60%	2.32%	2.37%	3.11%	1.60%	2.08%
B. animalis subsp *animalis*	0.99%	0.37%	0.54%	1.15%	0.09%	0.01%	4.28%	7.94%	0.34%	0.20%	0.07%	1.78%
B. animalis subsp *lactis*	3.36%	4.54%	3.97%	0.98%	4.96%	1.75%	2.62%	1.11%	5.13%	6.12%	2.03%	1.08%
B. bifidum	2.76%	1.26%	0.93%	1.58%	1.39%	0.46%	0.58%	1.03%	2.05%	0.94%	0.52%	0.19%
B. breve	18.32%	23.25%	20.03%	8.49%	21.51%	51.29%	44.44%	23.20%	28.83%	35.45%	39.23%	15.29%
B. dentium	0.02%	1.40%	0.69%	0.16%	0.43%	0.15%	0.29%	0.57%	0.81%	0.40%	0.33%	0.34%
B. gallinarum	1.90%	0.20%	0.07%	0.09%	0.22%	2.00%	0.05%	0.07%	0.04%	0.04%	0.05%	0.25%
B. kashiwanohense	0.01%	0.58%	0.13%	0.01%	0.06%	1.22%	0.11%	0.04%	0.17%	0.15%	0.08%	0.17%
B. longum subsp *longum*	5.97%	4.54%	4.61%	0.91%	12.59%	3.54%	2.70%	3.85%	5.43%	4.29%	3.75%	1.07%
B. longum subsp *suis*	1.69%	1.12%	1.37%	1.57%	1.63%	1.47%	0.70%	1.22%	1.49%	1.26%	0.69%	0.54%
B. magnum	0.73%	1.89%	1.39%	3.19%	1.53%	0.14%	1.23%	0.50%	1.21%	2.19%	0.77%	0.65%
B. mongoliense	0.71%	0.02%	0.19%	0.07%	0.22%	0.42%	0.13%	0.08%	0.16%	0.19%	1.43%	0.08%
B. myosotis	1.40%	1.55%	0.05%	2.85%	0.00%	0.00%	1.10%	1.95%	0.08%	0.28%	0.22%	3.07%
B. pseudocatenulatum	0.38%	0.48%	0.39%	1.35%	0.10%	0.01%	0.25%	0.11%	0.31%	0.34%	0.20%	0.12%
B. pseudolongum subsp *globosum*	29.21%	25.97%	37.04%	32.91%	35.95%	18.18%	14.17%	18.39%	27.29%	17.05%	22.31%	29.17%
B. pseudolongum subsp *pseudolongum*	8.72%	7.22%	14.18%	12.81%	8.95%	5.30%	8.26%	7.87%	9.76%	7.20%	7.37%	11.80%
B. pseudolongum subspp	3.91%	1.90%	1.96%	11.96%	1.01%	4.13%	5.36%	5.46%	1.48%	2.65%	8.84%	9.57%
B. reuteri	0.79%	2.17%	1.71%	0.10%	1.59%	0.21%	0.90%	0.13%	1.68%	2.25%	0.56%	0.30%
Putative new bifidobacterial species	10.68%	12.13%	2.69%	12.09%	2.37%	4.72%	7.54%	15.54%	2.61%	8.73%	4.90%	14.72%

Taxonomy	Absolute variation (%)								
	CG			Breve2L			CG+Breve2L		
	T1-T0	T2-T0	T3-T0	T1-T0	T2-T0	T3-T0	T1-T0	T2-T0	T3-T0
B. adolescentis biotype A	0.40%	-0.18%	0.49%	0.40%	-0.16%	1.13%	0.12%	-0.38%	2.40%
B. adolescentis biotype B	0.56%	-0.25%	0.77%	0.68%	0.90%	3.94%	-0.95%	-1.79%	-1.29%
B. adolescentis biotype C	-0.78%	-0.24%	-0.95%	-1.16%	-1.03%	-0.31%	0.73%	-0.77%	-0.29%
B. animalis subsp *animalis*	-0.62%	-0.45%	0.16%	-0.07%	4.19%	7.85%	-0.14%	-0.28%	1.44%
B. animalis subsp *lactis*	1.18%	0.61%	-2.38%	-3.22%	-2.34%	-3.85%	0.99%	-3.10%	-4.05%
B. bifidum	-1.50%	-1.83%	-1.18%	-0.93%	-0.81%	-0.36%	-1.11%	-1.54%	-1.86%
B. breve	4.93%	1.71%	-9.84%	29.78%	22.93%	1.68%	6.62%	10.40%	-13.54%
B. dentium	1.38%	0.67%	0.13%	-0.29%	-0.14%	0.14%	-0.41%	-0.48%	-0.47%
B. gallinarum	-1.69%	-1.83%	-1.81%	1.78%	-0.18%	-0.15%	0.00%	0.02%	0.21%
B. kashiwanohense	0.57%	0.13%	0.01%	1.16%	0.04%	-0.02%	-0.01%	-0.08%	0.00%
B. longum subsp *longum*	-1.43%	-1.37%	-5.06%	-9.05%	-9.88%	-8.73%	-1.14%	-1.67%	-4.36%
B. longum subsp *suis*	-0.57%	-0.32%	-0.12%	-0.16%	-0.93%	-0.40%	-0.23%	-0.81%	-0.95%
B. magnum	1.16%	0.66%	2.46%	-1.38%	-0.30%	-1.02%	0.98%	-0.45%	-0.57%
B. mongoliense	-0.69%	-0.53%	-0.64%	0.20%	-0.09%	-0.14%	0.02%	1.27%	-0.08%
B. myosotis	0.15%	-1.35%	1.45%	0.00%	1.10%	1.95%	0.20%	0.14%	2.99%
B. pseudocatenulatum	0.10%	0.01%	0.97%	-0.08%	0.15%	0.01%	0.02%	-0.12%	-0.19%
B. pseudolongum subsp *globosum*	-3.24%	7.84%	3.70%	-17.77%	-21.78%	-17.56%	-10.25%	-4.98%	1.88%
B. pseudolongum subsp *pseudolongum*	-1.51%	5.46%	4.09%	-3.64%	-0.69%	-1.07%	-2.55%	-2.38%	2.04%
B. pseudolongum subspp	-2.01%	-1.95%	8.05%	3.13%	4.35%	4.46%	1.17%	7.36%	8.09%
B. reuteri	1.38%	0.91%	-0.69%	-1.38%	-0.68%	-1.46%	0.57%	-1.12%	-1.38%
Putative new bifidobacterial species	1.44%	-7.99%	1.41%	2.34%	5.17%	13.17%	6.12%	2.29%	12.11%

Taxonomy	CG T1-T0	CG T2-T0	CG T3-T0	Breve2L T1-T0	Breve2L T2-T0	Breve2L T3-T0	CG+Breve2L T1-T0	CG+Breve2L T2-T0	CG+Breve2L T3-T0
	**Trend (effect of treatment respect to T0 controls)**								
B. adolescentis biotype A	+	-	+	+	-	+	+	-	+
B. adolescentis biotype B	+	-	+	+	+	+	-	-	-
B. adolescentis biotype C	-	-	-	-	-	-	+	-	-
B. animalis subsp *animalis*	-	-	+	-	+	+	-	-	+
B. animalis subsp *lactis*	+	+	-	-	-	-	+	-	-
B. bifidum	-	-	-	-	-	-	-	-	-
B. breve	+	+	-	+	+	+	+	+	-
B. dentium	+	+	+	-	-	+	-	-	-
B. gallinarum	-	-	-	+	-	-	+	+	+
B. kashiwanohense	+	+	+	+	+	-	-	-	+
B. longum subsp *longum*	-	-	-	-	-	-	-	-	-
B. longum subsp *suis*	-	-	-	-	-	-	-	-	-
B. magnum	+	+	+	-	-	-	+	-	-
B. mongoliense	-	-	-	+	-	-	+	+	-
B. myosotis	+	-	+		+	+	+	+	+
B. pseudocatenulatum	+	+	+	-	+	+	+	-	-
B. pseudolongum subsp *globosum*	-	+	+	-	-	-	-	-	+
B. pseudolongum subsp *pseudolongum*	-	+	+	-	-	-	-	-	+
B. pseudolongum subspp	-	-	+	+	+	+	+	+	+
B. reuteri	+	+	-	-	-	-	+	-	-
Putative new bifidobacterial species	+	-	+	+	+	+	+	+	+

	t-test p-value < 0.05								
	CG			Breve2L			CG+Breve2L		
**Taxonomy**	**T1-T0**	**T2-T0**	**T3-T0**	**T1-T0**	**T2-T0**	**T3-T0**	**T1-T0**	**T2-T0**	**T3-T0**
B. adolescentis biotype A		0.022							
B. adolescentis biotype B									
B. adolescentis biotype C									
B. animalis subsp *animalis*				0.021	0.014			0.004	0.020
B. animalis subsp *lactis*			0.029			0.015			0.044
B. bifidum		0.016					0.030	0.009	0.008
B. breve									
B. dentium			0.001						
B. gallinarum	0.017	0.006	0.034		0.000	0.044			
B. kashiwanohense		0.036							
B. longum subsp *longum*	0.015	0.039	0.008		0.004	0.007	0.034		0.010
B. longum subsp *suis*								0.001	
B. magnum			0.001	0.021					
B. mongoliense	0.011	0.030	0.042						
B. myosotis		0.006			0.001	0.040			0.011
B. pseudocatenulatum				0.034					
B. pseudolongum subsp *globosum*				0.041	0.005	0.020			
B. pseudolongum subsp *pseudolongum*				0.004	0.014				
B. pseudolongum subspp		0.027		0.001					0.043
B. reuteri				0.000	0.015	0.000		0.039	
Putative new bifidobacterial species		0.019			0.002	0.002	0.001	0.033	0.010
