# Supplementary material for: Ability of bifidobacteria to metabolize chitin-glucan and its impact on the gut microbiota
Source: Sci Rep. 2019 Apr 8;9:5755. doi: 10.1038/s41598-019-42257-z (PMC6453949; doi:10.1038/s41598-019-42257-z)
Supplement: Supplementary file 2 — Supplementary Tables [file 41598_2019_42257_MOESM2_ESM.pdf]

Ability of bifidobacteria to metabolize chitin-glucan and its impact on the gut microbiota

Key words: Bifidobacterium, prebiotics, gut microbiota, microbe-microbe interactions

Running title: Bifidobacteria and chitin glucan

Giulia Alessandri[1#], Christian Milani[2#], Sabrina Duranti[2#], Leonardo Mancabelli[2], Thibaut Ranjanoro[3], Salvatore Modica[3], Luca Carnevali[4], Rosario Statello[4], Francesca Bottacini[5], Francesca Turroni[2], Maria Cristina Ossiprandi[1], Andrea Sgoifo[4], Dowue van Sinderen[5] and Marco Ventura[1]

[#] These authors contributed equally

Department of Veterinary Medical Science, University of Parma, Parma, Italy[1]; Laboratory of Probiogenomics, Department of Chemistry, Life Sciences, and Environmental Sustainability, University of Parma, Parma, Italy[2]; KitoZyme, Belgium[3]; Stress Physiology Laboratory, Department of Chemistry, Life Sciences and Environmental Sustainability, University of Parma, Parma, Italy[4]; APC Microbiome Institute and School of Microbiology, Bioscience Institute, National University of Ireland, Cork, Ireland[5]

Correspondence. Mailing address for Marco Ventura; Laboratory of Probiogenomics, Department of Chemistry, Life Sciences, and Environmental Sustainability, University of Parma, Parco Area delle Scienze 11a, 43124 Parma, Italy. Phone: ++39-521-905666. Fax: ++39-521-905604. E-mail: marco.ventura@unipr.it

Table S1: The 100 most over-expressed genes in *B. breve* 2L cultivated in MRS +CG, sorted by CG average RPKM.

ORFs	CG average RPKM	MRS average RPKM	CG expression induction CG/MRS	Length (aa)	Annotation
B2L_0179	34762	16321	2.1	38	LSU ribosomal protein L36P
B2L_0967	24685	4987	4.9	308	**Solute binding protein of ABC transporter system**
B2L_1102	21932	15616	1.4	90	SSU ribosomal protein S15P
B2L_1128	16109	7300	2.2	65	LSU ribosomal protein L32P
B2L_0970	15117	922	16.4	114	Ferric uptake regulation protein
B2L_0141	14921	13773	1.1	150	50S ribosomal protein L13
B2L_0944	12433	12016	1	400	Protein Translation Elongation Factor Tu (EF-TU)
B2L_0407	10895	7863	1.4	94	DNA-binding protein HU
B2L_0166	9512	6684	1.4	123	50S ribosomal protein L14
B2L_0774	9174	36111	0.3	168	Hsp20-family heat shock chaperone
B2L_0155	8357	9709	0.9	103	30S ribosomal protein S10
B2L_0180	7719	7628	1	126	SSU ribosomal protein S13P
B2L_0320	7560	6456	1.2	71	LSU ribosomal protein L31P
B2L_0877	7124	8334	0.9	376	**ATP-binding protein of ABC transporter system for sugars**
B2L_0067	6889	9554	0.7	144	LSU ribosomal protein L11P
B2L_0677	6150	4528	1.4	87	Phosphocarrier protein HPr
B2L_0818	5986	4590	1.3	188	Alkyl hydroperoxide reductase C22 protein
B2L_1771	5889	2509	2.3	173	Transcriptional regulator
B2L_0119	5571	5700	1	98	10 kDa chaperonin GROES
B2L_0167	5546	9016	0.6	112	50S ribosomal protein L24
B2L_0142	5399	9388	0.6	164	30S ribosomal protein S9
B2L_0822	4965	3577	1.4	160	Hypothetical protein in DPS family
B2L_0947	4944	3047	1.6	124	SSU ribosomal protein S12P
B2L_1638	4827	6200	0.8	280	SSU ribosomal protein S2P
B2L_0200	4698	2193	2.1	144	Rrf2 family protein
B2L_1406	4690	2849	1.6	792	Formate acetyltransferase
B2L_0120	4588	6623	0.7	56	LSU ribosomal protein L33P
B2L_0224	4379	4092	1.1	257	Phosphoglycerate mutase
B2L_0168	4250	10550	0.4	191	50S ribosomal protein L5
B2L_1823	4169	2787	1.5	353	Glyceraldehyde 3-phosphate dehydrogenase
B2L_1272	4012	3183	1.3	210	LSU ribosomal protein L25P
B2L_0174	3694	11175	0.3	62	50S ribosomal protein L30
B2L_0109	3679	9569	0.4	174	LSU ribosomal protein L10P
B2L_1310	3647	5489	0.7	209	SSU ribosomal protein S4P
B2L_1417	3622	6116	0.6	826	Xylulose-5-phosphate/Fructose-6-phosphate phosphoketolase
B2L_1767	3561	3798	0.9	221	Ribosome-associated factor Y
B2L_0160	3248	7761	0.4	93	30S ribosomal protein S19
B2L_0727	3215	2837	1.1	627	Chaperone protein dnaK

B2L_0420	3185	1618	2	80	Cold shock protein
B2L_0158	3169	9259	0.3	99	50S ribosomal protein L23
B2L_1238	3122	1867	1.7	318	Choloylglycine hydrolase
B2L_0626	3034	6646	0.5	97	SSU ribosomal protein S6P
B2L_0726	2915	2552	1.1	228	GrpE protein
B2L_1455	2905	5476	0.5	492	SSU ribosomal protein S1P
B2L_0170	2900	7830	0.4	133	30S ribosomal protein S8
B2L_0156	2886	6171	0.5	214	50S ribosomal protein L3
B2L_0058	2871	1937	1.5	103	LSU ribosomal protein L21P
B2L_1143	2818	3460	0.8	122	LSU ribosomal protein L19P
B2L_0305	2773	1814	1.5	188	Hypoxanthine-guanine phosphoribosyltransferase
B2L_0795	2669	1988	1.3	128	hypothetical protein
B2L_1206	2656	2203	1.2	368	Transaldolase
B2L_1268	2588	3429	0.8	87	SSU ribosomal protein S20P
B2L_1864	2566	2767	0.9	321	L-lactate dehydrogenase
B2L_0495	2544	2965	0.9	449	**ABC transporter solute-binding protein, family 1**
B2L_0581	2481	2065	1.2	544	ATP synthase alpha chain
B2L_1819	2425	3615	0.7	267	Bacterial Protein Translation Initiation Factor 3 (IF-3)
B2L_0110	2409	15731	0.2	127	LSU ribosomal protein L12P (L7/L12)
B2L_0059	2357	1805	1.3	84	LSU ribosomal protein L27P
B2L_0157	2330	6772	0.3	219	50S ribosomal protein L4
B2L_0181	2314	3911	0.6	133	SSU ribosomal protein S11P
B2L_0182	2256	3186	0.7	332	DNA-directed RNA polymerase alpha chain
B2L_0578	2233	2004	1.1	98	ATP synthase epsilon chain
B2L_0169	2180	8145	0.3	62	30S ribosomal protein S14-1
B2L_0787	2161	762	2.8	154	Hypothetical membrane spanning protein in uncharacterized protein family (UPF0233)
B2L_0868	2145	1735	1.2	167	NrdI protein
B2L_0087	2139	3309	0.6	65	LSU ribosomal protein L28P
B2L_0165	2109	4637	0.5	87	30S ribosomal protein S17
B2L_0585	2103	1110	1.9	270	ATP synthase A chain
B2L_0068	2099	5979	0.4	231	LSU ribosomal protein L1P
B2L_0171	2098	3320	0.6	180	50S ribosomal protein L6
B2L_0589	2062	1960	1.1	165	Inorganic pyrophosphatase
B2L_1588	2057	1878	1.1	188	Protein Translation Elongation Factor P (EF-P)
B2L_1744	2056	686	3	98	hypothetical protein
B2L_0867	2014	1130	1.8	89	Glutaredoxin nrdH
B2L_0580	2002	2566	0.8	308	ATP synthase gamma chain
B2L_0584	1953	1308	1.5	77	ATP synthase C chain
B2L_0421	1895	4241	0.4	542	60 kDa chaperonin GroEL
B2L_0161	1823	3688	0.5	120	50S ribosomal protein L22
B2L_0996	1818	1066	1.7	393	putative membrane spanning protein
B2L_0103	1810	140	13	377	Phosphate-binding protein

B2L_0173	1782	5124	0.3	243	30S ribosomal protein S5
B2L_0207	1777	7152	0.2	439	**extracellular solute-binding protein, family 1**
B2L_0624	1777	6645	0.3	83	SSU ribosomal protein S18P
B2L_1005	1741	973	1.8	165	Autoinducer-2 production protein luxS
B2L_0159	1707	3156	0.5	277	50S ribosomal protein L2
B2L_1818	1699	1374	1.2	65	LSU ribosomal protein L35P
B2L_0175	1683	5447	0.3	151	50S ribosomal protein L15
B2L_1861	1674	146	11.5	128	LysM domain protein
B2L_0583	1573	456	3.4	173	ATP synthase B chain
B2L_0579	1559	2028	0.8	491	ATP synthase beta chain
B2L_1828	1556	1578	1	103	hypothetical protein
B2L_0354	1555	672	2.3	803	Anaerobic ribonucleoside-triphosphate reductase
B2L_0625	1541	5183	0.3	215	Single-strand DNA binding protein
B2L_1451	1541	1406	1.1	481	Pyruvate kinase
B2L_1027	1538	520	3	159	SsrA-binding protein
B2L_0203	1526	2234	0.7	697	**PTS system, fructose/glucose-specific IIABC component**
B2L_1116	1497	2550	0.6	166	SSU ribosomal protein S16P
B2L_0172	1489	4909	0.3	124	50S ribosomal protein L18
B2L_0243	1464	829	1.8	442	**family 1 extracellular solute-binding protein**

Table S2: The 100 most over-expressed genes in *B. breve* 2L cultivated in MRS +CG respect to *B. breve* 2L grown in MRS, sorted by CG expression induction (CG/MRS).

ORFs	CG average RPKM	MRS average RPKM	CG expression induction CG/MRS	Length (aa)	Annotation
B2L_1430	346	1	571.7	109	hypothetical protein
B2L_1711	126	1	148.6	542	mobilization protein
B2L_0714	360	3	111	96	Hypothetical protein, TadE-like
B2L_0148	201	2	106	446	**D-arabitol membrane transporter**
B2L_1705	252	3	100.4	144	hypothetical protein
B2L_1706	426	5	94.7	101	hypothetical protein
B2L_0712	287	4	79.4	113	putative secreted protein
B2L_0718	327	5	72.6	269	Septum site-determining protein minD, TadZ-like
B2L_0256	259	4	72.6	135	mobilization protein
B2L_1759	227	3	72.2	213	hypothetical protein
B2L_0252	116	2	71.6	203	hypothetical protein
B2L_1701	661	12	57.3	211	hypothetical protein
B2L_0257	61	1	52	477	Relaxase
B2L_0565	229	5	47.8	313	**Permease protein of ABC transporter system**
B2L_0713	155	3	47.8	125	TadF-like protein
B2L_1722	100	2	45.9	568	hypothetical protein
B2L_1723	99	3	38.8	798	transfer complex protein
B2L_0255	103	3	35.1	123	hypothetical protein
B2L_0564	109	3	34.5	304	**Permease protein of ABC transporter system**
B2L_0270	136	4	32.9	543	Hypothetical protein with CHAP and transglycosylase SLT domains
B2L_0258	17	1	32.4	290	hypothetical protein
B2L_0886	160	6	27.8	287	putative secreted protein
B2L_1707	207	8	27.2	114	hypothetical protein
B2L_0269	137	5	26.8	432	hypothetical protein
B2L_1574	655	28	23.3	56	Cystathionine beta-lyase
B2L_1389	126	6	22.9	512	ATPase
B2L_1328	92	4	22.8	296	cation-transporting P-type ATPase
B2L_1424	122	5	22.4	487	Transposase
B2L_0948	162	7	21.8	218	Transporter, Sodium/bile acid symporter family, macrolide-resistance protein
B2L_0271	188	9	20.6	792	transfer complex protein
B2L_0398	79	4	20.4	143	Nitrogen regulatory IIA (Enzyme IIA-NTR)
B2L_1421	181	9	19.8	185	hypothetical protein
B2L_1429	117	6	19.3	911	putative type III restriction system endonuclease
B2L_0259	52	3	18.7	335	hypothetical protein
B2L_1751	369	20	18.7	87	hypothetical protein
B2L_1781	160	9	18.5	220	Transcriptional regulatory protein
B2L_1667	181	10	18.2	569	ComE operon protein 3

B2L_1581	745	41	18	154	Hypothetical protein, very proline rich
B2L_1431	97	5	17.8	73	hypothetical protein
B2L_1313	626	36	17.4	132	putative membrane spanning protein
B2L_0399	153	9	16.8	157	**PTS system, fructose-like IIA component**
B2L_0517	359	22	16.6	578	**extracellular solute-binding protein**
B2L_0970	15117	922	16.4	114	Ferric uptake regulation protein
B2L_0273	68	4	16	282	putative membrane spanning protein
B2L_0402	229	14	15.9	239	Ribulose-phosphate 3-epimerase
B2L_0463	432	28	15.6	442	**Sucrose permease**
B2L_0563	125	8	15.6	438	**ATP-binding protein of ABC transporter system**
B2L_0731	317	21	15.4	307	**Maltose transport system permease protein malG**
B2L_0717	32	2	15.2	340	hypothetical protein
B2L_0716	40	3	14.9	149	putative membrane spanning protein, TadB-like protein
B2L_0462	282	19	14.6	519	**Beta-fructofuranosidase**
B2L_1731	76	5	14.5	222	plasmid partition protein ParA
B2L_1388	246	17	14.4	125	Endonuclease
B2L_0603	141	10	14	80	transposase
B2L_0732	144	10	14	467	**Maltodextrin transport system permease protein malC**
B2L_1086	179	13	13.8	251	Sulfatase family protein
B2L_0253	97	7	13	526	Chromosome partitioning protein parB
B2L_0103	1810	140	13	377	Phosphate-binding protein
B2L_1716	78	6	12.6	611	TraG family protein
B2L_0397	47	4	12.2	498	Transcription antiterminator, BglG family
B2L_0734	126	10	12.2	629	**Alpha-glucosidase**
B2L_0555	238	20	12.1	367	**putative ABC transporter, permease protein**
B2L_1704	49	4	12	741	DNA topoisomerase III
B2L_1712	63	5	11.8	358	hypothetical protein
B2L_1861	1674	146	11.5	128	LysM domain protein
B2L_1721	39	3	11.4	180	hypothetical protein
B2L_0016	110	10	11.2	675	hypothetical protein
B2L_1419	207	19	11.1	138	putative transcriptional regulator
B2L_1713	65	6	10.8	673	hypothetical protein
B2L_0260	73	7	10.8	383	hypothetical protein
B2L_1390	94	9	10.8	540	Smf protein
B2L_1653	130	12	10.5	365	**ABC transporter ATP-binding protein**
B2L_0650	465	46	10.2	875	putative membrane spanning protein
B2L_1738	322	32	10	238	hypothetical protein
B2L_0247	104	10	9.9	494	**Galactoside symporter**
B2L_1154	124	13	9.8	224	**family 1 extracellular solute-binding protein of ABC transporter system**
B2L_0551	498	51	9.7	495	xylulokinase
B2L_1439	232	24	9.7	225	hypothetical protein

B2L_1088	214	22	9.7	304	**Permease of ABC transporter for sugars**
B2L_1724	78	8	9.5	117	hypothetical protein
B2L_1247	177	19	9.4	245	Transcriptional activator tenA
B2L_0552	615	67	9.2	345	**ABC transporter, permease protein**
B2L_1740	682	74	9.2	74	hypothetical protein
B2L_0263	65	7	9.1	583	TraG/TraD family
B2L_1699	187	21	9	145	hypothetical protein
B2L_0401	63	7	9	373	**PTS system, fructose-like enzyme IIC component**
B2L_0250	178	20	8.9	322	Integrase protein
B2L_0515	138	16	8.8	333	Oligopeptide transport system permease protein oppC
B2L_1044	89	10	8.8	430	Hypothetical protein with DUF58 domain
B2L_0549	271	31	8.8	104	putative secreted protein
B2L_1736	353	40	8.7	52	hypothetical protein
B2L_0318	182	21	8.6	264	hypothetical protein
B2L_0932	207	25	8.3	835	Excinuclease ABC subunit A
B2L_1352	122	15	8.2	535	**Glycogen or amylose phosphorylase**
B2L_0743	318	39	8.2	456	**Transporter, MFS superfamily**
B2L_1087	85	10	8.1	459	**extracellular solute-binding protein, family 1**
B2L_0689	134	17	8.1	50	N-acetylglucosamine-6-phosphate deacetylase
B2L_0053	193	24	8.1	256	hypothetical protein
B2L_0587	208	27	7.6	645	putative secreted protein
B2L_0284	77	11	7.1	1461	Cell surface protein

Table S3: The most over-expressed genes loci in *B. breve* 2L supplemented with CG respect to *B. breve* 2L in MRS.

ORFs	CG average RPKM	MRS average RPKM	CG expression induction CG/MRS	Length (aa)	Annotation
B2L_0246	99	19	5.3	1164	**Beta-galactosidase**
B2L_0247	104	10	9.9	494	**Galactoside symporter**
B2L_0248	188	41	4.6	88	Site-specific recombinase
B2L_0250	178	20	8.9	322	Integrase protein
B2L_0251	175	37	4.7	222	hypothetical protein
B2L_0252	116	2	71.6	203	hypothetical protein
B2L_0253	97	7	13	526	Chromosome partitioning protein parB
B2L_0254	13	38	0.3	109	hypothetical protein
B2L_0255	103	3	35.1	123	hypothetical protein
B2L_0256	259	4	72.6	135	mobilization protein
B2L_0257	61	1	52	477	Relaxase
B2L_0258	17	1	32.4	290	hypothetical protein
B2L_0259	52	3	18.7	335	hypothetical protein
B2L_0260	73	7	10.8	383	hypothetical protein
B2L_0262	31	11	2.7	127	hypothetical protein
B2L_0263	65	7	9.1	583	TraG/TraD family
B2L_0269	137	5	26.8	432	hypothetical protein
B2L_0270	136	4	32.9	543	Hypothetical protein with CHAP and transglycosylase SLT domains
B2L_0271	188	9	20.6	792	transfer complex protein
B2L_0273	68	4	16	282	putative membrane spanning protein
B2L_0275	102	18	5.8	371	Transposase
B2L_0281	269	44	6.1	180	putative secreted protein
B2L_0282	202	61	3.3	334	Penicillin acylase, bile salt hydrolase
B2L_0283	426	98	4.3	262	Multidrug-efflux transporter 2 regulator
B2L_0284	77	11	7.1	1461	Cell surface protein
B2L_0397	47	4	12.2	498	Transcription antiterminator, BglG family
B2L_0398	79	4	20.4	143	Nitrogen regulatory IIA (Enzyme IIA-NTR)
B2L_0399	153	9	16.8	157	**PTS system, fructose-like IIA component**
B2L_0400	0	6	0	104	**PTS system, fructose-like IIB component**
B2L_0401	63	7	9	373	**PTS system, fructose-like enzyme IIC component**
B2L_0402	229	14	15.9	239	Ribulose-phosphate 3-epimerase
B2L_0514	71	14	5.2	328	Oligopeptide transport system permease protein oppB
B2L_0515	138	16	8.8	333	Oligopeptide transport system permease protein oppC
B2L_0516	171	25	6.9	713	Oligopeptide transport ATP-binding protein oppD

B2L_0517	359	22	16.6	578	extracellular solute-binding protein
B2L_0549	271	31	8.8	104	putative secreted protein
B2L_0550	131	118	1.1	433	putative ROK family protein
B2L_0551	498	51	9.7	495	xylulokinase
B2L_0552	615	67	9.2	345	**ABC transporter, permease protein**
B2L_0553	336	50	6.7	513	**ABC transporter, permease protein**
B2L_0554	274	39	6.9	344	**ABC transporter, permease protein**
B2L_0555	238	20	12.1	367	**putative ABC transporter, permease protein**
B2L_0556	512	72	7.1	193	**sugar isomerase (SIS)**
B2L_0563	125	8	15.6	438	**ATP-binding protein of ABC transporter system**
B2L_0564	109	3	34.5	304	**Permease protein of ABC transporter system**
B2L_0565	229	5	47.8	313	**Permease protein of ABC transporter system**
B2L_0566	90	14	6.6	389	**Solute binding protein of ABC transporter system**
B2L_0598	154	48	3.2	601	**putative alpha-galactosidase**
B2L_0599	169	53	3.2	290	**sugar ABC transporter permease**
B2L_0600	209	66	3.2	297	**sugar ABC transporter permease**
B2L_0601	202	78	2.6	441	**extracellular solute-binding protein**
B2L_0602	330	357	0.9	292	putative transcriptional regulator, AraC family
B2L_0603	141	10	14	80	transposase
B2L_0604	194	34	5.6	124	transposase
B2L_0712	287	4	79.4	113	**putative secreted protein**
B2L_0713	155	3	47.8	125	**TadF-like protein**
B2L_0714	360	3	111	96	**Hypothetical protein, TadE-like**
B2L_0715	3	11	0.3	219	**putative secreted protein, TadC-like protein**
B2L_0716	40	3	14.9	149	**putative membrane spanning protein, TadB-like protein**
B2L_0717	32	2	15.2	340	**hypothetical protein**
B2L_0718	327	5	72.6	269	**Septum site-determining protein minD, TadZ-like**
B2L_0730	370	64	5.8	268	**putative membrane spanning protein**
B2L_0731	317	21	15.4	307	**Maltose transport system permease protein malG**
B2L_0732	144	10	14	467	**Maltodextrin transport system permease protein malC**
B2L_0733	281	64	4.4	414	**Maltose/maltodextrin-binding protein**
B2L_0734	126	10	12.2	629	**Alpha-glucosidase**
B2L_1085	146	39	3.8	200	Sulfatase family protein
B2L_1086	179	13	13.8	251	Sulfatase family protein
B2L_1087	85	10	8.1	459	**extracellular solute-binding protein, family 1**
B2L_1088	214	22	9.7	304	**Permease of ABC transporter for sugars**

B2L_1089	174	47	3.7	325	**Permease of ABC transporter for sugars**
B2L_1419	207	19	11.1	138	putative transcriptional regulator
B2L_1420	62	9	7.1	183	Relaxase/mobilization nuclease domain
B2L_1421	181	9	19.8	185	hypothetical protein
B2L_1422	238	165	1.4	387	hypothetical protein
B2L_1423	690	343	2	220	hypothetical protein
B2L_1424	122	5	22.4	487	Transposase
B2L_1425	65	13	5	169	IstB domain protein ATP-binding protein
B2L_1427	397	135	3	96	transposase
B2L_1429	117	6	19.3	911	putative type III restriction system endonuclease
B2L_1430	346	1	571.7	109	hypothetical protein
B2L_1431	97	5	17.8	73	hypothetical protein
B2L_1432	397	57	6.9	230	hypothetical protein
B2L_1688	149	22	6.8	1051	**Alpha-mannosidase**
B2L_1689	68	10	7	1040	**Alpha-mannosidase**
B2L_1690	846	161	5.3	128	phage integrase family protein
B2L_1692	199	83	2.4	326	Fic family protein
B2L_1693	149	151	1	438	hypothetical protein
B2L_1695	152	247	0.6	135	putative secreted protein
B2L_1696	169	65	2.6	448	phage integrase family protein
B2L_1697	835	395	2.1	256	filamentation induced by cAMP protein Fic
B2L_1699	187	21	9	145	hypothetical protein
B2L_1700	102	46	2.2	138	hypothetical protein
B2L_1701	661	12	57.3	211	hypothetical protein
B2L_1704	49	4	12	741	DNA topoisomerase III
B2L_1705	252	3	100.4	144	hypothetical protein
B2L_1706	426	5	94.7	101	hypothetical protein
B2L_1707	207	8	27.2	114	hypothetical protein
B2L_1708	702	479	1.5	86	hypothetical protein
B2L_1709	982	838	1.2	83	hypothetical protein
B2L_1710	0	3	0	127	hypothetical protein
B2L_1711	126	1	148.6	542	mobilization protein
B2L_1712	63	5	11.8	358	hypothetical protein
B2L_1713	65	6	10.8	673	hypothetical protein
B2L_1716	78	6	12.6	611	TraG family protein
B2L_1717	214	117	1.8	168	hypothetical protein
B2L_1718	457	784	0.6	320	adenine-specific DNA methyltransferase
B2L_1719	692	900	0.8	488	hypothetical protein
B2L_1720	192	52	3.7	452	hypothetical protein
B2L_1721	39	3	11.4	180	hypothetical protein
B2L_1722	100	2	45.9	568	hypothetical protein

B2L_1723	99	3	38.8	798	transfer complex protein
B2L_1724	78	8	9.5	117	hypothetical protein
B2L_1725	34	16	2.1	282	hypothetical protein
B2L_1726	14	29	0.5	78	putative membrane spanning protein
B2L_1727	81	25	3.3	1789	Cell surface protein
B2L_1728	81	42	1.9	353	hypothetical protein
B2L_1729	147	26	5.7	163	hypothetical protein
B2L_1730	11	8	1.4	94	hypothetical protein
B2L_1731	76	5	14.5	222	plasmid partition protein ParA
B2L_1732	11	5	2.2	166	hypothetical protein
B2L_1733	46	7	6.8	85	hypothetical protein
B2L_1734	159	22	7	89	hypothetical protein
B2L_1735	170	43	3.9	288	hypothetical protein
B2L_1736	353	40	8.7	52	hypothetical protein
B2L_1737	55	18	3.1	64	hypothetical protein
B2L_1738	322	32	10	238	hypothetical protein
B2L_1739	103	27	3.8	123	hypothetical protein
B2L_1740	682	74	9.2	74	hypothetical protein

Table S4: 16S rRNA gene microbial profiling quality filtering table.

Sample Group	Time Point	Mouse ID	Sample	Number of sequenced pe reads	Number of pe reads with mean quality > 20	Number of merged pe reads	Non-bacterial sequences	Length outside bounds of 100 and 400	Ambiguous bases	Homopolymers > 7	Mismatch in primers >1	Reverse primer not found	Final Read Number
CG	T_0	1	**WT-1A**	66035	18816	18342	3	0	0	3	383	3	17950
CG	T_1	1	**WT-1B**	98615	33864	32053	8	0	0	19	12149	7	19870
CG	T_2	1	**WT-1C**	73988	51454	49516	2	0	0	23	2606	24	46861
CG	T_3	1	**WT-1D**	45607	44227	43582	16	0	0	4	943	17	42602
CG	T_0	2	**WT-2A**	54386	45743	44896	0	0	0	3	917	12	43964
CG	T_1	2	**WT-2B**	98616	89695	86081	4	0	1	20	1711	40	84305
CG	T_2	2	**WT-2C**	82401	44669	42675	0	0	1	16	8428	12	34218
CG	T_3	2	**WT-2D**	73244	65784	63080	2	0	2	21	3926	38	59091
CG	T_0	3	**WT-3A**	86982	37229	35981	0	0	0	13	5065	19	30884
CG	T_1	3	**WT-3B**	92483	71531	69418	0	0	0	17	3495	33	65873
CG	T_2	3	**WT-3C**	14401	688	640	0	0	0	0	384	0	256
CG	T_3	3	**WT-3D**	388903	378255	370448	19	0	0	53	11198	255	358923
CG	T_0	4	**WT-4A**	56149	54106	52833	0	0	0	6	974	19	51834
CG	T_1	4	**WT-4B**	6760	4206	4112	1	0	0	1	146	2	3962
CG	T_2	4	**WT-4C**	75216	37913	36128	3	0	0	15	7349	10	28751
CG	T_3	4	**WT-4D**	36569	34984	34371	3	0	0	1	974	6	33387
CG	T_0	5	**WT-5A**	36637	28152	27597	3	0	0	7	830	9	26748
CG	T_1	5	**WT-5B**	48916	46908	46165	0	0	0	7	875	15	45268
CG	T_2	5	**WT-5C**	78437	56811	54411	4	0	0	24	3239	15	51129
CG	T_3	5	**WT-5D**	91571	86894	83631	2	0	2	37	1326	50	82214
CG	T_0	6	**WT-6A**	50907	49512	48787	0	0	0	9	835	19	47924
CG	T_1	6	**WT-6B**	69118	62624	60887	5	0	1	11	2743	11	58116
CG	T_2	6	**WT-6C**	37208	34806	33361	2	0	0	12	1273	18	32056
CG	T_3	6	**WT-6D**	56051	40839	39842	4	0	0	12	6390	7	33429
CG	T_0	7	**WT-7A**	68553	66253	63674	7	0	0	28	1134	28	62477
CG	T_1	7	**WT-7B**	84075	66572	63924	6	0	2	24	17906	21	45965
CG	T_2	7	**WT-7C**	69716	65421	62660	6	0	1	16	10525	30	52082
CG	T_3	7	**WT-7D**	80578	36532	35037	4	0	1	7	1178	22	33825
CG	T_0	8	**WT-8A**	96574	84202	80624	4	0	0	26	28385	18	52191
CG	T_1	8	**WT-8B**	83792	77204	73054	7	0	1	25	6746	44	66231
CG	T_2	8	**WT-8C**	69987	66827	64402	2	0	0	13	4404	41	59942
CG	T_3	8	**WT-8D**	54056	48084	46894	1	0	0	13	6577	8	40295
Breve2L	T_0	9	**WT-9A**	58274	56973	56011	1	0	0	9	1385	24	54592
Breve2L	T_1	9	**WT-9B**	75468	73286	70395	2	0	1	21	3566	38	66767
Breve2L	T_2	9	**WT-9C**	77640	71922	68908	5	0	0	21	1715	23	67144
Breve2L	T_3	9	**WT-9D**	71572	69344	66897	1	0	0	19	2545	38	64294

Breve2L	T_0	10	**WT-10A**	40653	25414	24970	0	0	0	5	907	8	24050
Breve2L	T_1	10	**WT-10B**	40673	37181	36633	0	0	0	2	669	12	35950
Breve2L	T_2	10	**WT-10C**	76059	70649	68022	6	0	1	17	4706	24	63268
Breve2L	T_3	10	**WT-10D**	43732	41013	40444	1	0	0	4	959	10	39470
Breve2L	T_0	11	**WT-11A**	47837	46397	45640	1	0	0	5	1104	12	44518
Breve2L	T_1	11	**WT-11B**	45754	42486	41801	1	0	0	6	1005	8	40781
Breve2L	T_2	11	**WT-11C**	59288	49456	47199	3	0	1	14	17190	8	29982
Breve2L	T_3	11	**WT-11D**	85559	82354	78987	5	0	1	24	1750	41	77166
Breve2L	T_0	12	**WT-12A**	67438	60819	58543	3	0	1	21	1702	23	56793
Breve2L	T_1	12	**WT-12B**	82596	79980	76668	3	0	0	19	9945	36	66665
Breve2L	T_2	12	**WT-12C**	80399	59881	57674	3	0	0	35	2233	20	55383
Breve2L	T_3	12	**WT-12D**	82495	44645	43157	3	0	0	44	16292	2	26816
Breve2L	T_0	13	**WT-13A**	31999	30773	30298	0	0	0	3	668	6	29621
Breve2L	T_1	13	**WT-13B**	47635	45345	44601	0	0	0	5	845	11	43740
Breve2L	T_2	13	**WT-13C**	67693	41807	40994	0	0	0	7	3286	11	37690
Breve2L	T_3	13	**WT-13D**	67327	60704	59094	1	0	0	6	8652	21	50414
Breve2L	T_0	14	**WT-14A**	79958	70556	67890	2	0	2	15	4750	37	63084
Breve2L	T_1	14	**WT-14B**	44561	43290	42629	0	0	0	7	784	10	41828
Breve2L	T_2	14	**WT-14C**	68888	63064	60639	0	0	2	15	2608	25	57989
Breve2L	T_3	14	**WT-14D**	60063	57380	56534	1	0	0	8	1403	13	55109
Breve2L	T_0	15	**WT-15A**	73127	65361	63275	5	0	0	20	7914	31	55305
Breve2L	T_1	15	**WT-15B**	38841	37855	37258	0	0	0	3	831	16	36408
Breve2L	T_2	15	**WT-15C**	72741	63848	61722	7	0	1	15	3685	26	57988
Breve2L	T_3	15	**WT-15D**	45916	42421	41833	5	0	0	7	971	17	40833
Breve2L	T_0	16	**WT-16A**	42058	41437	40777	2	0	0	15	964	26	39770
Breve2L	T_1	16	**WT-16B**	50832	50293	49521	2	0	0	9	1037	20	48453
Breve2L	T_2	16	**WT-16C**	72101	64092	61830	3	0	1	16	2356	25	59429
Breve2L	T_3	16	**WT-16D**	58222	15314	14818	0	0	0	3	3822	6	10987
CG+Breve2L	T_0	17	**WT-17A**	63236	57536	55977	1	0	0	19	3423	27	52507
CG+Breve2L	T_1	17	**WT-17B**	88868	22177	20640	7	0	0	4	9353	4	11272
CG+Breve2L	T_2	17	**WT-17C**	63726	57768	55622	2	0	1	25	5419	33	50142
CG+Breve2L	T_3	17	**WT-17D**	60263	58194	57418	1	0	0	6	1407	19	55985
CG+Breve2L	T_0	18	**WT-18A**	48706	47362	46621	0	0	0	8	1004	15	45594
CG+Breve2L	T_1	18	**WT-18B**	64647	55263	52913	6	0	1	35	5014	17	47840
CG+Breve2L	T_2	18	**WT-18C**	100580	68180	65442	2	0	1	30	6154	30	59225
CG+Breve2L	T_3	18	**WT-18D**	68705	48962	48133	3	0	0	5	2294	11	45820
CG+Breve2L	T_0	19	**WT-19A**	97380	56292	53992	3	0	1	31	5018	23	48916
CG+Breve2L	T_1	19	**WT-19B**	79803	31190	29799	3	0	0	9	5767	11	24009
CG+Breve2L	T_2	19	**WT-19C**	100801	34012	32276	9	0	0	10	3001	14	29242
CG+Breve2L	T_3	19	**WT-19D**	53220	49302	48580	3	0	0	11	1228	17	47321
CG+Breve2L	T_0	20	**WT-20A**	49572	48882	48015	3	0	0	7	959	6	47040
CG+Breve2L	T_1	20	**WT-20B**	68842	67123	64658	2	0	1	15	3316	26	61298
CG+Breve2L	T_2	20	**WT-20C**	98311	27120	25671	2	0	0	15	8596	12	17046
CG+Breve2L	T_3	20	**WT-20D**	68057	60312	58165	0	0	1	12	1778	25	56349
CG+Breve2L	T_0	21	**WT-21A**	65884	51767	49755	4	0	0	17	1375	23	48336

CG+Breve2L	T_1	21	**WT-21B**	80559	72085	69126	3	0	4	22	6779	33	62285
CG+Breve2L	T_2	21	**WT-21C**	122242	37388	36389	0	0	0	9	6299	6	30075
CG+Breve2L	T_3	21	**WT-21D**	59304	56314	55236	1	0	0	7	2574	18	52636
CG+Breve2L	T_0	22	**WT-22A**	34663	33456	32966	3	0	0	4	646	6	32307
CG+Breve2L	T_1	22	**WT-22B**	73540	31130	29934	4	0	1	11	2181	5	27732
CG+Breve2L	T_2	22	**WT-22C**	82779	43818	41480	7	0	1	21	9654	21	31776
CG+Breve2L	T_3	22	**WT-22D**	69278	67414	64789	2	0	1	21	2362	42	62361
CG+Breve2L	T_0	23	**WT-23A**	71319	37955	36325	3	0	3	20	6721	6	29572
CG+Breve2L	T_1	23	**WT-23B**	74124	51526	49143	10	0	1	19	17554	13	31546
CG+Breve2L	T_2	23	**WT-23C**	55717	19363	18944	0	0	0	20	1342	2	17580
CG+Breve2L	T_3	23	**WT-23D**	63689	57747	56798	5	0	0	3	2967	19	53804
CG+Breve2L	T_0	24	**WT-24A**	39593	38884	38260	1	0	0	4	977	18	37260
CG+Breve2L	T_1	24	**WT-24B**	72335	60879	58485	5	0	0	10	4836	18	53616
CG+Breve2L	T_2	24	**WT-24C**	46807	19559	19112	1	0	0	0	1300	7	17804
CG+Breve2L	T_3	24	**WT-24D**	74482	45140	44184	0	0	0	6	5871	10	38297

Table S5: Bifidobacterial ITS profiling quality filtering table.

Sample Group	Time Point	Mouse ID	Sample	Number of sequenced pe reads	Number of pe reads with mean quality > 20	Number of merged pe reads	Non-bacterial sequences	Length outside bounds of 100 and 400	Ambiguous bases	Homopolymers > 7	Mismatch in primers >1	Reverse primer not found	Final Read Number
CG	T_0	1	**WT-1A**	66035	18876	18379	3	0	0	3	18339	0	34
CG	T_1	1	**WT-1B**	98615	34299	32444	8	0	0	19	20369	31	12017
CG	T_2	1	**WT-1C**	73988	51760	49788	2	0	0	22	47801	6	1957
CG	T_3	1	**WT-1D**	45607	44248	43591	15	0	0	4	43570	0	2
CG	T_0	2	**WT-2A**	54386	45788	44931	0	0	0	3	44863	0	65
CG	T_1	2	**WT-2B**	98616	89769	86132	4	0	1	20	86034	1	72
CG	T_2	2	**WT-2C**	82401	45412	43362	0	0	1	16	35016	18	8311
CG	T_3	2	**WT-2D**	73244	65894	63166	2	0	2	21	60367	3	2771
CG	T_0	3	**WT-3A**	86982	37379	36115	0	0	0	13	31556	8	4538
CG	T_1	3	**WT-3B**	92483	71691	69559	0	0	0	17	67305	5	2232
CG	T_2	3	**WT-3C**	14401	712	662	0	0	0	0	264	1	397
CG	T_3	3	**WT-3D**	388903	378383	370507	19	0	0	52	370419	0	17
CG	T_0	4	**WT-4A**	56149	54126	52848	0	0	0	6	52833	0	9
CG	T_1	4	**WT-4B**	6760	4220	4124	1	0	0	1	4043	0	79
CG	T_2	4	**WT-4C**	75216	38348	36517	3	0	0	16	29327	15	7156
CG	T_3	4	**WT-4D**	36569	34990	34373	2	0	0	1	34366	0	4
CG	T_0	5	**WT-5A**	36637	28179	27615	3	0	0	7	27368	0	237
CG	T_1	5	**WT-5B**	48916	46923	46173	0	0	0	7	46161	0	5
CG	T_2	5	**WT-5C**	78437	57089	54647	4	0	0	24	52169	4	2446
CG	T_3	5	**WT-5D**	91571	87069	83778	2	0	2	37	83734	0	3
CG	T_0	6	**WT-6A**	50907	49533	48799	0	0	0	10	48789	0	0
CG	T_1	6	**WT-6B**	69118	62739	60988	5	0	1	11	59375	4	1592
CG	T_2	6	**WT-6C**	37208	34867	33415	2	0	0	12	32704	1	696
CG	T_3	6	**WT-6D**	56051	40953	39941	3	0	0	12	34265	15	5646
CG	T_0	7	**WT-7A**	68553	66340	63749	7	0	0	28	63709	0	5
CG	T_1	7	**WT-7B**	84075	67249	64546	6	0	2	20	47062	31	17425
CG	T_2	7	**WT-7C**	69716	65781	62979	6	0	1	17	53370	24	9561
CG	T_3	7	**WT-7D**	80578	36832	35309	4	0	1	7	34440	1	856
CG	T_0	8	**WT-8A**	96574	85440	81774	4	0	0	25	53481	52	28212
CG	T_1	8	**WT-8B**	83792	77585	73383	7	0	1	25	67660	18	5672
CG	T_2	8	**WT-8C**	69987	66990	64550	2	0	0	12	61214	10	3312
CG	T_3	8	**WT-8D**	54056	48141	46939	1	0	0	13	41463	13	5449
Breve2L	T_0	9	**WT-9A**	58274	57001	56034	1	0	0	9	56024	0	0
Breve2L	T_1	9	**WT-9B**	75468	73341	70434	2	0	1	21	68139	3	2268
Breve2L	T_2	9	**WT-9C**	77640	72101	69065	5	0	0	21	68469	1	569
Breve2L	T_3	9	**WT-9D**	71572	69446	66973	2	0	0	19	65605	6	1341

Breve2L	T_0	10	**WT-10A**	40653	25471	25004	0	0	0	5	24690	0	309
Breve2L	T_1	10	**WT-10B**	40673	37197	36643	0	0	0	2	36621	0	20
Breve2L	T_2	10	**WT-10C**	76059	70819	68166	6	0	1	15	64453	3	3688
Breve2L	T_3	10	**WT-10D**	43732	41020	40448	1	0	0	4	40379	0	64
Breve2L	T_0	11	**WT-11A**	47837	46408	45646	1	0	0	5	45640	0	0
Breve2L	T_1	11	**WT-11B**	45754	42505	41815	1	0	0	6	41646	1	161
Breve2L	T_2	11	**WT-11C**	59288	49716	47430	3	0	1	14	30729	16	16666
Breve2L	T_3	11	**WT-11D**	85559	82388	79002	5	0	1	24	78881	0	91
Breve2L	T_0	12	**WT-12A**	67438	60985	58690	3	0	1	21	57975	1	689
Breve2L	T_1	12	**WT-12B**	82596	80133	76799	3	0	0	19	68027	18	8732
Breve2L	T_2	12	**WT-12C**	80399	60190	57938	3	0	0	36	56605	3	1291
Breve2L	T_3	12	**WT-12D**	82495	44987	43474	3	0	0	46	27629	31	15765
Breve2L	T_0	13	**WT-13A**	31999	30781	30302	0	0	0	3	30296	0	3
Breve2L	T_1	13	**WT-13B**	47635	45354	44605	0	0	0	5	44600	0	0
Breve2L	T_2	13	**WT-13C**	67693	41991	41165	1	0	0	7	38480	4	2673
Breve2L	T_3	13	**WT-13D**	67327	60740	59116	1	0	0	5	51780	15	7315
Breve2L	T_0	14	**WT-14A**	79958	70737	68050	2	0	2	13	64376	8	3649
Breve2L	T_1	14	**WT-14B**	44561	43302	42637	0	0	0	7	42620	0	10
Breve2L	T_2	14	**WT-14C**	68888	63176	60740	0	0	2	13	59304	0	1421
Breve2L	T_3	14	**WT-14D**	60063	57395	56546	1	0	0	8	56381	2	154
Breve2L	T_0	15	**WT-15A**	73127	66097	63949	5	0	0	19	56638	5	7282
Breve2L	T_1	15	**WT-15B**	38841	37864	37265	0	0	0	3	37197	2	63
Breve2L	T_2	15	**WT-15C**	72741	64041	61895	7	0	1	15	59284	2	2586
Breve2L	T_3	15	**WT-15D**	45916	42439	41839	5	0	0	6	41789	0	39
Breve2L	T_0	16	**WT-16A**	42058	41448	40786	2	0	0	15	40769	0	0
Breve2L	T_1	16	**WT-16B**	50832	50305	49528	2	0	0	9	49517	0	0
Breve2L	T_2	16	**WT-16C**	72101	64159	61882	3	0	1	15	60660	1	1202
Breve2L	T_3	16	**WT-16D**	58222	15376	14870	0	0	0	3	11287	6	3574
CG+Breve2L	T_0	17	**WT-17A**	63236	57686	56111	1	0	0	19	53627	10	2454
CG+Breve2L	T_1	17	**WT-17B**	88868	22821	21212	7	0	0	3	11574	25	9603
CG+Breve2L	T_2	17	**WT-17C**	63726	57941	55777	2	0	1	25	51254	13	4482
CG+Breve2L	T_3	17	**WT-17D**	60263	58201	57420	1	0	0	6	57320	0	93
CG+Breve2L	T_0	18	**WT-18A**	48706	47370	46624	0	0	0	8	46615	0	1
CG+Breve2L	T_1	18	**WT-18B**	64647	55448	53074	6	0	1	35	49009	7	4016
CG+Breve2L	T_2	18	**WT-18C**	100580	68501	65721	2	0	1	30	60323	11	5354
CG+Breve2L	T_3	18	**WT-18D**	68705	49021	48177	3	0	0	5	46937	1	1231
CG+Breve2L	T_0	19	**WT-19A**	97380	56758	54411	3	0	1	31	49896	9	4471
CG+Breve2L	T_1	19	**WT-19B**	79803	31571	30129	3	0	0	7	24583	11	5525
CG+Breve2L	T_2	19	**WT-19C**	100801	34207	32412	9	0	0	10	29829	6	2558
CG+Breve2L	T_3	19	**WT-19D**	53220	49324	48592	3	0	0	11	48531	0	47
CG+Breve2L	T_0	20	**WT-20A**	49572	48891	48021	3	0	0	8	48010	0	0
CG+Breve2L	T_1	20	**WT-20B**	68842	67185	64708	2	0	1	15	62580	5	2105
CG+Breve2L	T_2	20	**WT-20C**	98311	27744	26223	2	0	0	15	17465	24	8717
CG+Breve2L	T_3	20	**WT-20D**	68057	60460	58295	0	0	1	11	57491	1	791
CG+Breve2L	T_0	21	**WT-21A**	65884	51934	49899	4	0	0	16	49357	0	522

CG+Breve2L	T_1	21	**WT-21B**	80559	72372	69388	3	0	4	22	63686	12	5661
CG+Breve2L	T_2	21	**WT-21C**	122242	37689	36656	0	0	0	9	30795	15	5837
CG+Breve2L	T_3	21	**WT-21D**	59304	56332	55246	1	0	0	9	54060	2	1174
CG+Breve2L	T_0	22	**WT-22A**	34663	33463	32967	3	0	0	4	32959	0	1
CG+Breve2L	T_1	22	**WT-22B**	73540	31478	30237	5	0	1	11	28428	3	1789
CG+Breve2L	T_2	22	**WT-22C**	82779	44256	41862	7	0	1	21	32448	22	9363
CG+Breve2L	T_3	22	**WT-22D**	69278	67516	64883	2	0	1	22	63666	1	1191
CG+Breve2L	T_0	23	**WT-23A**	71319	38398	36714	3	0	3	19	30311	9	6369
CG+Breve2L	T_1	23	**WT-23B**	74124	52337	49905	10	0	1	17	32407	23	17447
CG+Breve2L	T_2	23	**WT-23C**	55717	19551	19109	0	0	0	21	17943	4	1141
CG+Breve2L	T_3	23	**WT-23D**	63689	57801	56833	5	0	0	3	55144	2	1679
CG+Breve2L	T_0	24	**WT-24A**	39593	38894	38263	1	0	0	4	38258	0	0
CG+Breve2L	T_1	24	**WT-24B**	72335	61201	58771	5	0	0	10	54806	6	3944
CG+Breve2L	T_2	24	**WT-24C**	46807	19644	19191	1	0	0	0	18180	5	1005
CG+Breve2L	T_3	24	**WT-24D**	74482	45334	44355	0	0	0	6	39231	12	5106
